# Neutrophil-to-lymphocyte ratio predicts a poor prognosis for penile cancer with an immunosuppressive tumor microenvironment

**DOI:** 10.3389/fimmu.2025.1568825

**Published:** 2025-04-16

**Authors:** Xingliang Tan, Yanjun Wang, Yiqi Yu, Runhao Zheng, Jing Li, Shaohua Chen, Qingling Xie, Shengjie Guo, Chichen Zhang, Xinpei Deng, Zhicheng Liu, Yi Tang, Hang Li, Weicheng Wu, Juexiao Chen, Qianghua Zhou, Wensu Wei, Kai Yao, Zhiming Wu

**Affiliations:** ^1^ Department of Urology, Sun Yat-sen University Cancer Center, Guangzhou, China; ^2^ State Key Laboratory of Oncology in Southern China, Guangzhou, China; ^3^ Guangdong Provincial Clinical Research Center for Cancer, Guangzhou, China; ^4^ Zhongshan School of Medicine, Sun Yat-Sen University, Guangzhou, China; ^5^ Department of Urology, Affiliated Cancer Hospital and Institute of Guangzhou Medical University, Guangzhou, China; ^6^ Department of Urology, Guangxi Medical University Cancer Center, Nanning, China; ^7^ Department of Urology, The Fifth Affiliated Hospital of Guangzhou Medical University, Guangzhou Medical University, Guangzhou, China

**Keywords:** cancer, neutrophil-to-lymphocyte ratio, tumor-associated neutrophils, neutrophil extracellular traps, propensity score matching

## Abstract

**Background:**

Chronic inflammation related to poor genital hygiene is a well-recognized pathogenic trigger for penile cancer (PC). The neutrophil-to-lymphocyte ratio (NLR) is a simple, reproducible systemic inflammatory marker and has been reported to indicate unfavorable outcomes. However, previous studies were limited by small sample sizes, confounding prognostic factors and a lack of high-quality evidence demonstrating the significance of the NLR in PC.

**Methods:**

A large multicenter cohort of 582 PC patients who underwent radical inguinal lymphadenectomy with definitive pN stage information was assessed. Univariate and multivariate Cox regression analyses were performed to investigate the prognostic value of inflammation-related markers. Propensity score matching (PSM) was used to minimize confounding prognostic clinicopathological features. Immunofluorescence was used to assess the immunosuppressive tumor microenvironment (TME).

**Results:**

A high preoperative NLR (≥ 3.0) was associated with advanced pT, pN, and pathological grade and lymphovascular invasion in PC patients. After PSM to eliminate interference from clinical factors, pN and the NLR were found to be independent prognostic indicators (both p<0.001). PC patients with high NLRs had shorter progression-free survival (PFS) and poorer cisplatin-based chemotherapy and PD-1 immunotherapy response. We also found that the NLR is associated with proinflammatory cytokine secretion and increased N2 tumor-associated neutrophils (TANs) infiltration and CD8^+^ T-cell exhaustion in TME. N2 TANs induced neutrophil extracellular trap formation might contribute to tumor progression and resistance in high-NLR PC patients.

**Conclusions:**

The NLR is an effective, simple and independent prognostic indicator for PC. A high NLR is associated with an immunosuppressive TME and poor outcomes.

## 1 Background

Penile cancer (PC) is a physically and mentally devastating malignancy in males and is particularly prevalent in developing countries with poor economic and hygienic conditions (1, 2). According to the Global Cancer Observatory, the worldwide age-standardized incidence rate of PC was 0.80 cases per 100,000 person-years in 2018, with an expected increase of over 56% by 2040 (1). The prediction of PC prognosis is mainly based on the tumor–node–metastasis (TNM) staging system and pathological characteristics (3, 4). Lymph node metastasis is recognized as a key risk factor in PC, with dramatic decreases in 5-year overall survival (OS) rates of 79.7–31.8% for pN1–pN3 patients and 93.6% for pN0 patients (5–7); however, molecular signatures for individualized prognosis assessment are lacking. Although several markers (8–11), including squamous cell carcinoma antigen (SCC-Ag) expression, HER2 overexpression, and EGFR amplification, have been reported to be correlated with survival, their low sensitivity has limited their clinical application; thus, prognostic indicators, especially for preoperative assessment, are still lacking.

Human papilloma virus infection, chronic irritation, and inflammation are key factors in PC tumorigenesis and progression (12). Chronic inflammation is responsible for cancer incidence and controlling inflammation is a potential strategy to stop the development of cancer (13). The neutrophil-to-lymphocyte ratio (NLR) has been recognized as an indicator of systemic inflammation associated with advanced disease, poor outcomes and chemotherapy resistance in a series of solid tumors (14–17). The NLR serves as a simple, reproducible and economical marker that is related to lymph node metastasis, immunotherapy response, and survival in PC patients (16, 18–21). The high level of NLR is associated with tumor-associated neutrophils (TANs), mediating the suppressive immune microenvironment, and is related to poor outcomes. However, previous studies have not only ignored the dominant influence of lymph node metastasis and other confounding factors on NLR-based prognostic assessment but have also been limited by small sample sizes, the use of single-center cohorts, a lack of standardized inguinal lymph node dissection and the use of inappropriate statistical methods (18–20, 22). In addition, the relationship between the peripheral NLR and the inflammatory microenvironment of tumors remains unclear.

In our study, we addressed these limitations by conducting a multicenter, large-scale, propensity score matching (PSM) analysis of 582 PC patients with clear pN stage information to demonstrate that the NLR serves as an independent predictor of survival in PC patients. Additionally, we investigated the correlations between the NLR and the levels of serum inflammatory cytokines, secreted proteins and tumor-infiltrating immune cells (TIICs). We found that a high preoperative NLR affected the immunosuppressive tumor microenvironment (TME) by increasing N2-type tumor-associated neutrophil (TAN) infiltration, which induced the formation of neutrophil extracellular traps (NETs) to promote tumor progression, contributing to a poor prognosis in PC patients.

## 2 Methods

### 2.1 Patient cohorts and research ethics

This retrospective study was conducted on 582 pathologically confirmed PSCC patients between January 2010 and December 2023 from four centers; this cohort included 495 (85.1%) patients from Sun Yat-sen University Cancer Center (SYSUCC), and the detailed information is listed in **Table 1**. The inclusion criteria were as follows: 1. Naive patients who underwent bilateral and radical inguinal lymphadenectomy (rILND) with classical boundaries (23, 24). 2. Clear pN stage information individually determined by experienced pathologists on the basis of the 8th edition of the AJCC TNM Staging System for Penile Cancer (2). 3. Reliable preoperative routine blood, blood chemistry and inflammatory secreted proteins and cytokines. 4. Clear clinical outcome data. Our study received approval from the ethics committee of Sun Yat-sen University Cancer Center (Approval number: G2023-098-01), and informed consent was waived by the institutional review board due to the retrospective nature of the investigation.

**Table 1:** Clinicopathological characteristics of 582 penile cancer patients.

Variables (n = 582)	
Institution	
SYSUCC	495 (85.1%)
ACHIGMU	41 (7.0%)
GMUCC	36 (6.2%)
FAHGMU	10 (1.7%)
Age (years)	
> 55	290 (49.8%)
≤ 55	292 (50.2%)
Body mass index (kg/m^2^)	
> 23	266 (45.7%)
≤ 23	274 (47.1%)
Unknown	42 (7.2%)
pT stage	
pTa/Tis	26 (4.4%)
pT1	209 (35.9%)
pT2	124 (21.3%)
pT3	187 (32.1%)
pT4	14 (2.4%)
pTx	22 (3.7%)
pN stage	
pN0	312 (53.6%)
pN1	75 (12.8%)
pN2	55 (9.4%)
pN3	140 (24.0%)
Grade	
G1	284 (48.7%)
G2	212 (36.4%)
G3/4	64 (10.9%)
Gx	22 (3.7%)
Pathologic high-risk factors	
Lymphovascular invasion	62 (10.6%)
Perineural invasion	89 (15.2%)
Extranodal extension	105 (18.0%)
Inflammatory indicators (Median, IQR)	
NLR (n = 582)	2.56 (1.82-3.75)
LMR (n = 582)	3.69 (2.63-5.00)
CRP (n = 559)	2.40 (0.88-9.56)
SAA (n = 412)	6.75 (3.60-23.03)
Perioperative therapy	
Cisplatin-based chemotherapy	205 (35.2%)
Chemotherapy + anti-PD-1 immunotherapy	107 (18.3%)
Clinical Outcomes	
Progression	162 (27.8%)
Death due to disease	124 (21.3%)

SYSUCC, Sun Yat-sen University Cancer Center; ACHIGMU, Affiliated Cancer Hospital and Institute of Guangzhou Medical University; FAHGMU, Fifth Affiliated Hospital of Guangzhou Medical University; GMUCC, Guangxi Medical University Cancer Center.

### 2.2 Propensity score matching and cutoff values

PSM was performed with a 1:1 matching ratio, using the “nearest” method and a caliper of 0.2. The matched variables included age, pT stage, pN stage, histological grade, body mass index (BMI), and the presence of lymphovascular invasion (LVI), which were significantly different between the high and low NLR groups. For the PSM population, we used the chi-square test and absolute standardized difference test to assess the balance of the variables between the two groups. The primary endpoints for this analysis were cancer-specific survival (CSS) and progression-free survival (PFS). A receiver operating characteristic (ROC) curve was generated to determine the optimal cutoff values of continuous variables, including the NLR, lymphocyte–monocyte ratio (LMR), C-reactive protein (CRP), and serum amyloid A (SAA).

### 2.3 Evaluation of tumor immune microenvironment scores

To determine the composition of inflammatory immune cells and immune microenvironment heterogeneity across groups classified according to the NLR, 16 fresh tumor tissue samples (9 from the high NLR group and 7 from the low NLR group) were subjected to mRNA-seq. The Immune Cell Abundance Identifier (ImmuCellAI) tool was used to estimate the abundance of 24 immune cell types from the mRNA-seq matrix and provided a comprehensive landscape of inflammatory immune cells for high- and low-NLR tumors (25).

### 2.4 Laboratory assessments of inflammatory markers

The NLR and LMR and CRP, SAA and SCC-Ag levels were assessed before surgery. SAA detection was only conducted after 2017, so data on SAA were collected from only 412 patients. Serum proinflammatory cytokines (26), including interleukin-6 (IL-6) and IL-8, and anti-inflammatory cytokines (IL-4 and IL-10) were detected in 116 PC patients via enzyme-linked immunosorbent assay (ELISA) (Human ELISA Kit, Beyotime, #PI618, #PI330, #PI640 and #PI528). The specific steps are described in the kit instructions.

### 2.5 Hematoxylin and eosin staining and immunohistochemistry

A total of 167 PC patients with well-preserved, 4-µm paraffin-embedded tumor sections were included in the analysis. HE-stained samples were used to evaluate the proportion of TIICs. CD4+ T cells (CD4, CST, #25229), CD8+ T cells (CD8α, CST, #98941) and tumor-associated neutrophils (TANs) (CD66b, Abcam, #ab300122) were detected via IHC staining according to standard pathologic procedures previously described (27, 28). Five fields of view at high-magnification (400×) were randomly selected to count the absolute cell number of immune cells stained with each antibody. Pathological diagnosis and IHC staining were performed by two independent pathologists.

### 2.6 Flow cytometry and multiplex immunofluorescence

A total of 42 fresh tumor tissues were obtained and prepared as single-cell suspensions for subsequent flow cytometry. In brief, N2 TANs were identified according to dual positivity for CXCR2 (anti-CXCR2 antibody, Abcam, ab89254) and CD66b (anti-CD66b antibody, Abcam, ab48589) (29, 30). Flow cytometry was restricted to sorting double-positive cells, and the percentage of N2-phenotype cells among all TANs (CD66b+) was calculated. Multiplex immunofluorescence was performed with a Cellcook kit according to the manufacturer’s instructions. The antibodies against the following targets were used: pan-CK (CST, #67306), CD66b (Abcam, ab48589), CXCR2 (Abcam, ab89254), myeloperoxidase (MPO) (CST, #14569), TGF-β (CST, #3709), TIM3 (CST, #75743) and (citH3) (CST, #97272). The visualization of NETs was performed according to MPO and citH3 staining (31). CD66b/CXCR2 and TIM3/CD8 were colocalized in N2 TANs and exhausted CD8+ T cells, respectively. The spatial distance between immune cells was analyzed via Halo software (Indica labs) and multiplex IHC modules.

### 2.7 Statistical analysis

Statistical analysis was conducted via SPSS software (Ver. 25.0). All the results are presented as the means ± SDs, and the differences between two groups were assessed via Student’s t test or one-way ANOVA. Pearson correlation analysis was used to detect correlations between variables. Survival analysis, including PFS and CSS analysis, was performed using Kaplan–Meier survival curves, and multivariate analysis was performed via the forward method. A p value < 0.05 was considered to indicate statistical significance.

## 3 Results

### 3.1 Baseline characteristics

A total of 582 penile squamous cell carcinoma (PSCC) patients from four centers were enrolled in our study, and the mean follow-up time was 41.1 ± 31.4 months (**Table 1**). All patients underwent bilateral radical dissection of inguinal lymph nodes (13.12 ± 5.42 nodes per patient) (23). Among the patients, 46.4% of patients had positive nodes, and 12.8%, 9.4% and 24.0% of patients had clear pN1, pN2 and pN3 stage disease, respectively. Among these patients, 105 (18.0%) had extranodal extension (**Table 1**). Among pN+ patients, cisplatin-based chemotherapy combined with anti-PD-1 immunotherapy was administered to 205 patients (35.2%) and 107 patients (18.3%). Ultimately, 162 patients (27.8%) exhibited disease progression, and 124 patients died (21.3%) from PSCC; the 5-year PFS and 5-year CSS rates were 68.5% (95% CI: 0.64–0.73) and 75.0% (95% CI: 0.71–0.79), respectively, in our cohort.

### 3.2 Cutoff values of inflammatory markers and SCC-Ag

**Figure 1:**
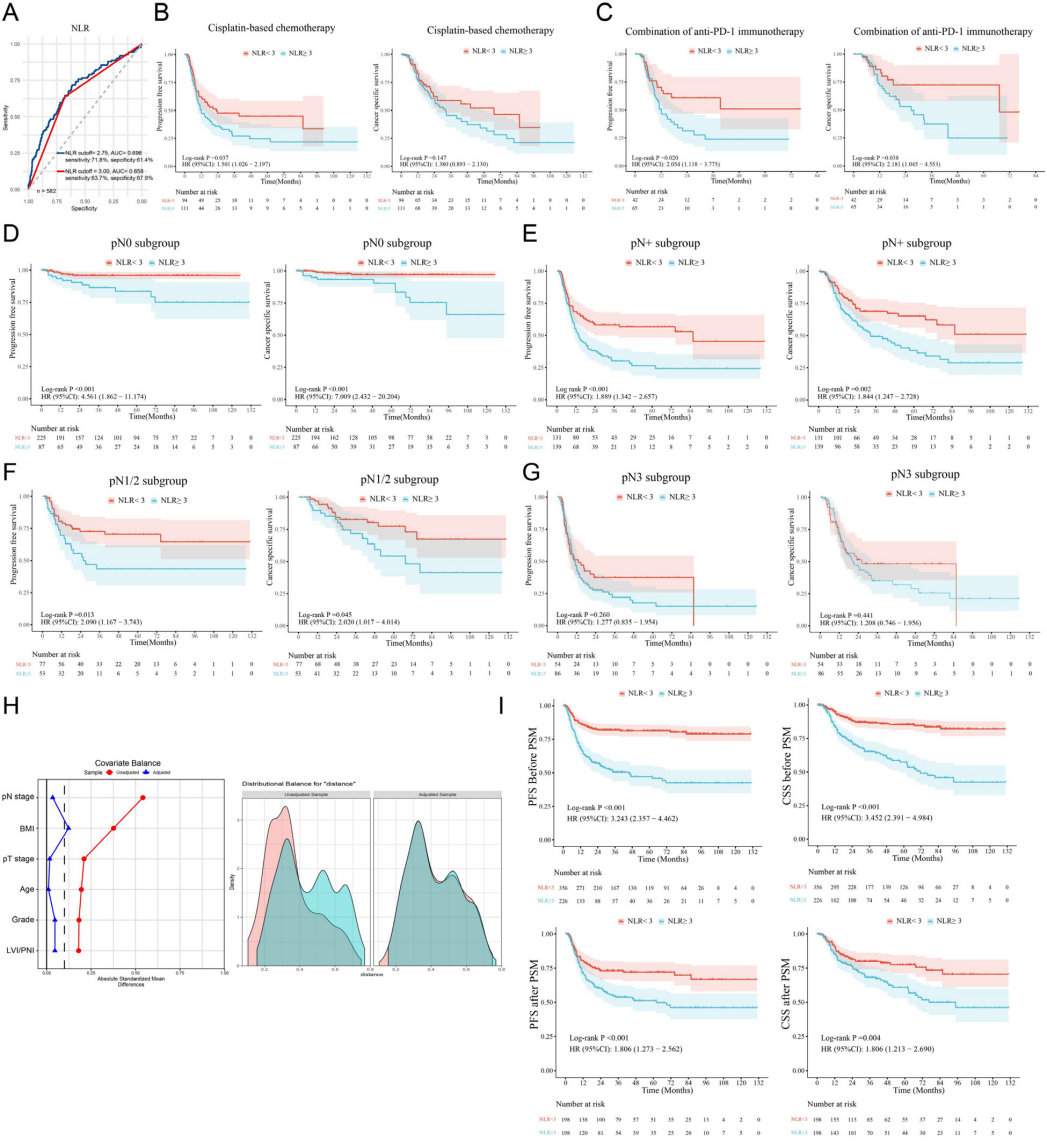
A high NLR is associated with a poor PSCC prognosis. **(A)** ROC curves indicated that the best cutoff value of the NLR for determining cancer-specific survival in PSCC patients was 2.75 in our cohort (blue line). The red line shows that the AUC was 0.658 when the cutoff value of the NLR was 3.00. **(B, C)** Subgroup analysis was performed on patients treated with therapeutic agents, and survival analysis revealed that high NLRs were related to poor response to chemotherapy and immunotherapy. **(D–G)** Survival analysis was performed for the pN0, pN+, pN1-N2 and pN3 subgroups. **(H)** PSM was used to adjust for confounding clinicopathological effects in the high/low NLR groups. **(I)** Differences in survival between the high- and low-NLR groups before and after PSM. NLR, neutrophil–lymphocyte ratio; ROC, receiver operating characteristic curve; AUC, area under the curve; PFS, progression-free survival; CSS, cancer-specific survival; PSM, propensity score matching; HR, hazard ratio; Cl, confidence interval; LVI, lymphovascular invasion; PNI, perineural invasion; PSCC, penile squamous cell carcinoma.

Preoperative inflammatory indicators, including the NLR and LMR and the levels of CRP, SAA, and SCC-Ag were assessed; the median values are listed in **Table 1**. The optimal cutoff values on the basis of the ROC curves were 3.5 for the LMR, 2.2 for CRP, 11.3 for SAA and 2.7 for SCC-Ag (**Supplementary Figure S1**). Although the cutoff value for the NLR was 2.75 in our cohort, the threshold was recalibrated to 3.0 to maintain consistency with previous literature and improve reproducibility and clinical practicality (**Figure 1A**) (20, 21). In total, a high NLR (NLR≥ 3) was detected in 38.8% of patients, and the NLR has a sensitivity of 63.7% and a specificity of 67.9% for predicting survival (**Figure 1A**).

### 3.3 Inflammatory markers and clinical outcomes

To further explore the clinical significance of inflammatory indicators in survival, Kaplan–Meier survival analysis was performed (**Supplementary Table S1**). Univariate analysis revealed that advanced pT, pN, and pathological grade; LVI/PNI; high NLR; high CRP, SAA and SCC-Ag levels; and low LMR were associated with poor CSS and PFS (**Supplementary Table S1** and **Supplementary Figure S2**). More importantly, we found that a high NLR in was associated with a poor response to cisplatin-based chemotherapy and anti-PD-1 immunotherapy in PSCC patients (**Figures 1B, C**). Further multivariate analysis revealed that only pN stage was an independent unfavorable prognostic factor (**Supplementary Table S2**). Similarly, our study indicated that lymph node metastasis was a radical and recognized prognostic factor for predicting clinical outcomes (7); thus, it might be a confounding factor in survival analysis based on the NLR that has been ignored in previous NLR studies.

### 3.4 Subgroup analysis and propensity score matching for the NLR

To control the confounding influence of lymph node metastasis in prognosis analysis, we first conducted subgroup analysis between different pN stages. We found that, in both the pN0 subgroup and the pN+ subgroup, PSCC patients with high preoperative NLRs had shorter PFS and CSS than those with low NLRs did (both p < 0.01) (**Figures 1D, E**). In patients with pN1/N2-stage disease and a low metastatic burden, the NLR was also associated with poor outcomes (**Figure 1F**), but the differences between patients with advanced (pN3-stage) disease were not statistically significant (**Figure 1G**).

Subsequently, the chi-square test demonstrated that a high NLR was significantly correlated with age, body mass index (BMI), pT stage, pN stage, pathological grade and LVI/PNI (**Table 2**). To balance the above confounding factors, we constructed a 1:1 cohort (high NLR versus low NLR, with 198 patients in each group) via the PSM method. The baseline characteristics were comparable between the two groups (**Table 2** **).** PSM analysis confirmed that the covariates were well balanced, as evidenced by absolute standard differences of less than 0.1 for all included factors (**Figure 1H**). To our surprise, the survival differences between the high- and low-NLR groups persisted; notably, PFS and CSS were significantly longer in the low-NLR group (**Figure 1I**). After the clinicopathological factors were adjusted via PSM, univariate and multivariate analyses revealed that pN stage and a high NLR were both independent prognostic indicators in terms of PFS (HR: 1.64; 95% CI: 1.15–2.34) and CSS (HR: 1.56; 95% CI: 1.04–2.34) (**Table 3**).

**Table 2:** Association between the NLR and clinicopathological features before and after propensity score matching.

Variables	Before PSM					After PSM				
	Total (n=582)	NLR<3 (n=356)	NLR≥3 (n=226)	χ^2^	P value	Total (n=396)	NLR<3 (n=198)	NLR≥3 (n=198)	χ^2^	P value
**Age, n (%)**				5.187	**0.023**				0.010	0.920
≤ 55	292 (50.17)	192 (53.93)	100 (44.25)			191 (48.23)	96 (48.48)	95 (47.98)		
> 55	290 (49.83)	164 (46.07)	126 (55.75)			205 (51.77)	102 (51.52)	103 (52.02)		
**BMI, n (%)**				9.473	**0.009**				1.803	0.406
≤ 23	274 (47.08)	161 (45.22)	113 (50.00)			198 (50)	102 (51.52)	96 (48.48)		
> 23	266 (45.7)	160 (44.94)	106 (46.90)			188 (47.47)	93 (46.97)	95 (47.98)		
MD	42 (7.22)	35 (9.83)	7 (3.10)			10 (2.53)	3 (1.52)	7 (3.54)		
**pT stage, n (%)**				12.653	**0.013**				1.799	0.773
pTa/Tis/pT1	235 (40.38)	158 (44.38)	77 (34.07)			150 (37.88)	74 (37.37)	76 (38.38)		
pT2	124 (21.31)	80 (22.47)	44 (19.47)			82 (20.71)	44 (22.22)	38 (19.19)		
pT3	187 (32.13)	102 (28.65)	85 (37.61)			136 (34.34)	68 (34.34)	68 (34.34)		
pT4	14 (2.41)	5 (1.40)	9 (3.98)			12 (3.03)	4 (2.02)	8 (4.04)		
pTx	22 (3.78)	11 (3.09)	11 (4.87)			16 (4.04)	8 (4.04)	8 (4.04)		
**pN stage, n (%)**				46.373	**<0.001**				1.159	0.763
pN0	312 (53.61)	225 (63.20)	87 (38.50)			175 (44.19)	88 (44.44)	87 (43.94)		
pN1	75 (12.89)	46 (12.92)	29 (12.83)			58 (14.65)	29 (14.65)	29 (14.65)		
pN2	55 (9.45)	31 (8.71)	24 (10.62)			50 (12.63)	28 (14.14)	22 (11.11)		
pN3	140 (24.05)	54 (15.17)	86 (38.05)			113 (28.54)	53 (26.77)	60 (30.30)		
**Grade, n (%)**				11.724	**0.020**				3.287	0.511
G1	284 (48.8)	189 (53.09)	95 (42.04)			179 (45.2)	92 (46.46)	87 (43.94)		
G2	212 (36.43)	127 (35.67)	85 (37.61)			152 (38.38)	78 (39.39)	74 (37.37)		
G3	61 (10.48)	28 (7.87)	33 (14.60)			47 (11.87)	20 (10.10)	27 (13.64)		
G4	3 (0.52)	1 (0.28)	2 (0.88)			2 (0.51)	0 (0.00)	2 (1.01)		
Gx	22 (3.78)	11 (3.09)	11 (4.87)			16 (4.04)	8 (4.04)	8 (4.04)		
**LVI/PNI, n (%)**				5.178	**0.023**				0.230	0.631
No	463 (79.55)	294 (82.58)	169 (74.78)			306 (77.27)	151 (76.26)	155 (78.28)		
Yes	119 (20.45)	62 (17.42)	57 (25.22)			90 (22.73)	47 (23.74)	43 (21.72)		

PSM, propensity score matching; MD, missing data; LVI, lymphovascular invasion; PNI, perineural invasion; NLR, neutrophil–lymphocyte ratio.

The bold-faced values represent P-values that indicate statistical significance.

**Table 3:** Univariate and multivariate analyses of clinicopathological factors associated with survival after PSM.

Variables	PFS after PSM				CSS after PSM			
	Univariate analysis		Multivariate analysis		Univariate analysis		Multivariate analysis	
	HR (95% Cl)	P value	HR (95% Cl)	P value	HR (95% Cl)	P value	HR (95% Cl)	P value
**Age (>55 vs. ≤55)**	0.85 (0.60-1.19)	0.350	1.21 (0.85-1.73)	0.283	0.95 (0.64-1.40)	0.790	1.42 (0.95-2.13)	0.087
**BMI (>23 vs. ≤23)**	1.03 (0.92-1.14)	0.640	1.09 (0.97-1.23)	0.152	1.03 (0.93-1.15)	0.564	1.11 (0.98-1.25)	0.087
pT stage^a^	1.17 (1.07-1.27)	**<0.001**	1.12 (0.95-1.31)	0.179	1.14 (1.03-1.26)	0.013	1.07 (0.89-1.28)	0.495
pN stage^a^	2.22 (1.90-2.58)	**<0.001**	2.22 (1.89-2.60)	**<0.001**	2.22 (1.86-2.65)	**<0.001**	2.28 (1.89-2.75)	**<0.001**
Pathological grade^a^	1.13 (1.04-1.24)	0.006	0.99 (0.85-1.17)	0.929	1.10 (0.99-1.23)	0.084	0.97 (0.81-1.17)	0.759
**LVI/PNI (Yes vs. No)**	1.55 (1.06-2.27)	0.023	1.12 (0.76-1.66)	0.562	1.66 (1.09-2.55)	**0.019**	1.25 (0.81-1.94)	0.320
**NLR (≥ 3.0 vs. < 3.0)**	1.81 (1.27-2.56)	**<0.001**	1.64 (1.15-2.34)	**0.006**	1.81 (1.21-2.69)	**0.004**	1.56 (1.04-2.34)	**0.030**

a pT, pN and pathological grade used linear trends to compare different subgroups. PSM, propensity score matching; PFS, progression-free survival; CSS, cancer-specific survival; HR, hazard ratio; 95% CI, 95% confidence interval; LVI, lymphovascular invasion; PNI, perineural invasion; NLR, neutrophil–lymphocyte ratio.

The bold-faced values represent P-values that indicate statistical significance.

### 3.5 A high NLR is associated with increased systemic inflammation and TAN infiltration

**Figure 2:**
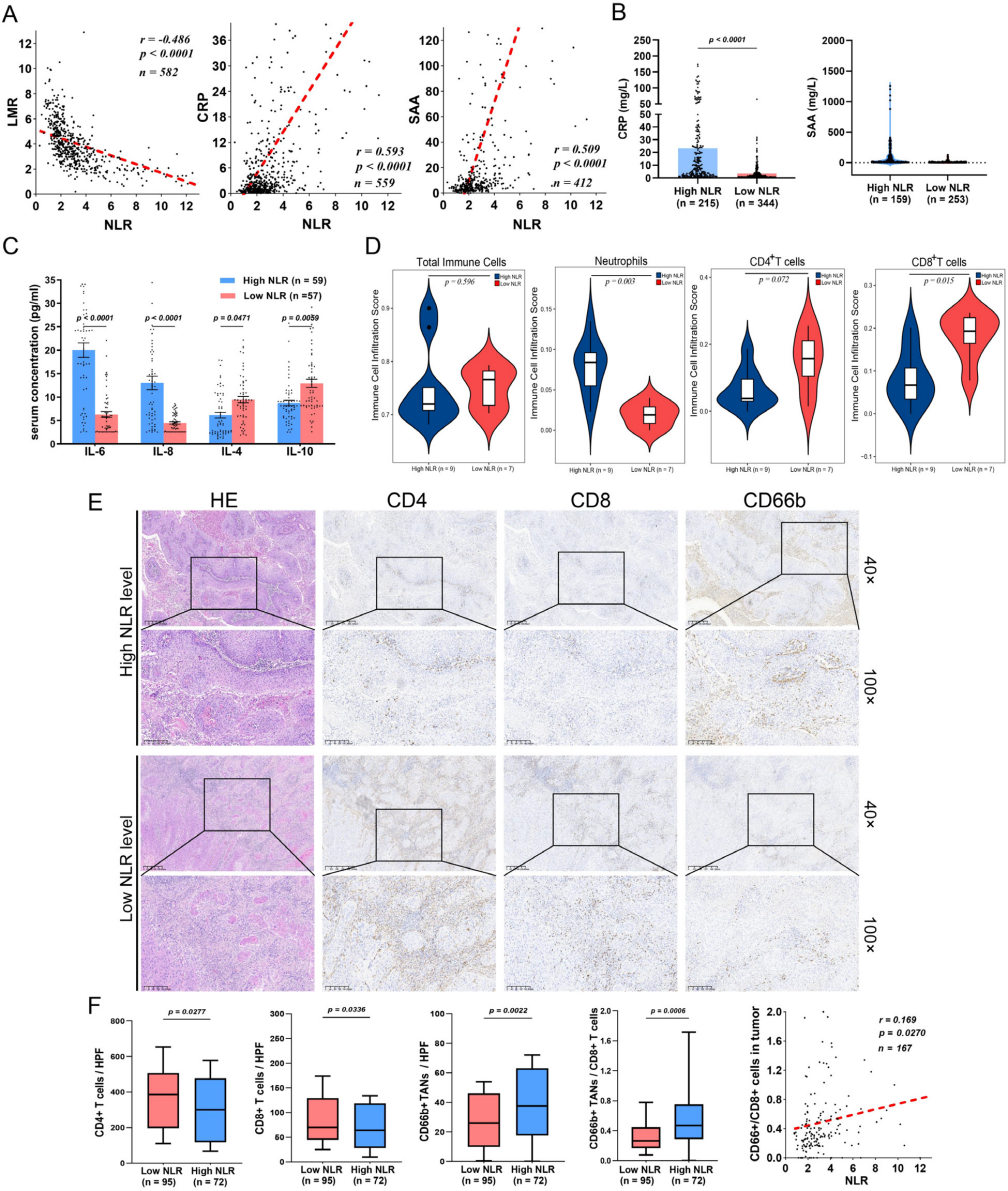
A high preoperative NLR is associated with TAN infiltration and a decrease in CD8+ T cells in the TME. **(A)** In PSCC patients, the NLR is negatively correlated with LMR and positively correlated with proinflammatory CRP and SAA protein expression. **(B)** CRP and SAA were evaluated in the high-NLR groups. **(C)** The NLR is related to high proinflammatory cytokine secretion (IL-6 and IL-8) and low levels of anti-inflammatory cytokines (IL-4 and IL-10) in PSCC. **(D)** Immune infiltration scores determined by ImmuCellAI indicated that the infiltration of TANs was increased, whereas the infiltration of CD8+ T cells was decreased in the high-NLR groups. **(E)** IHC assays showing the expression patterns of TANs (CD66^+^), CD4^+^ T cells and CD8^+^ T cells in the TME. **(F)** CD4^+^ T cells and CD8^+^ T cells were abundant in the low NLR subgroup, whereas TAN infiltration and the TANs/CD8^+^ T-cell ratio were increased and positively correlated with the high NLR subgroup. CRP, C-reactive protein; IHC, immunohistochemistry; LMR, lymphocyte–monocyte ratio; NLR, neutrophil–lymphocyte ratio; TANs, tumor-associated neutrophils; TME, tumor microenvironment; SAA, serum amyloid A; PSCC, penile squamous cell carcinoma.

To investigate the correlation between the preoperative NLR and the level of systemic inflammation, Pearson correlation analysis was performed, which revealed that the NLR was positively corelated with the levels of the inflammatory proteins CRP and SAA but negatively correlated with the LMR (**Figures 2A, B**). In addition, the levels of the proinflammatory cytokines IL-6 and IL-8 were significantly elevated in the high NLR group (n=59), whereas the levels of the anti-inflammatory cytokines IL-4 and IL-10 were significantly reduced (**Figure 2C**). These findings indicate that PC patients with a high NLR have a greater degree of systemic inflammation.

To further explore the tumor inflammatory immune microenvironment in patients grouped according to the NLR. Sixteen tumors were subjected to mRNA-seq to analyze immune cell subtypes via ImmuCellAI (**Supplementary Figure S3** **) (** 25). Although there was no significant difference in the proportion of tumor-infiltrating immune cells (TIICs) between the high-NLR and low-NLR groups, the high-NLR group presented more tumor-associated neutrophils (TANs) and less infiltration of antitumor CD8+ T cells (**Figure 2D**). Moreover, paraffin-embedded tumor samples from 167 tumor tissues were used to verify the findings (**Figures 2E, F**). Notably, a high NLR was significantly correlated with an increased CD66^+^ TAN/CD8+ T-cell ratio (**Figure 2F** **) (** 18). These results suggest that the preoperative NLR in PC patients is associated with a proinflammatory microenvironment in tumors, especially those with an immunosuppressive phenotype.

**Figure 3:**
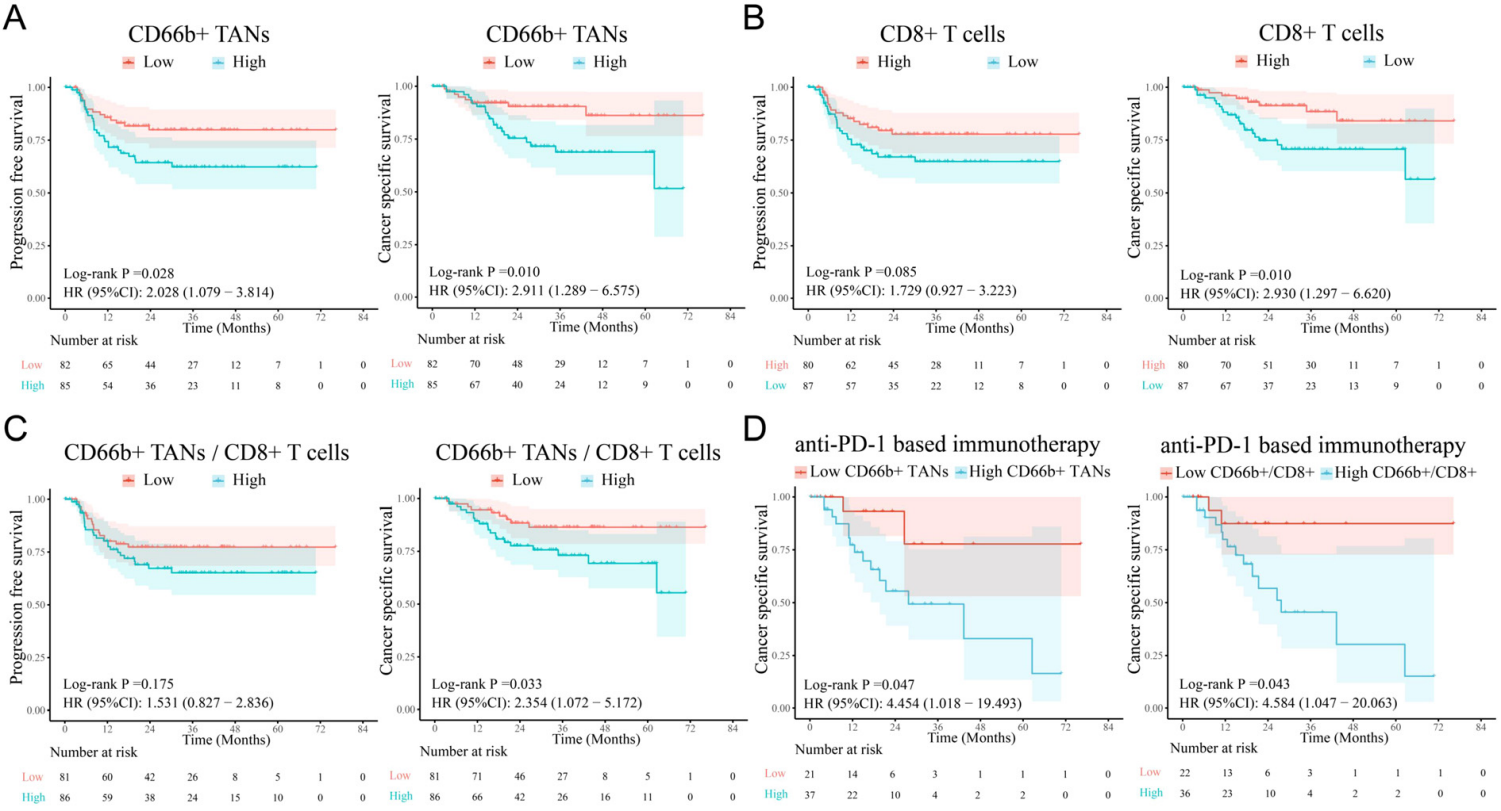
The infiltration levels of TANs and CD8^+^ T cells are correlated with survival in PSCC patients. **(A)** Survival analysis revealed that high TAN infiltration in tumors was associated with poor PFS and CSS in PSCC patients. **(B, C)** A reduction in the number of CD8^+^ T cells and an increase in the CD66b^+^/CD8^+^ T-cell ratio were related to poor survival, although the differences in PFS were not significant. **(D)** Increases in CD66b^+^ TANs and the CD66b^+^/CD8^+^ ratio were associated with poor immunotherapy response. TANs, tumor-associated neutrophils; PFS, progression-free survival; CSS, cancer-specific survival.

More importantly, survival analysis revealed that high infiltration of CD66^+^ TANs was associated with poor PFS and CSS (**Figure 3A** **),** whereas enrichment of CD8+ T cells was correlated with better CSS but was not significantly correlated with PFS (**Figure 3B**). Both the CD66^+^ TAN ratio and the CD66^+^ TAN/CD8+ T-cell ratio indicated the presence of an immunosuppressive phenotype and predicted a poor immunotherapy response and clinical outcomes (**Figures 3C, D**).

### 3.6 The proinflammatory response leads to TAN N2 polarization and NET formation, inducing CD8+ T-cell depletion

**Figure 4:**
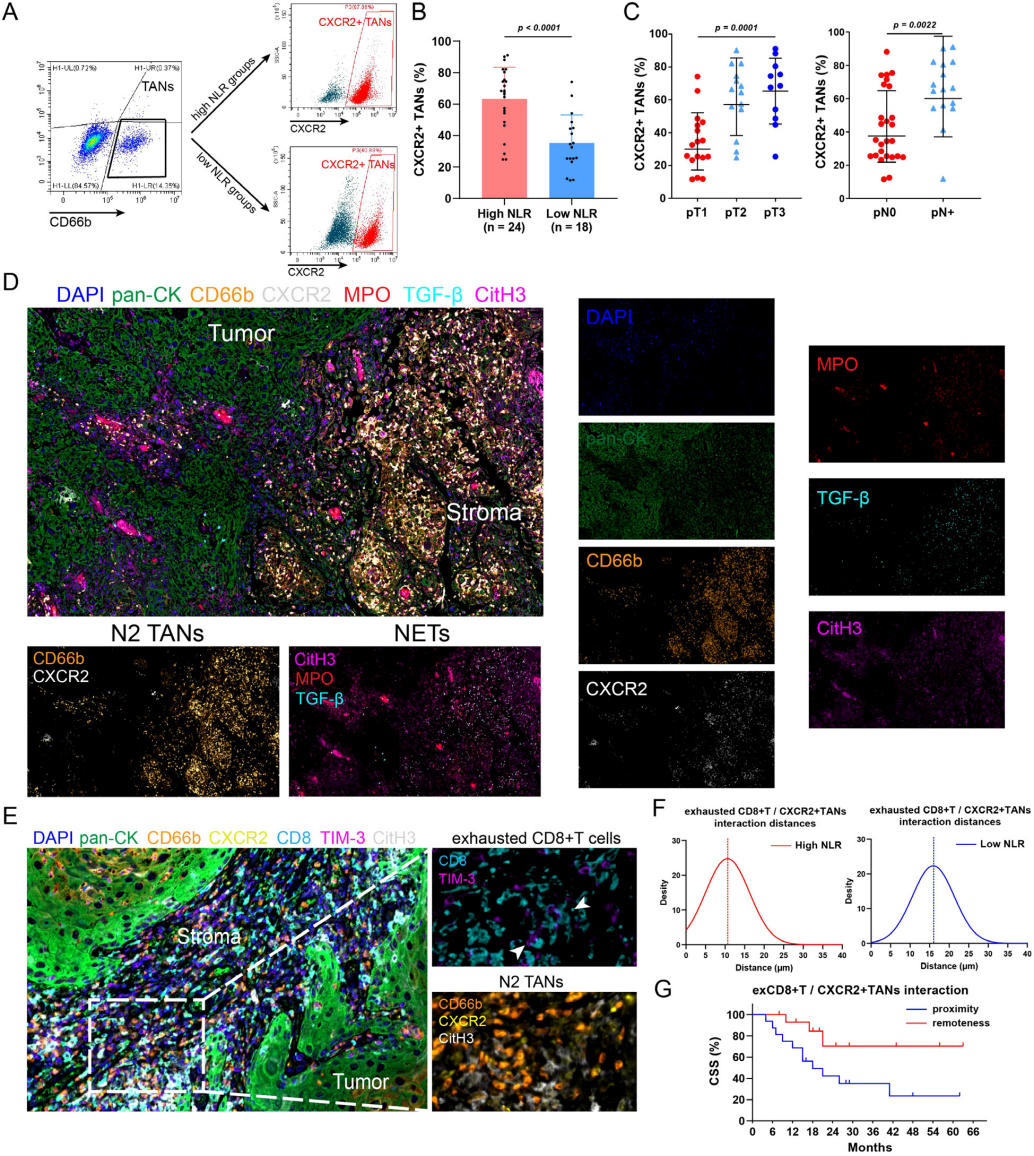
N2 TANs induce NET formation and CD8+ T cell exhaustion in tumors with high NLRs. **(A)** Flow cytometry was conducted to detect N2 TANs (CXCR2^+^CD66^+^) in tumors with different NLRs. **(B)** PSCC patients with high NLRs had increased N2-weighted TAN infiltration in tumors. **(C)** The abundance of N2 TANs in PSCC was associated with advanced pT stage and lymph node metastasis. **(D)** Immunofluorescence staining revealed the tumor and stroma regions in PSCC tumors (400×). In patients with high NLRs, TANs exhibit the N2 phenotype (CXCR2^+^), promote NET formation (marked by CitH3, MPO) and secrete the protumor cytokine TGF-β. **(E)** Multiple immunofluorescence assays revealed the spatial relationship between N2 TANs and exhausted CD8+ T cells (CD8^+^TIM-3^+^) (1000×). **(F)** The relative distance between N2 TANs and exhausted CD8+ T cells was shorter in patients with high NLRs than in those with low NLRs, indicating that NET formation could mediate the dysfunction of CD8+ T cells. **(G)** A close relationship between N2 TANs and exhausted CD8+ T cells was associated with poor survival (12 patients in the proximity group, 15 patients in the remoteness group). TANs, tumor-associated neutrophils; NETs, neutrophil extracellular traps; ex, exhausted; NLR, neutrophil–lymphocyte ratio.

Accumulating evidence indicates that N2 TANs are critical components that stimulate immunosuppression, tumor progression and metastasis and can be induced by the proinflammatory cytokine IL-8 (30, 32–35). To assess the polarization of TANs, flow cytometric analysis was performed; the results revealed that the proportion of N2 TANs (CXCR2^+^CD66^+^) was significantly greater in patients with high NLRs and was associated with advanced pT and pN stages (**Figures 4A, B**). Through mIF staining, we discovered that in tumors from high-NLR patients, N2 TANs formed many NETs through NETosis (36); these NETs were identified via CitH3 and MPO staining and also exhibited high expression of the prometastatic cytokine TGF-β (**Figure 4D**). Previous studies have also indicated that NETs can impair antitumor T-cell responses by increasing CD8+ T-cell exhaustion (37, 38). We found that exhausted CD8^+^ T cells were enriched in high-NLR tumors, which was consistent with N2 TAN infiltration (**Figure 4E**). Spatial distance analysis revealed that exhausted CD8+ T cells were in close proximity to N2 TANs in the high-NLR group (**Figure 4F**). Survival analysis revealed that a closer average distance between these cells was associated with a poorer prognosis (**Figure 4G**). These results suggest that N2 TANs mediate the formation of NETs, which might induce the exhaustion of neighboring CD8+ T cells, thereby promoting the formation of an immunosuppressive microenvironment. This could be the underlying reason for the poor response to immunotherapy and the unfavorable prognosis in patients with high NLRs.

## 4 Discussion

Penile cancer (PC) is more prevalent in underdeveloped regions with poor access to education and medical resources (2). Although PC can be cured in more than 80% of patients with early-stage disease, PC progresses to a life-threatening disease when lymphatic metastasis occurs; up to 40% of newly diagnosed cases in China exhibit lymph node metastasis (1, 39). Definitive TNM staging is the most commonly used method to predict prognosis, but there is a lack of available personalized molecular markers to address intratumor heterogeneity. To enhance therapeutic efficacy, it was great challenges to explore the heterogeneity and complexity of the TME in PC (40). Although previous studies indicate that IDO1, RAB20 and HOXD11 are related to a poor PC prognosis (27, 28, 41); the disadvantages of high cost, high time consumption and poor repeatability for genetic testing of these markers make their clinical application more difficult. The NLR is an economical, simple and widely used inflammatory marker that is related to immunotherapy resistance, regional lymph node metastasis and poor outcomes in patients with PC (16). The preoperative NLR, as an ideal indicator, is beneficial for predicting survival before surgery and can be used to guide treatment and follow-up precisely. However, previous studies of the NLR in PC patients lacked high-quality prognostic evidence, and the relationships between the NLR and the inflammatory TME and the underlying mechanism warrant additional exploration.

In previous studies, it was highly challenging to obtain a large cohort of PC patients with standardized baseline treatment initially. Owing to the surgical timing and boundary of inguinal lymph node dissection remaining controversial in PC (4), the surgical patterns are not consistent. To ensure data quality, all PC patients in our study underwent concurrent lymphadenectomy to obtain clear pN stage information, and prognostic bias attributed to insufficient surgical treatment was ruled out (23). More importantly, most studies did not account for the notably greater proportion of pN+ patients in the high NLR group, which might lead to overestimation of the prognostic value of the NLR. In addition, given the rare nature of this disease, existing studies have focused on small sample sizes and single-center cohorts.

Since 2010, a total of 582 PC patients from four centers were enrolled in our study. The results underscore the significant correlation between a high NLR and adverse clinical characteristics such as pT stage, pN stage, grade, and lymphovascular and perineural invasion. Similar to previous findings, PC patients with high NLRs presented a systemic hyperinflammatory status associated with shorter PFS and CSS (16, 19, 20, 22). Importantly, our study represents the first use of PSM and stratified analysis to control for the prognostic bias of pN stage and revealed that the NLR is also an independent unfavorable prognostic indicator for both PFS (HR: 1.64, 95% CI: 1.15–2.34) and CSS (HR: 1.56, 95% CI: 1.04–2.34). Interestingly, high NLR level patients were also associated with poor chemotherapy and immunotherapy response. Therefore, through rigorous enrollment and reasonable statistical methods, we demonstrated that the preoperative NLR is a simple and reliable marker for PC prognosis evaluation and is particularly suitable for wide use in underdeveloped regions. Besides, preoperative NLR level is an available marker to predict response especially in the neoadjuvant therapy phase, and is also beneficial for postoperative treatment decisions.

Although the NLR reflects the balance between systemic inflammation and adaptive immunity, the dynamic changes in cytokines, secreted proteins and the inflammatory TME in PC remain unclear. Wang et al. reported that IL1RN and PRRX1 is the prognostic biomarker correlated with immune infiltrates in colorectal cancer (42). The increase in the NLR was consistent with the trends in the levels of CRP and the proinflammatory cytokines IL-6, IL-2 and TNF-α (43). Similarly, we found that a high NLR was associated with high SAA, CRP and serum IL-6 and IL-8 levels in PC patients, indicating a direct link between the NLR and the inflammatory immune microenvironment.

To further characterize the inflammatory microenvironment in high-NLR patients, comprehensive sequencing, IHC and mIF were performed, and the results revealed an immunosuppressive phenotype due to the enrichment of TANs and decreased enrichment of CD8+ T cells. It was reported that IL-6 and IL-8 recruit and induce the migration of macrophages and neutrophils into the TME to amplify inflammatory signaling and promote malignant progression (44). In addition, IL-8 plays a dominant role in the polarization of TANs toward N2, which is widely associated with immunosuppression and metastasis (32, 33, 44–46). Our results proved that the preoperative NLR is strongly related to the N2-weighted TAN/CD8+ T-cell ratio in the TME. In PC patients, a high-NLR was related to increased IL-8 secretion, TAN enrichment, N2 phenotype differentiation, reduced CD8+ T-cell infiltration. The imbalance in the TAN/CD8+ T-cell ratio is a key feature that explains the poor prognosis and immunotherapy resistance of high-NLR patients. On the other hand, N2 TANs can induce NET formation by forming web-like DNA structures and releasing reactive oxygen species, cytokines and granular proteins to promote tumor dissemination and metastasis (47, 48). NETs facilitate tumor progression and poor outcomes through various mechanisms. For example, the release of matrix metalloproteinase-9 and neutrophil elastase promotes degradation of the extracellular matrix, creating a more permissive environment for tumor cell invasion (49). Second, NETs induce TGF-β and VEGF secretion to increase tumor angiogenesis, which supplies nutrients and oxygen, further supporting their growth and metastatic potential (50). Moreover, NETs mediate epithelial–mesenchymal transition by continuously secreting protumor cytokines to promote the acquisition of mesenchymal and invasive properties by tumor cells (51). We found that many NETs formed around N2 TANs in PC tissues with high NLRs. We also found that oversecretion of TGF-β by N2 TANs induced CD8+ T-cell depletion and might facilitate metastasis by inducing angiogenesis. Our results preliminarily revealed that in PC patients with high NLRs, the proinflammatory immune microenvironment might promote tumor progression and was associated with poor prognosis. Further understanding of the detailed mechanisms is urgently needed to improve current treatments and outcomes.

Finally, the study has several limitations. First, it was a retrospective observational study with inherent bias and limited by the small sample sizes of patients performing with mRNA-seq. The clinical significance of the NLR needs to be validated in other centers, especially in cohorts from different areas with different races. Second, a prospective study design is essential to validate the predictive role of the NLR in guiding treatment decision making and follow-up strategies. Besides, PSM methods excluded substantial portions of the whole cohort, potentially distorting the true effects of covariates. Although we highlight the relationship between the NLR and the suppressive immune microenvironment, the associated molecular pathways and interventions need to be further explored.

## 5 Conclusion

In summary, we used PSM to demonstrate that the NLR is an independent unfavorable prognostic indicator in a high-quality and large multicenter cohort of PC patients. A high preoperative NLR is associated with a systematic inflammatory response that suppresses the immune response by promoting N2 TAN infiltration, NET formation and CD8+ T-cell exhaustion and ultimately mediates immunotherapy resistance and poor outcomes.

## Data Availability

The raw data supporting the conclusions of this article will be made available by the authors, without undue reservation.
