# Supplementary figures and images for: Neutrophil-to-lymphocyte ratio predicts a poor prognosis for penile cancer with an immunosuppressive tumor microenvironment
Source: Front Immunol. 2025 Apr 16;16:1568825. doi: 10.3389/fimmu.2025.1568825 (PMC12041217; doi:10.3389/fimmu.2025.1568825)

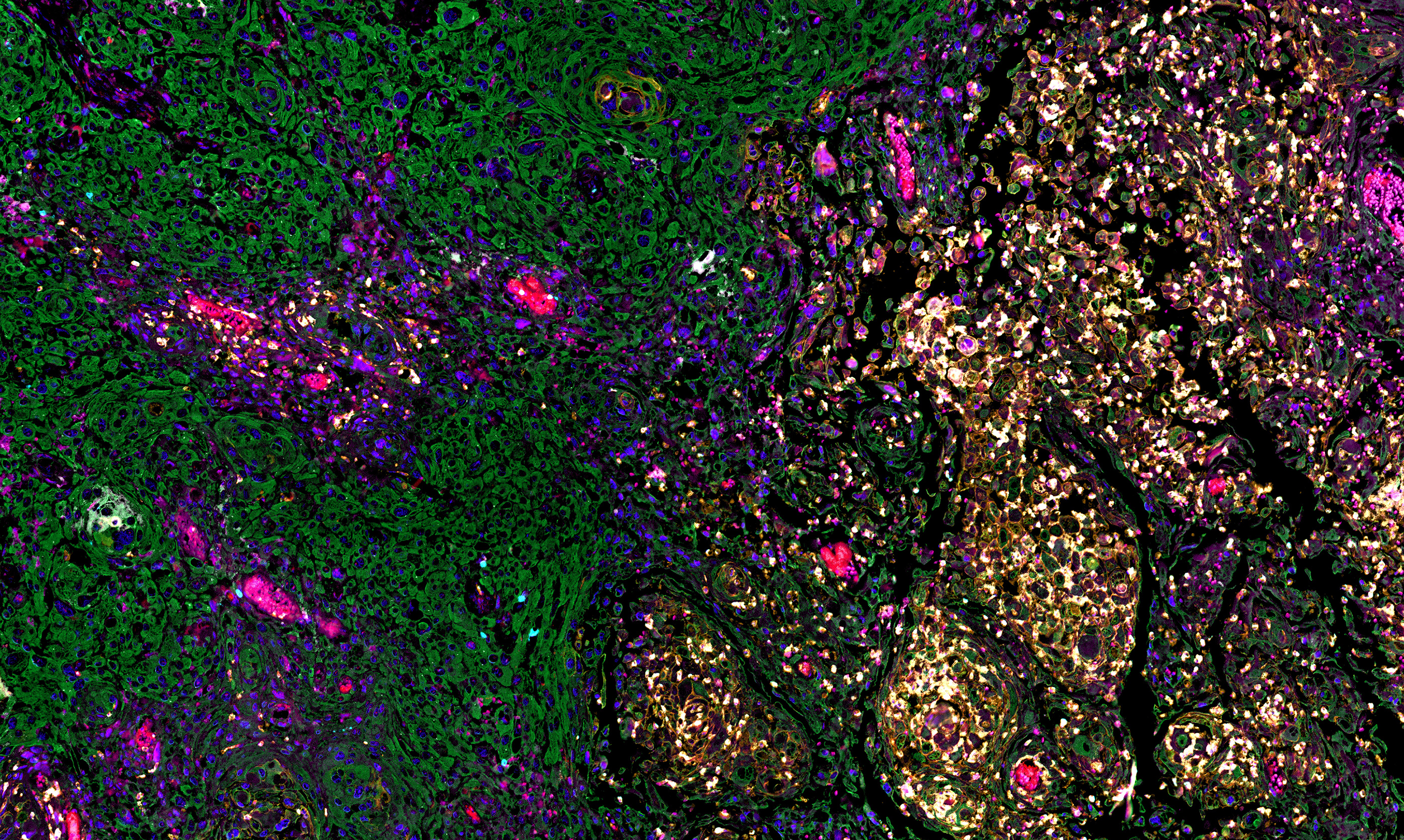

Supplement: Supplementary file 1 [file Image1.jpeg]

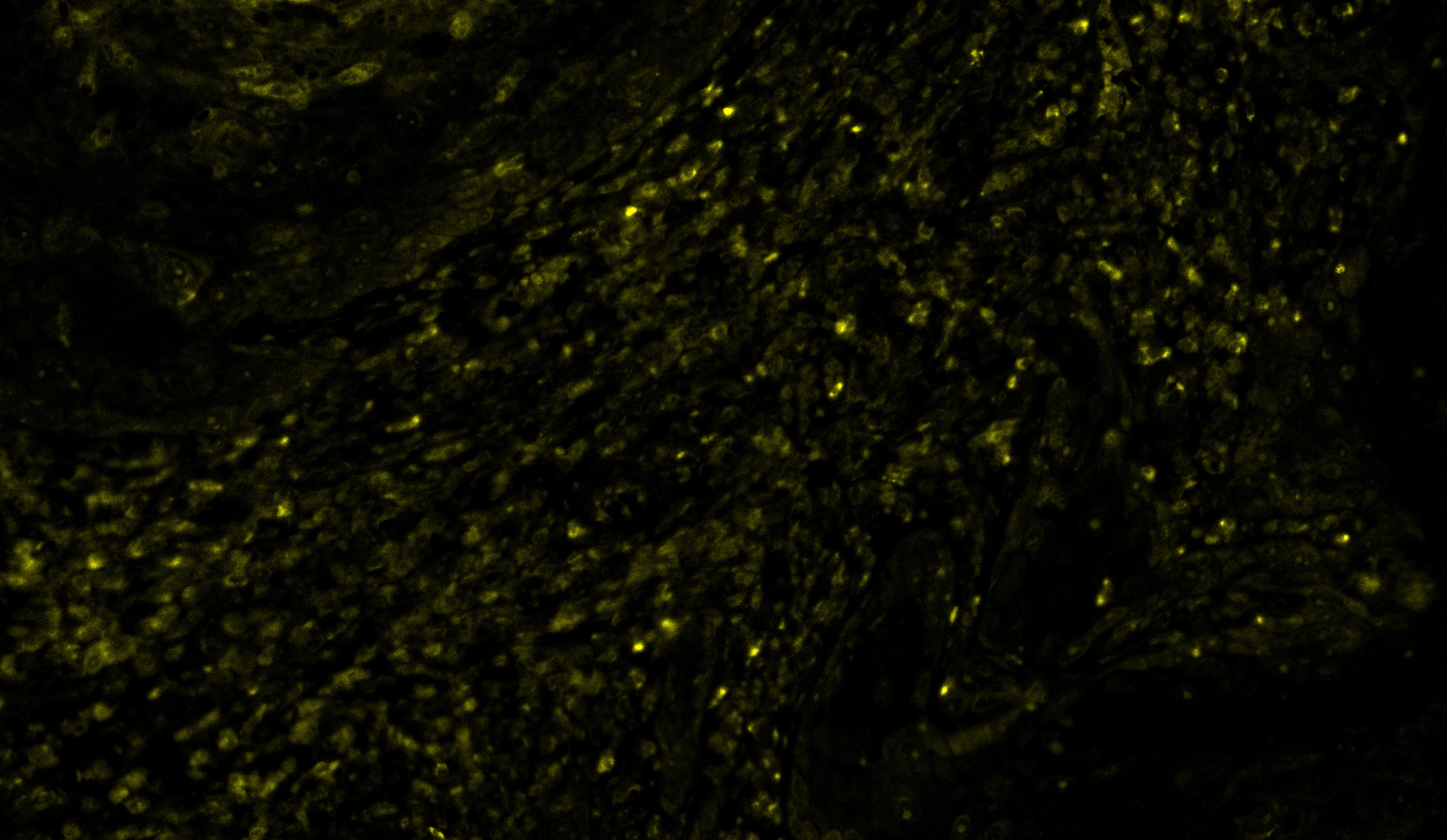

Supplement: Supplementary file 3 [file DataSheet2.zip › CD8ExhaustedT/CD8 exhaustedT/CXCR2.jpg]

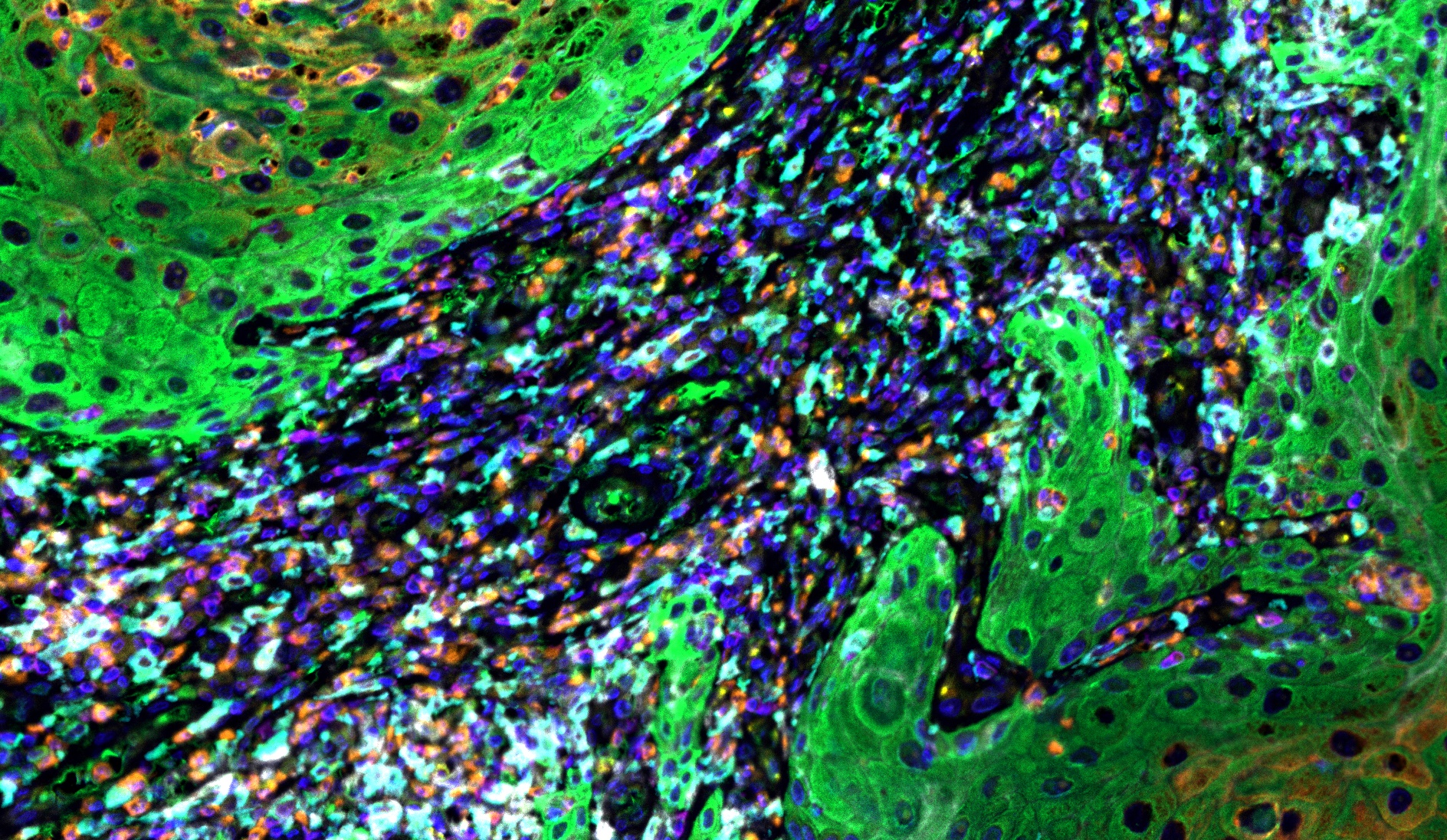

Supplement: Supplementary file 3 [file DataSheet2.zip › CD8ExhaustedT/CD8 exhaustedT/Merge.jpg]

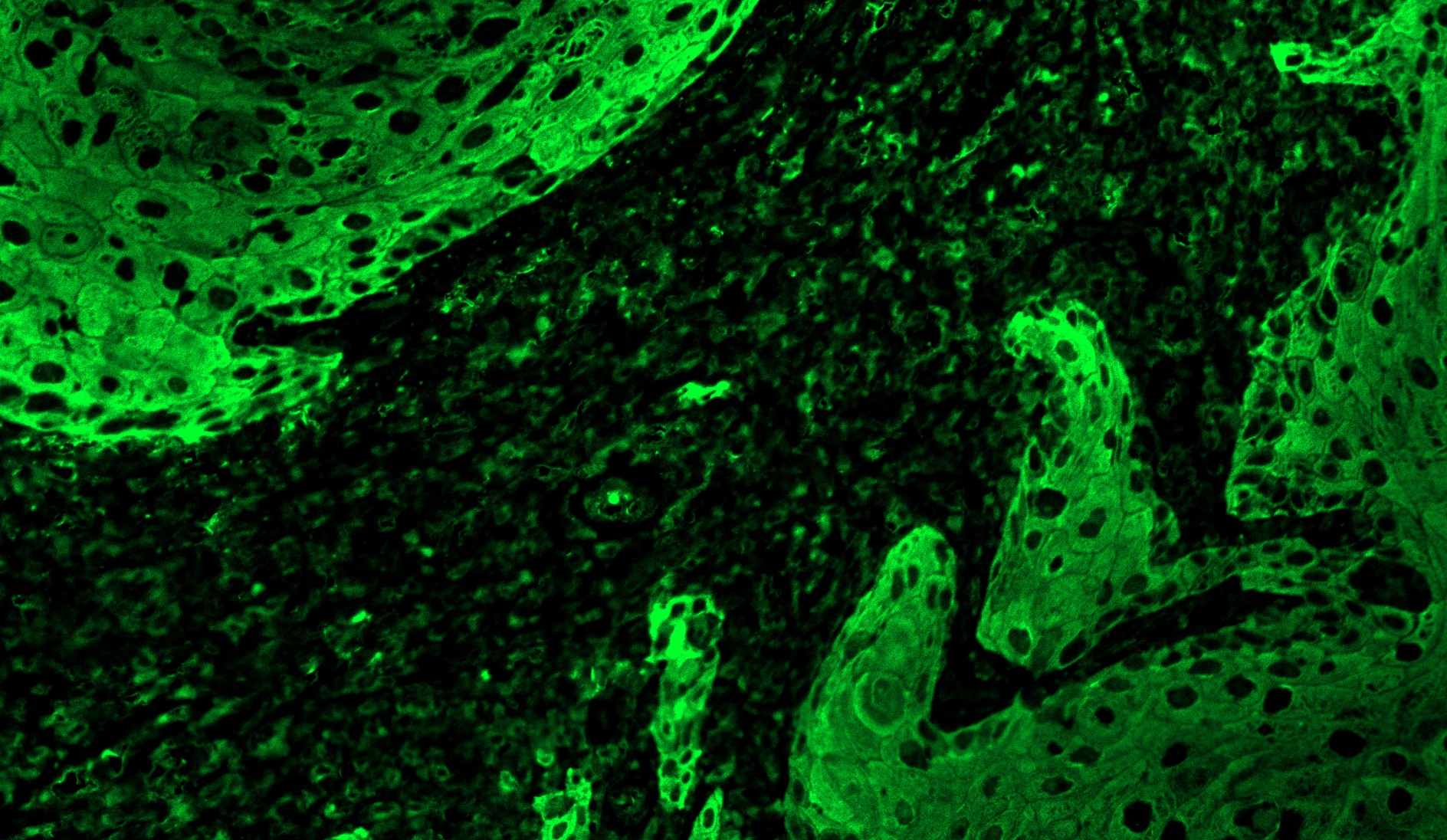

Supplement: Supplementary file 3 [file DataSheet2.zip › CD8ExhaustedT/CD8 exhaustedT/PAN-CK.jpg]

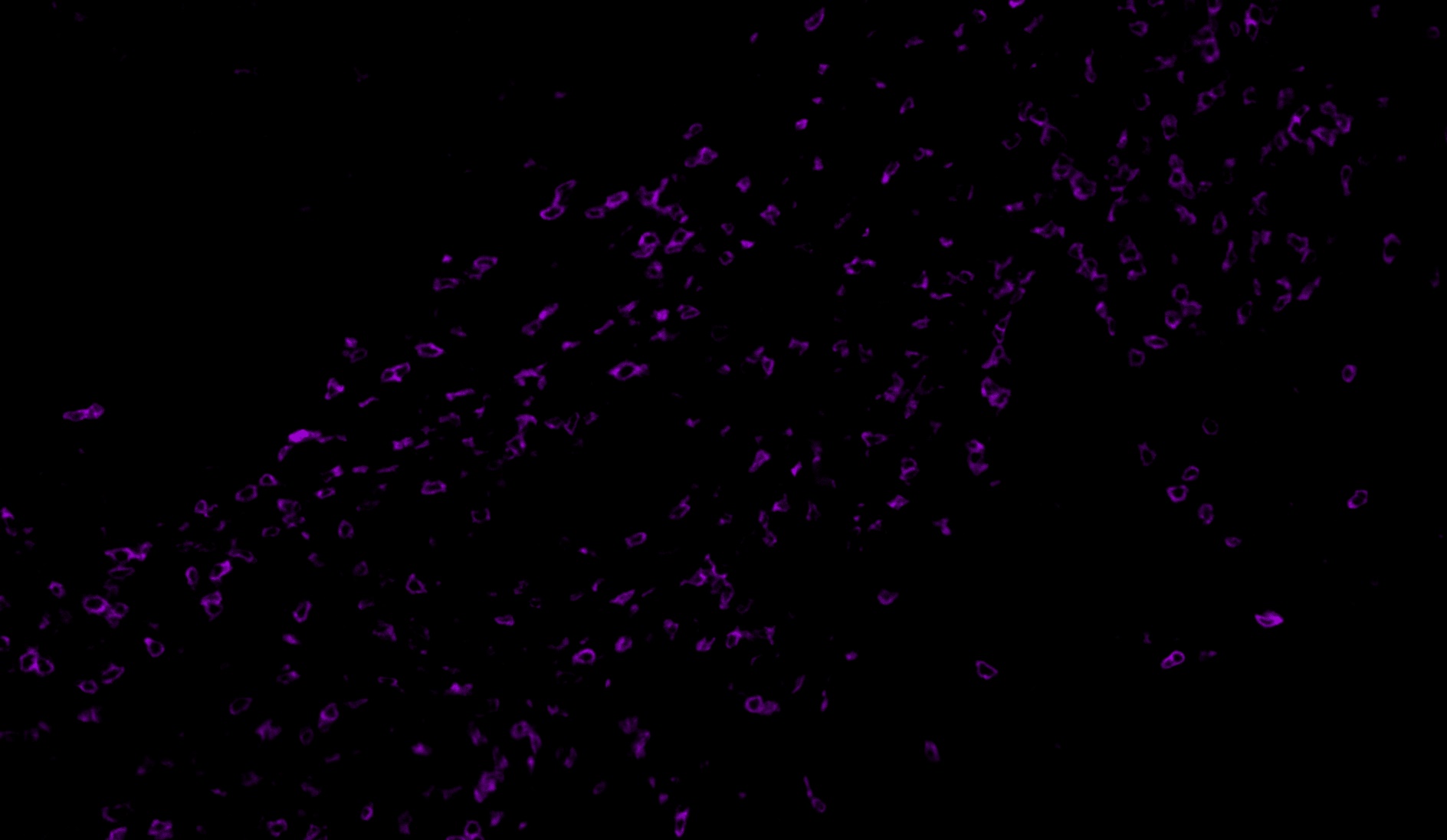

Supplement: Supplementary file 3 [file DataSheet2.zip › CD8ExhaustedT/CD8 exhaustedT/TIM-3.jpg]

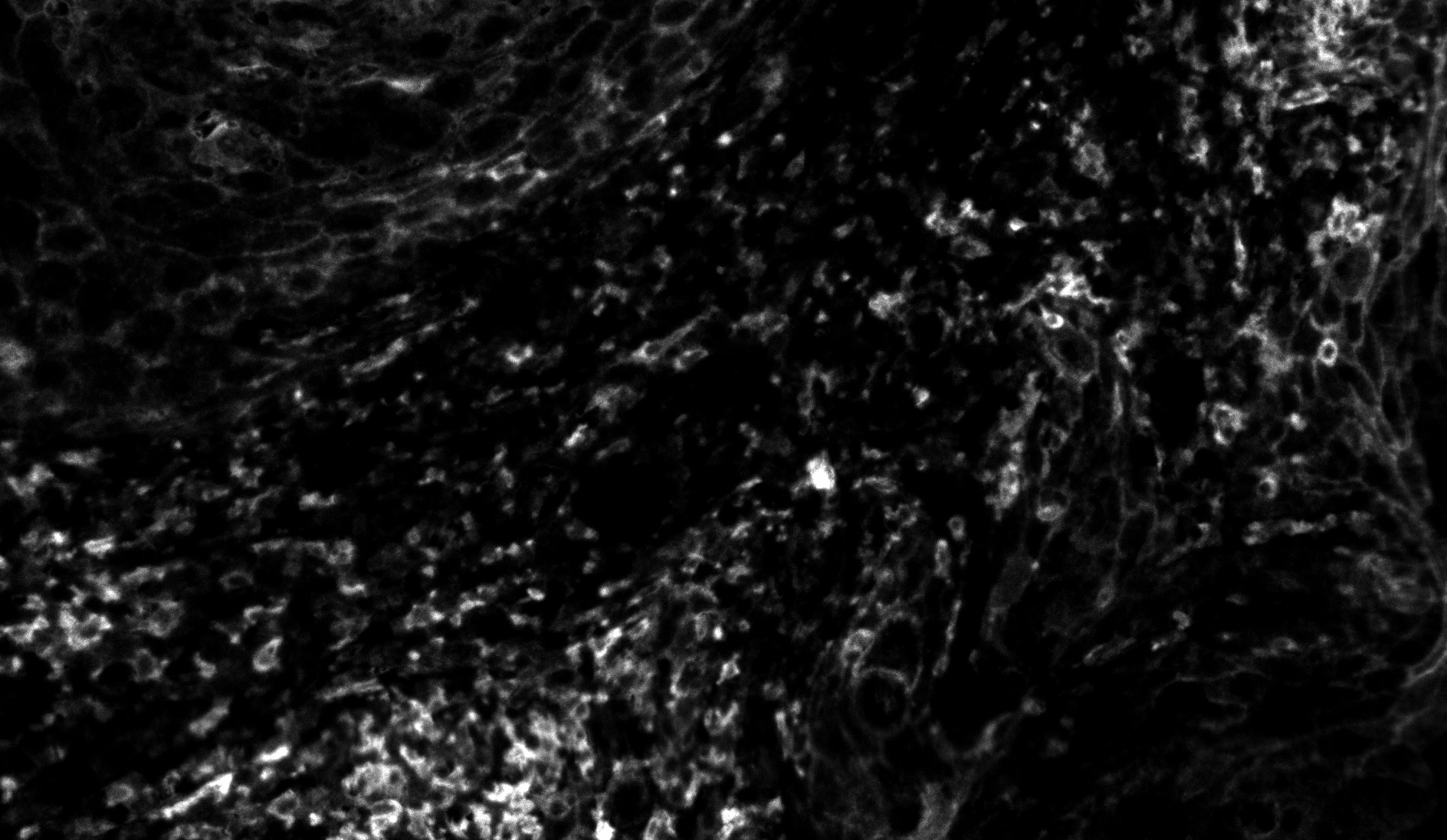

Supplement: Supplementary file 3 [file DataSheet2.zip › CD8ExhaustedT/CD8 exhaustedT/citH3.jpg]

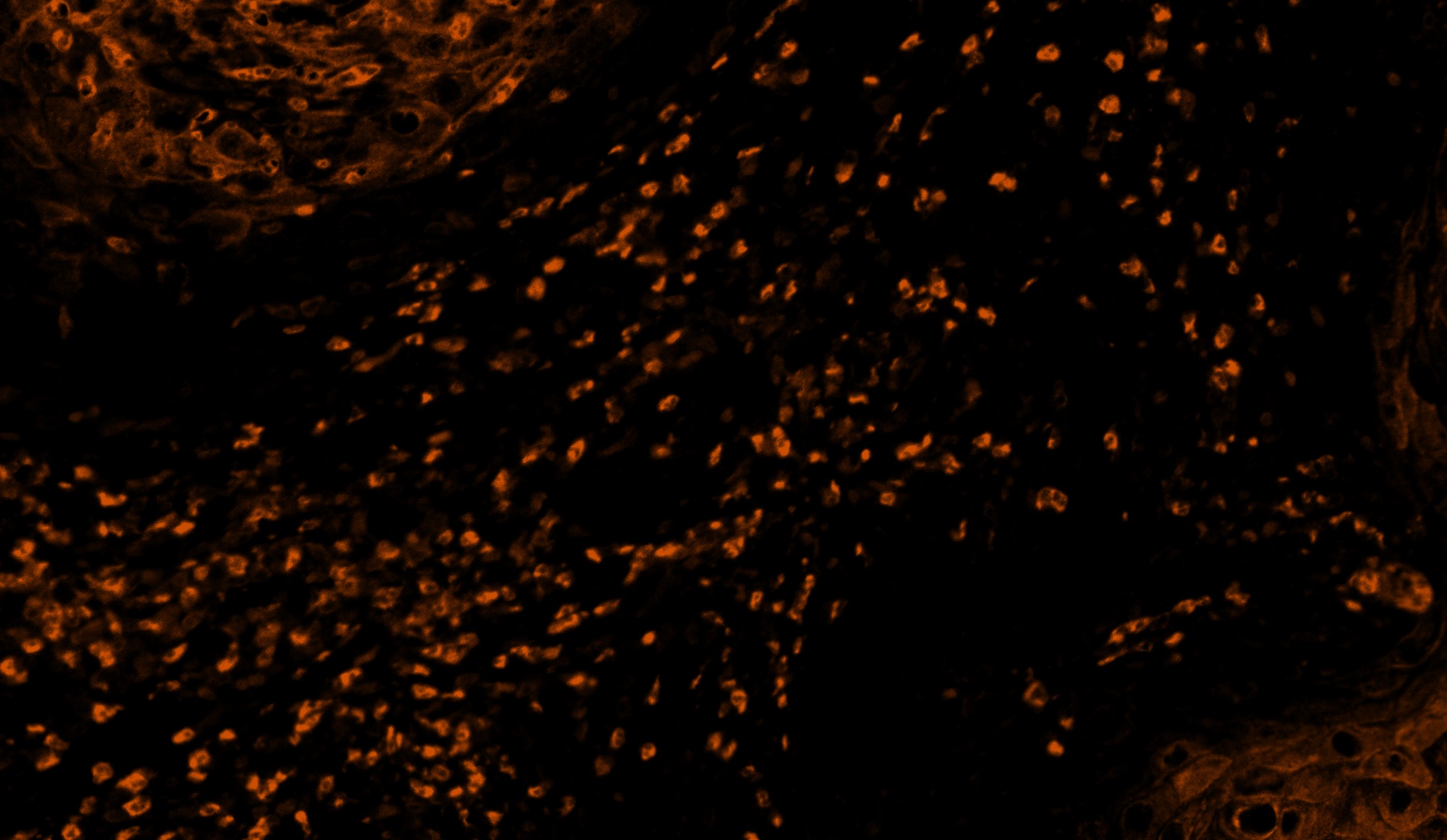

Supplement: Supplementary file 3 [file DataSheet2.zip › CD8ExhaustedT/CD8 exhaustedT/CD66b.jpg]

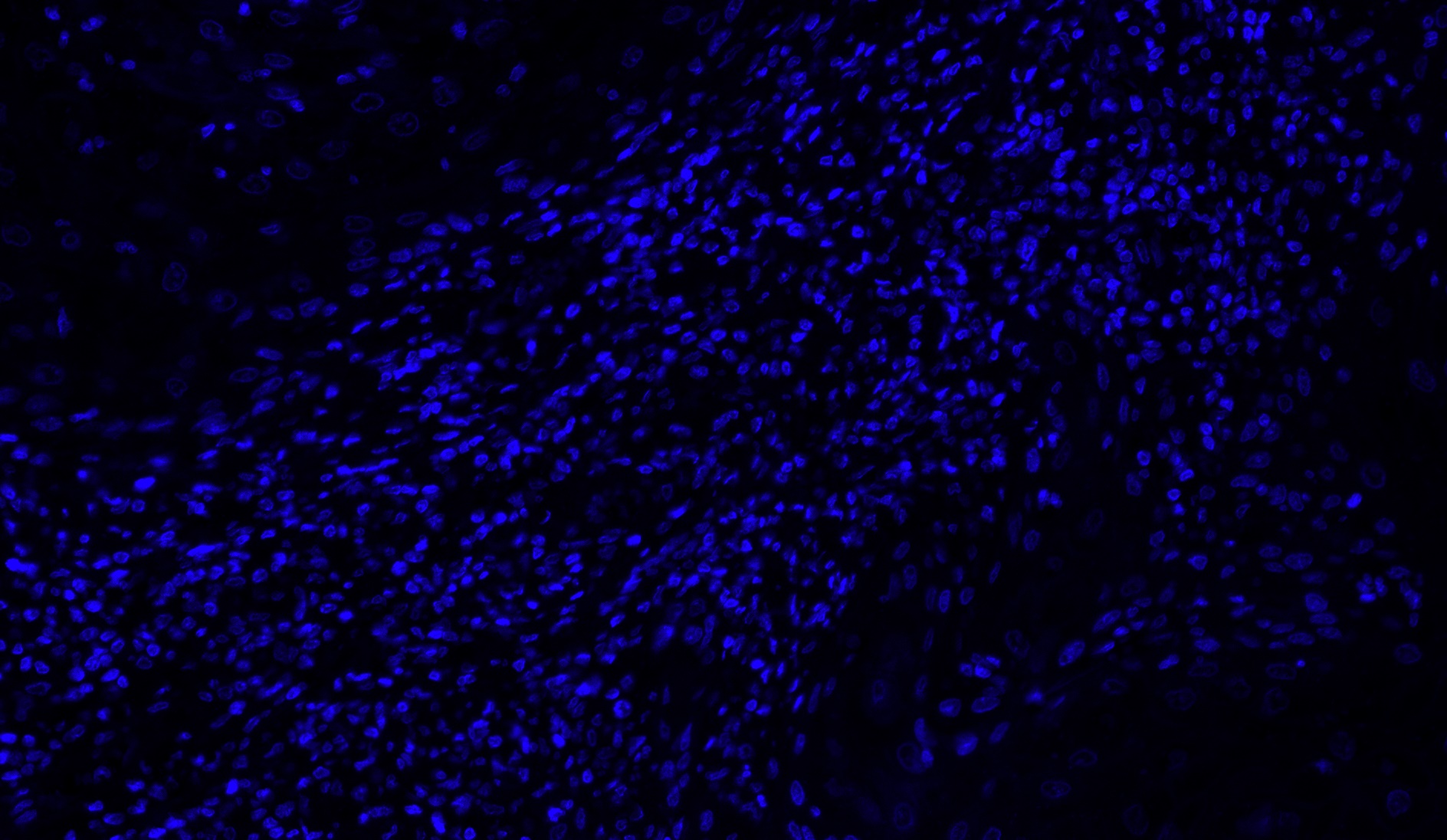

Supplement: Supplementary file 3 [file DataSheet2.zip › CD8ExhaustedT/CD8 exhaustedT/DAPI.jpg]

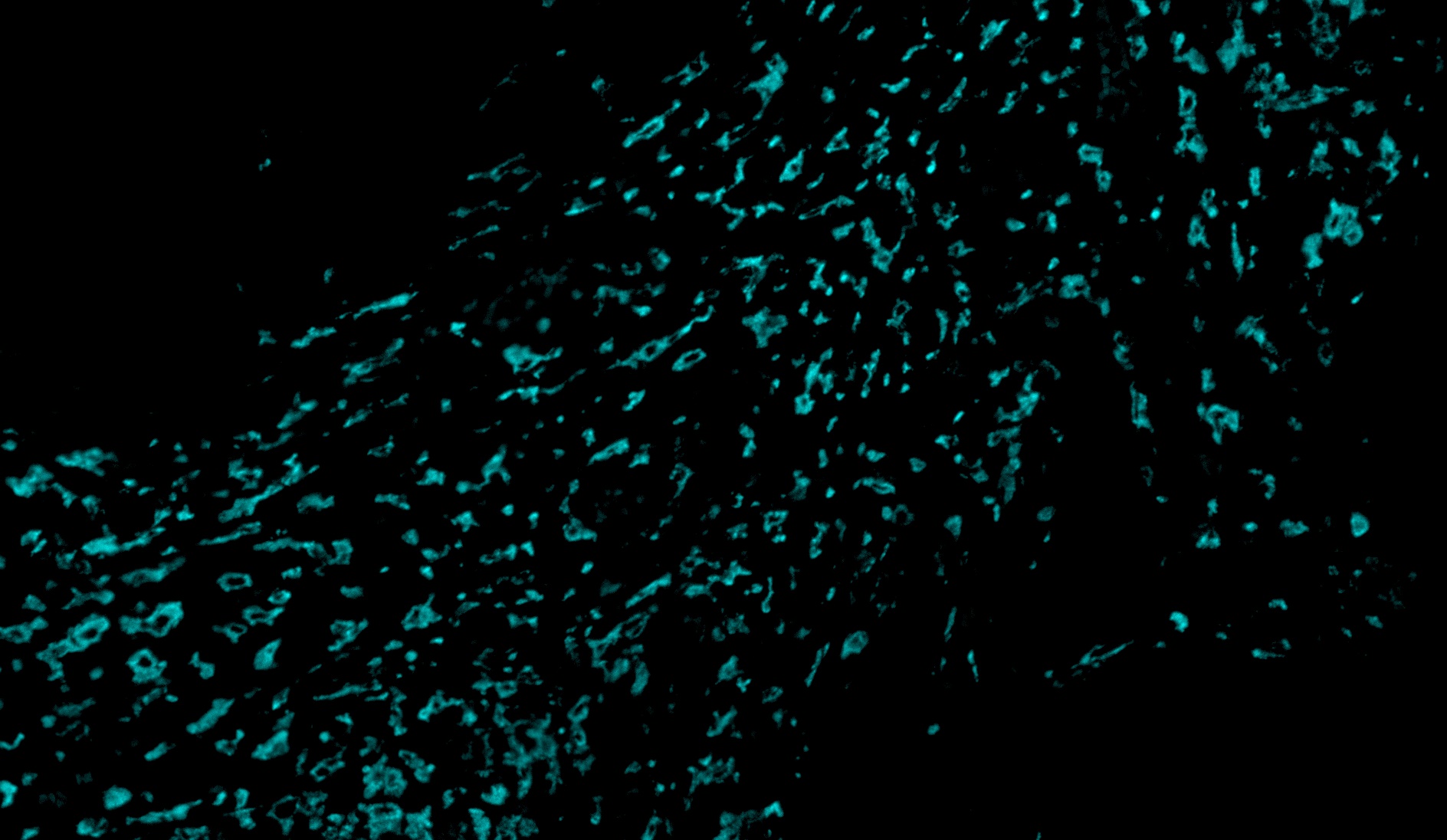

Supplement: Supplementary file 3 [file DataSheet2.zip › CD8ExhaustedT/CD8 exhaustedT/CD8 t.jpg]

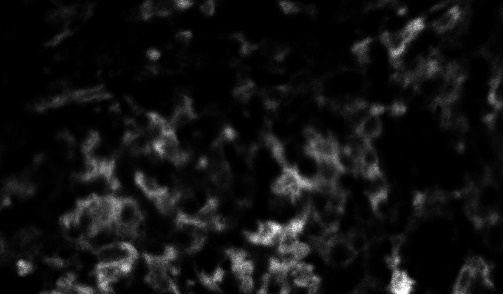

Supplement: Supplementary file 3 [file DataSheet2.zip › CD8ExhaustedT/Zoom in/Untitled26_Nets.jpg]

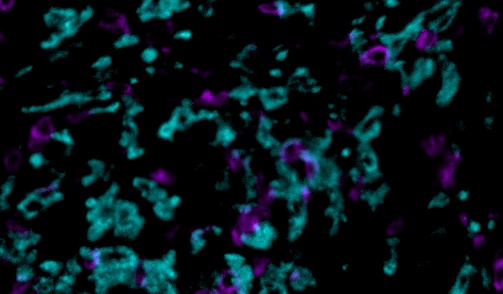

Supplement: Supplementary file 3 [file DataSheet2.zip › CD8ExhaustedT/Zoom in/Untitled26_exhust-CD8T.jpg]

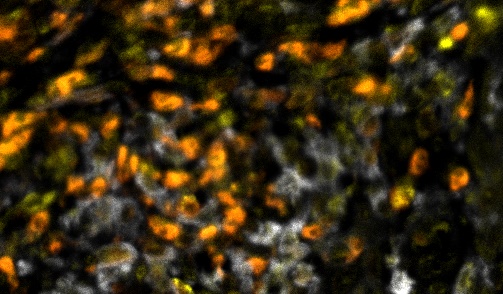

Supplement: Supplementary file 3 [file DataSheet2.zip › CD8ExhaustedT/Zoom in/Untitled26_TANs,NETs.jpg]

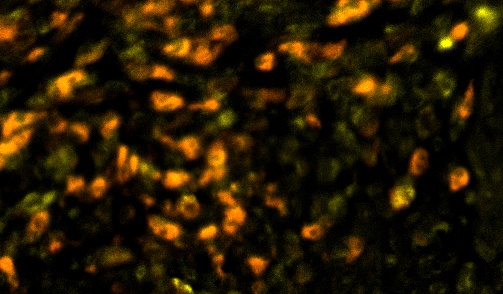

Supplement: Supplementary file 3 [file DataSheet2.zip › CD8ExhaustedT/Zoom in/Untitled26_TANs.jpg]

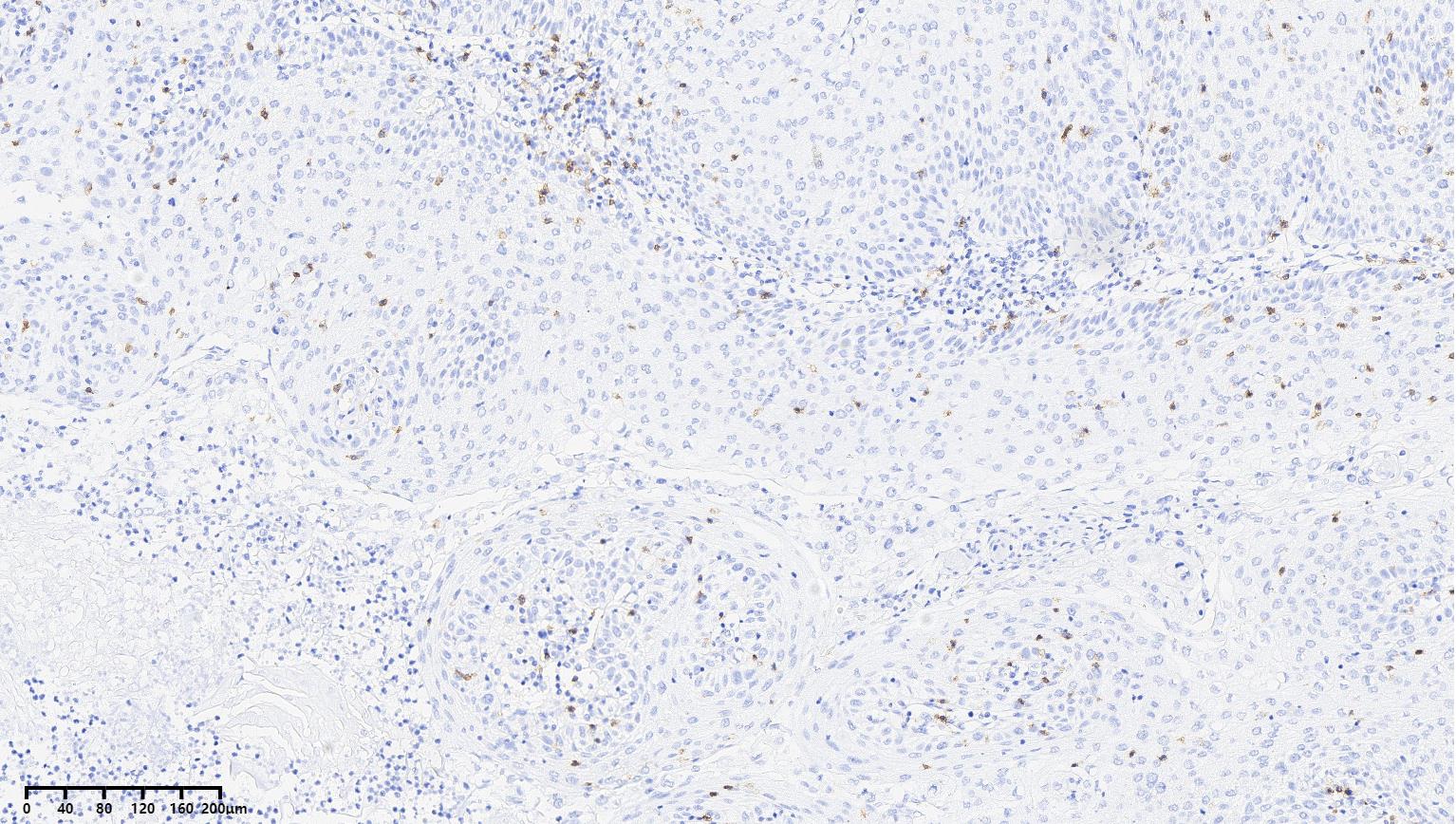

Supplement: Supplementary file 4 [file DataSheet3.zip › high NLR/817746--CD8_10.00X.jpg]

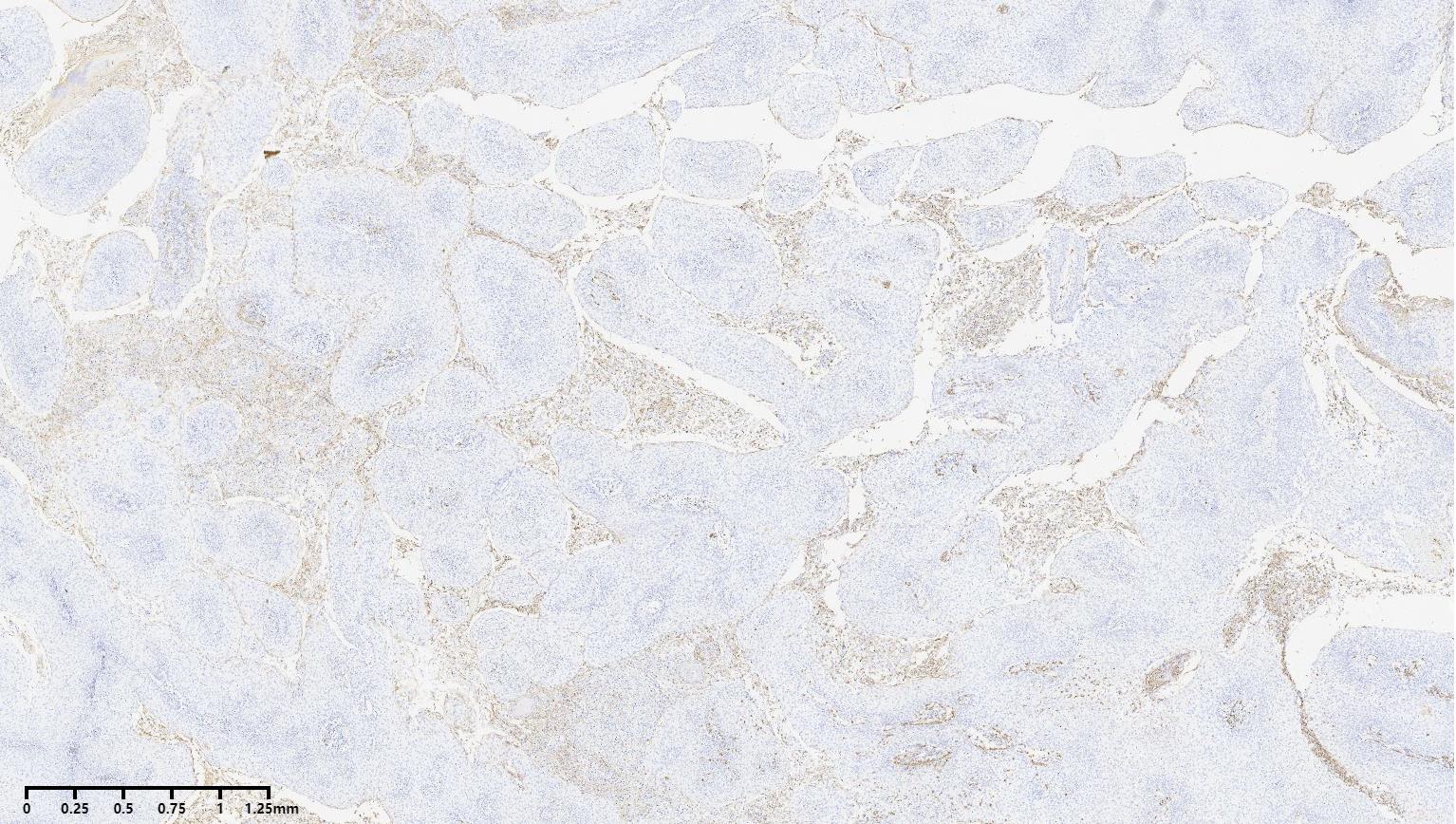

Supplement: Supplementary file 4 [file DataSheet3.zip › high NLR/817746---CD66b_2.00X.jpg]

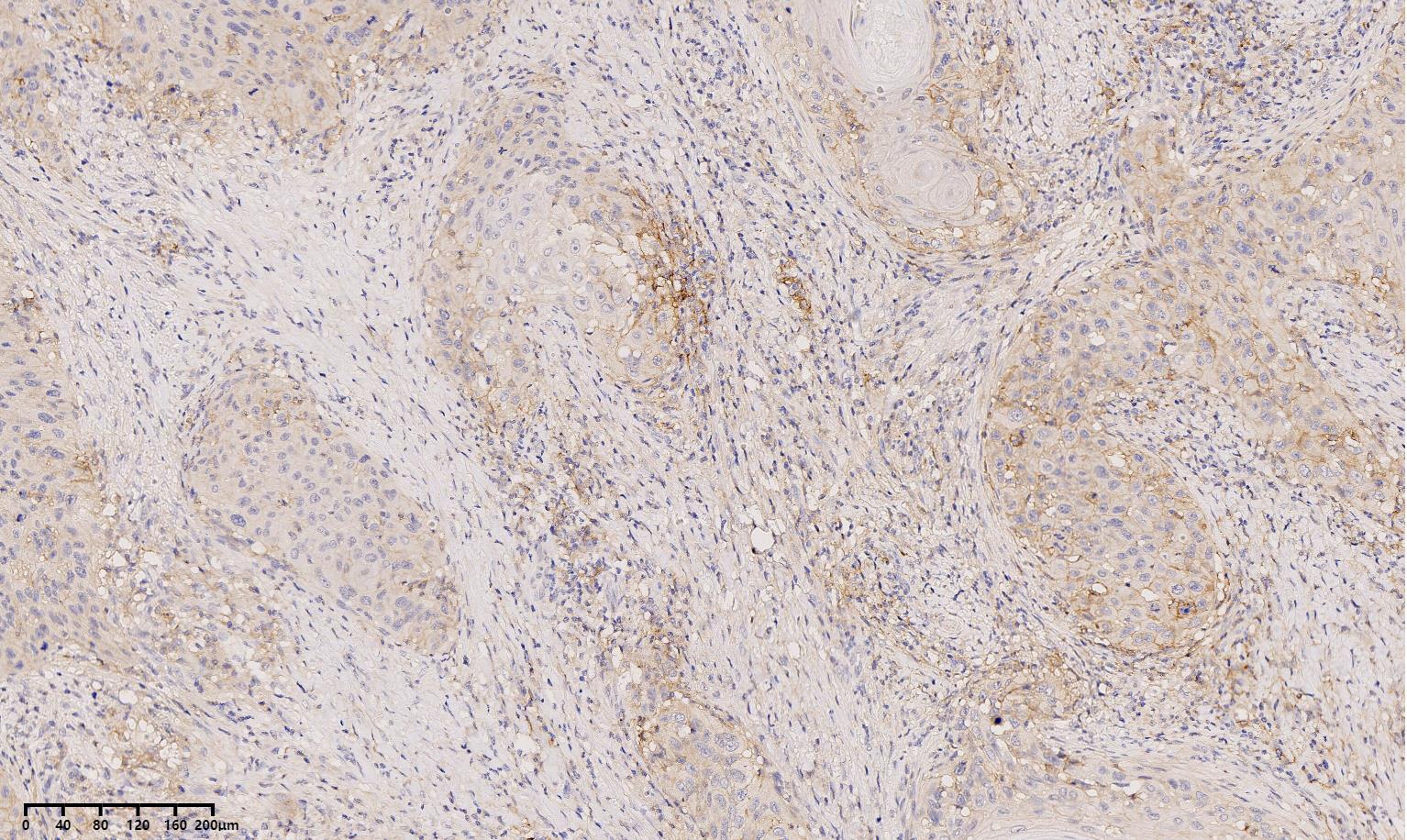

Supplement: Supplementary file 4 [file DataSheet3.zip › high NLR/MMP9_10.00X.jpg]

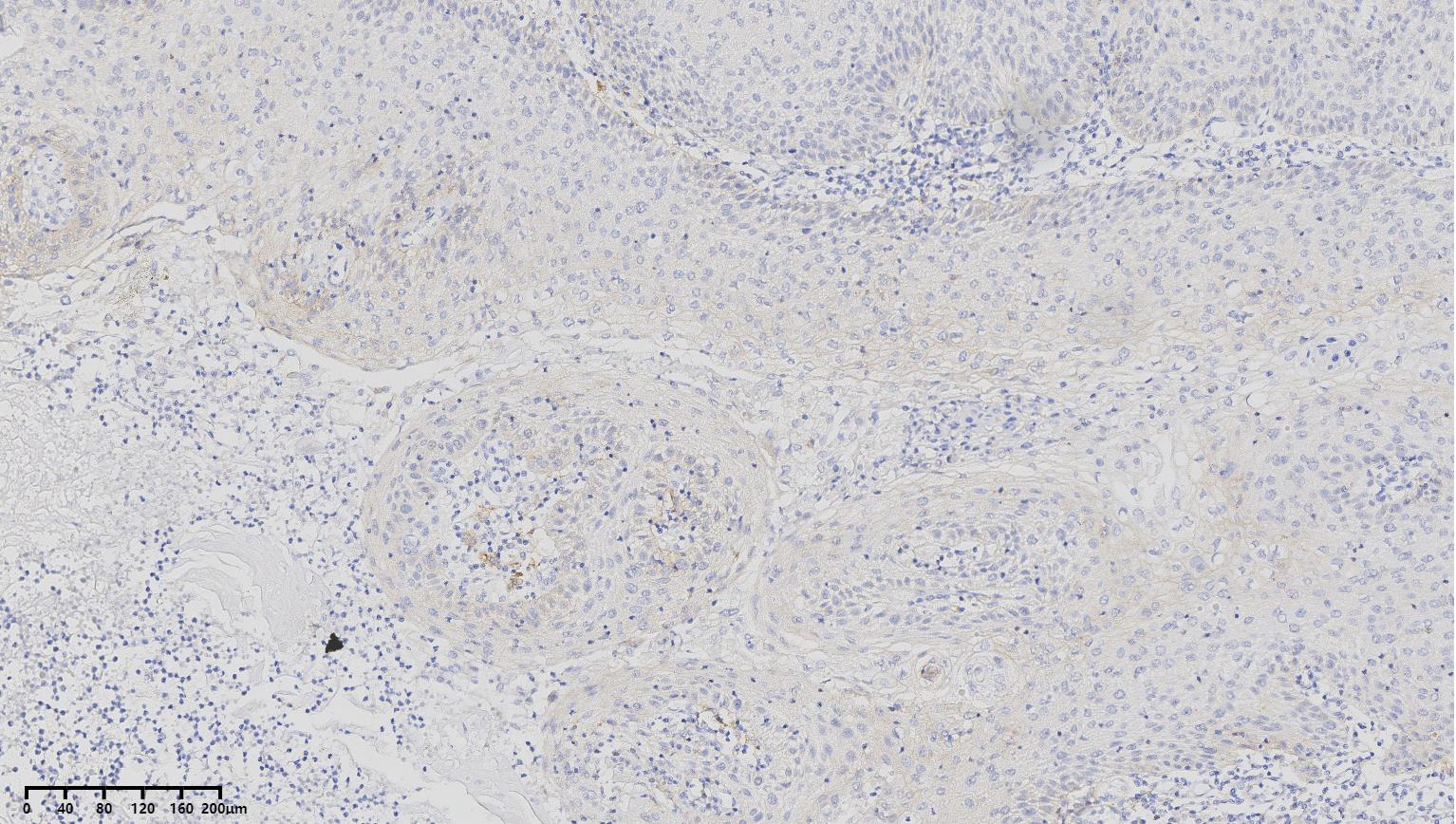

Supplement: Supplementary file 4 [file DataSheet3.zip › high NLR/817746--PDL1_10.00X.jpg]

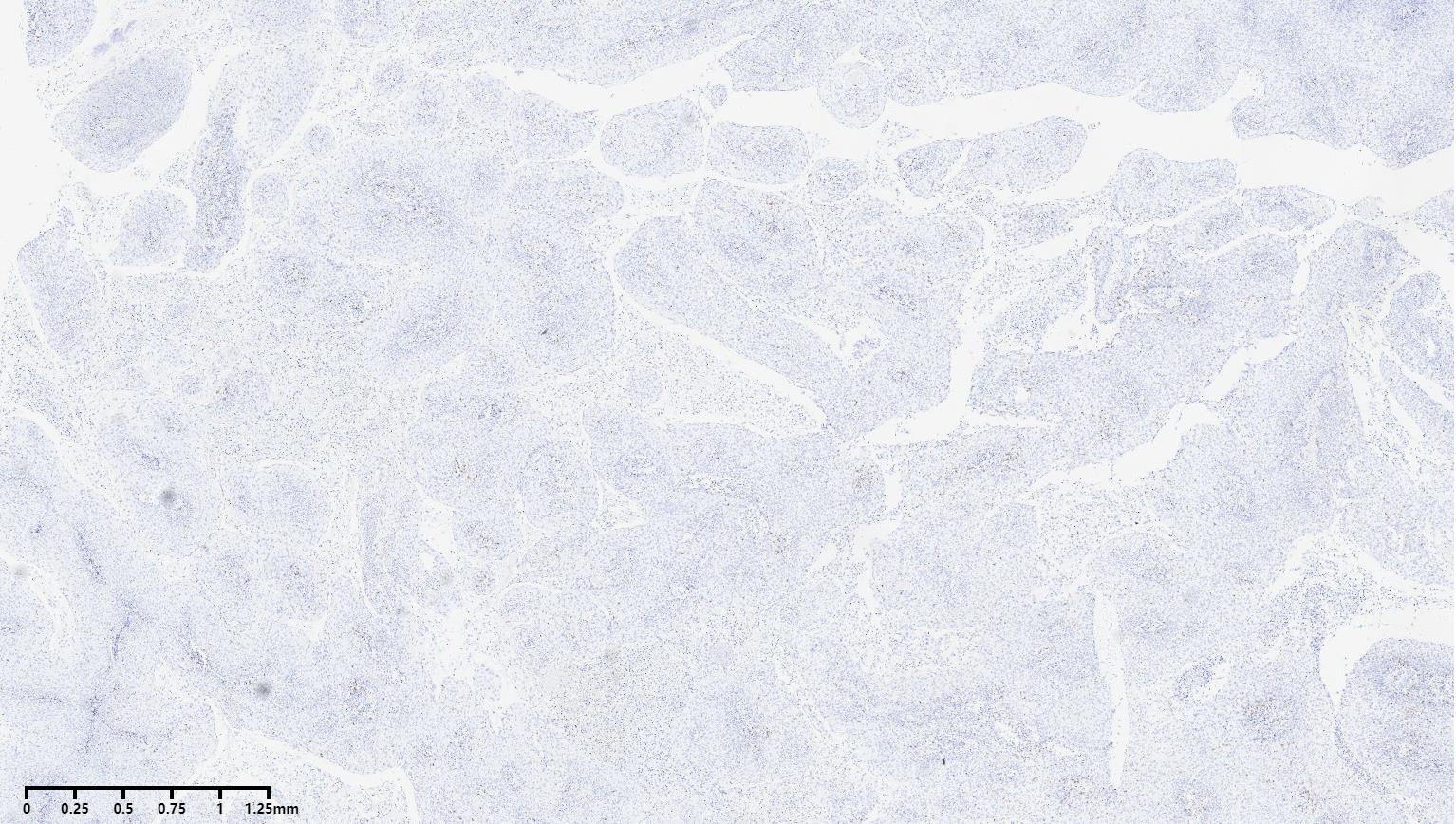

Supplement: Supplementary file 4 [file DataSheet3.zip › high NLR/817746--CD8_2.00X.jpg]

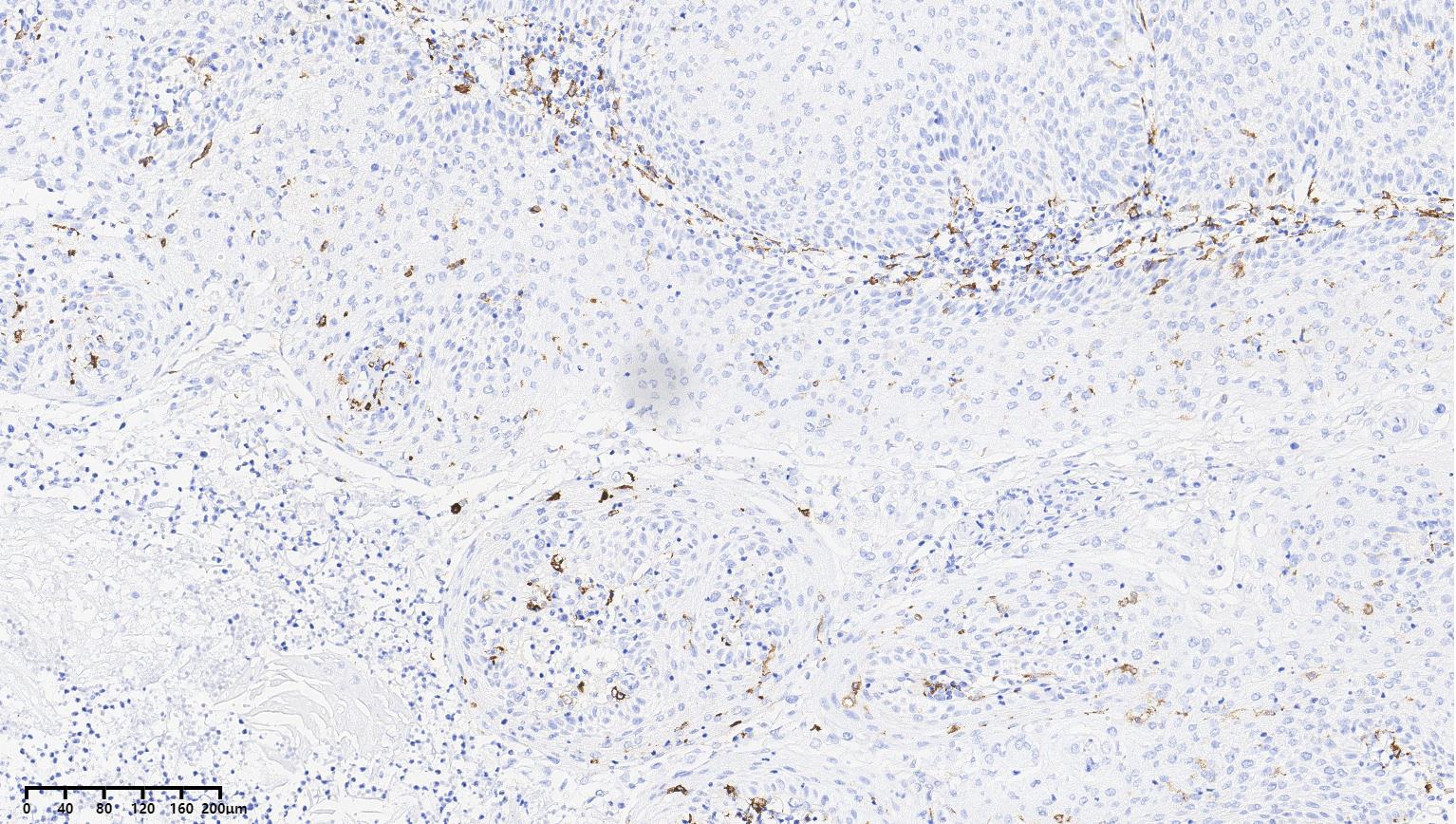

Supplement: Supplementary file 4 [file DataSheet3.zip › high NLR/817746---CD4_10.00X.jpg]

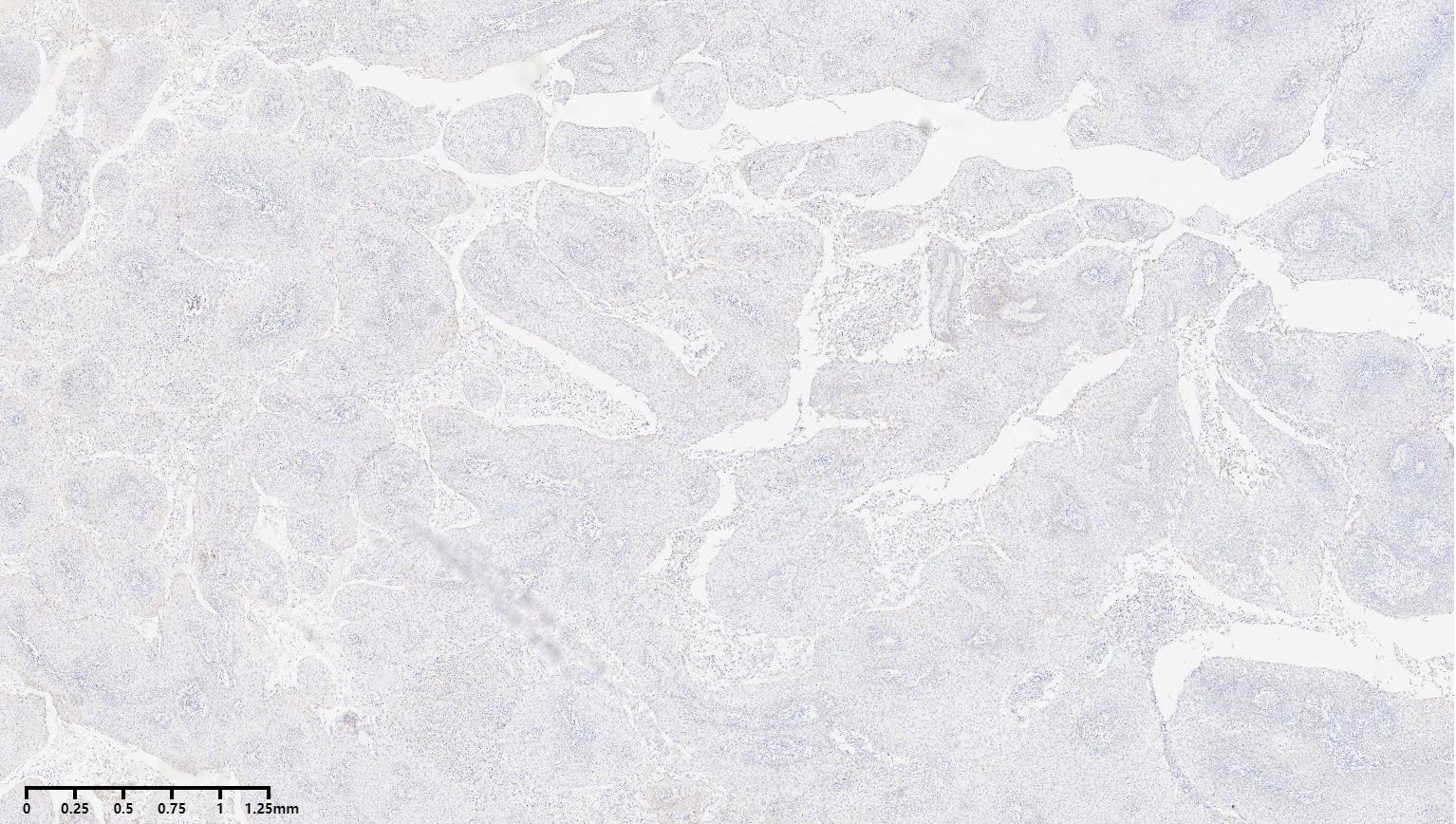

Supplement: Supplementary file 4 [file DataSheet3.zip › high NLR/817746--PDL1_2.00X.jpg]

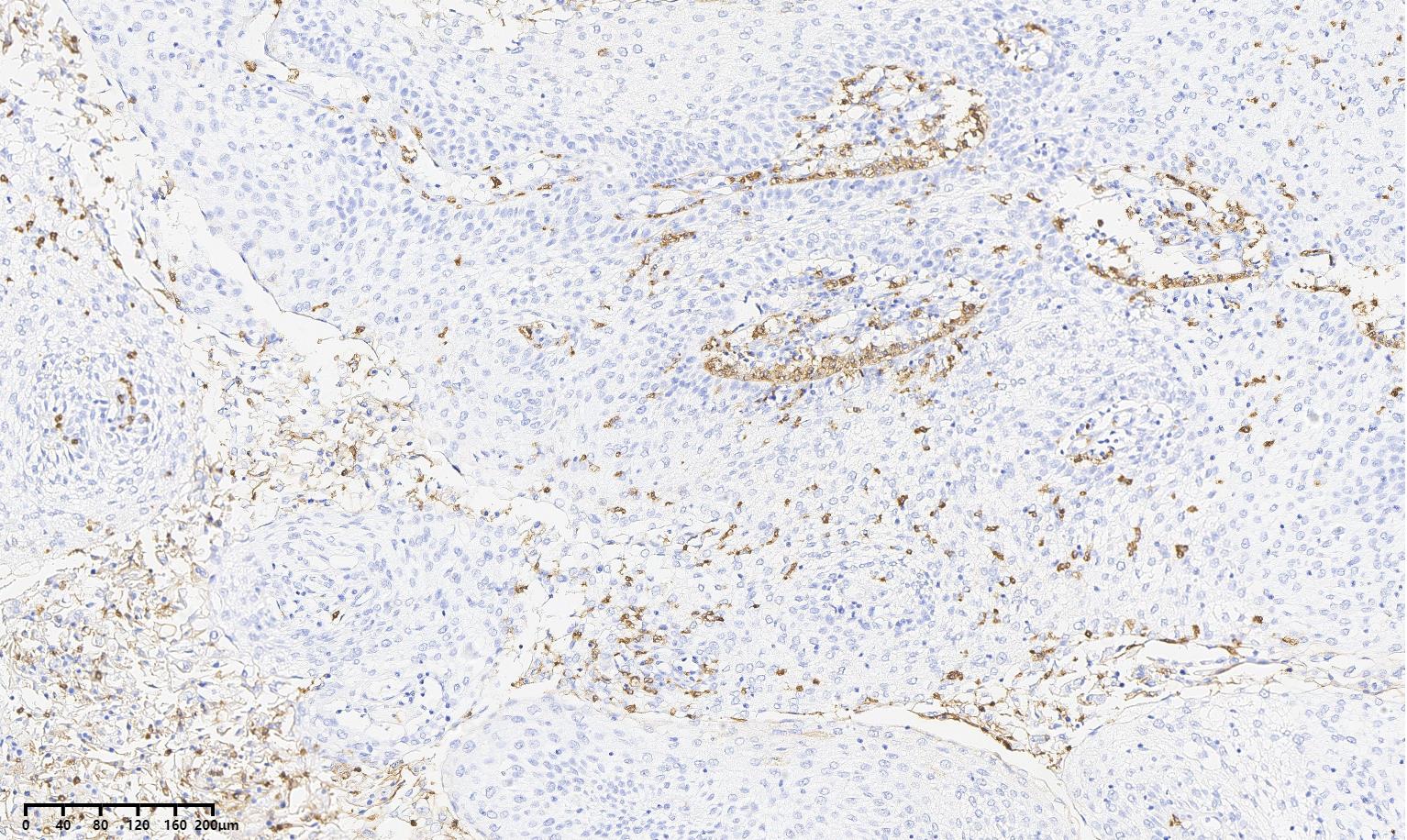

Supplement: Supplementary file 4 [file DataSheet3.zip › high NLR/817746---CD66b_10.00X.jpg]

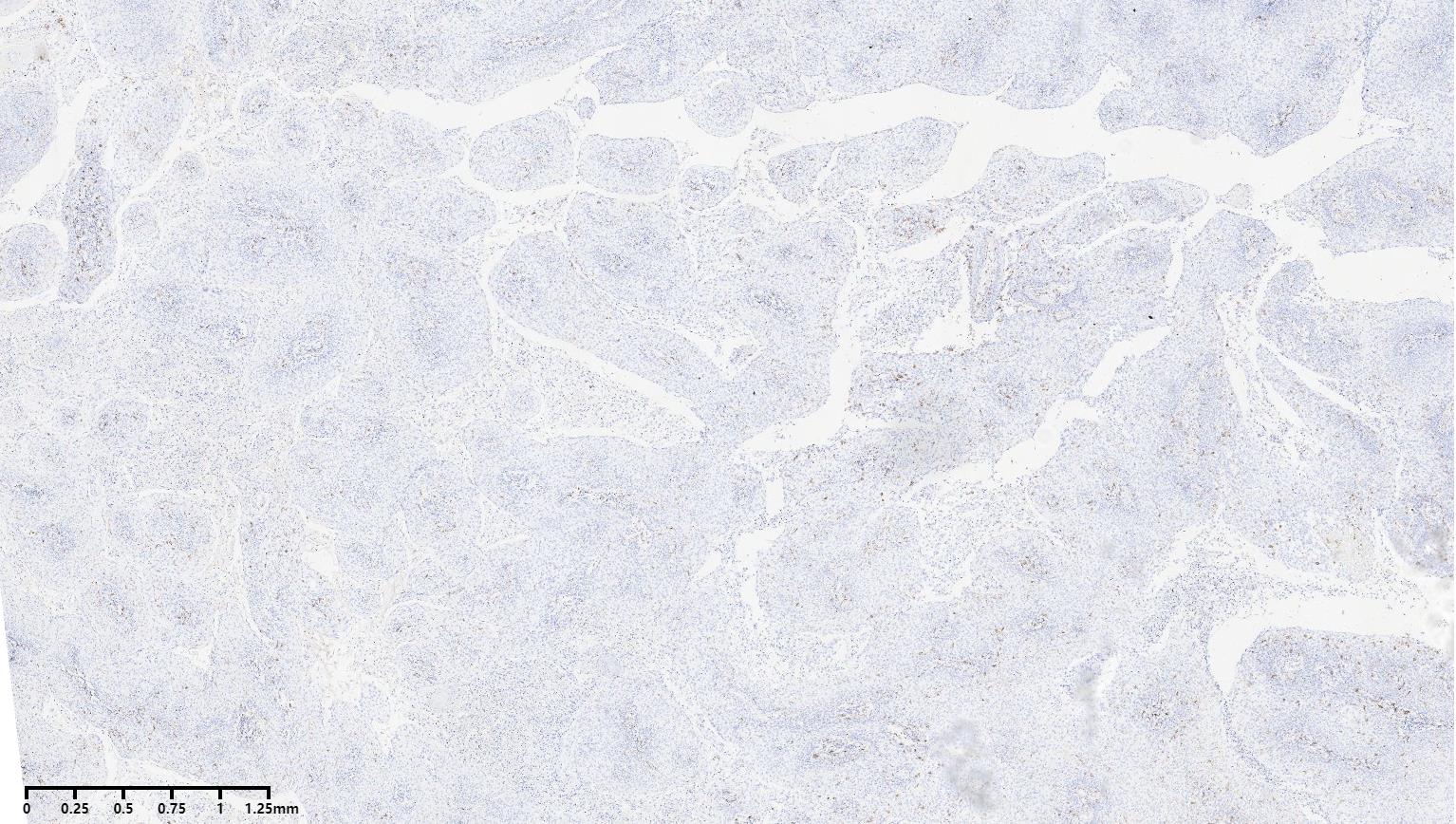

Supplement: Supplementary file 4 [file DataSheet3.zip › high NLR/817746---CD4_2.00X.jpg]

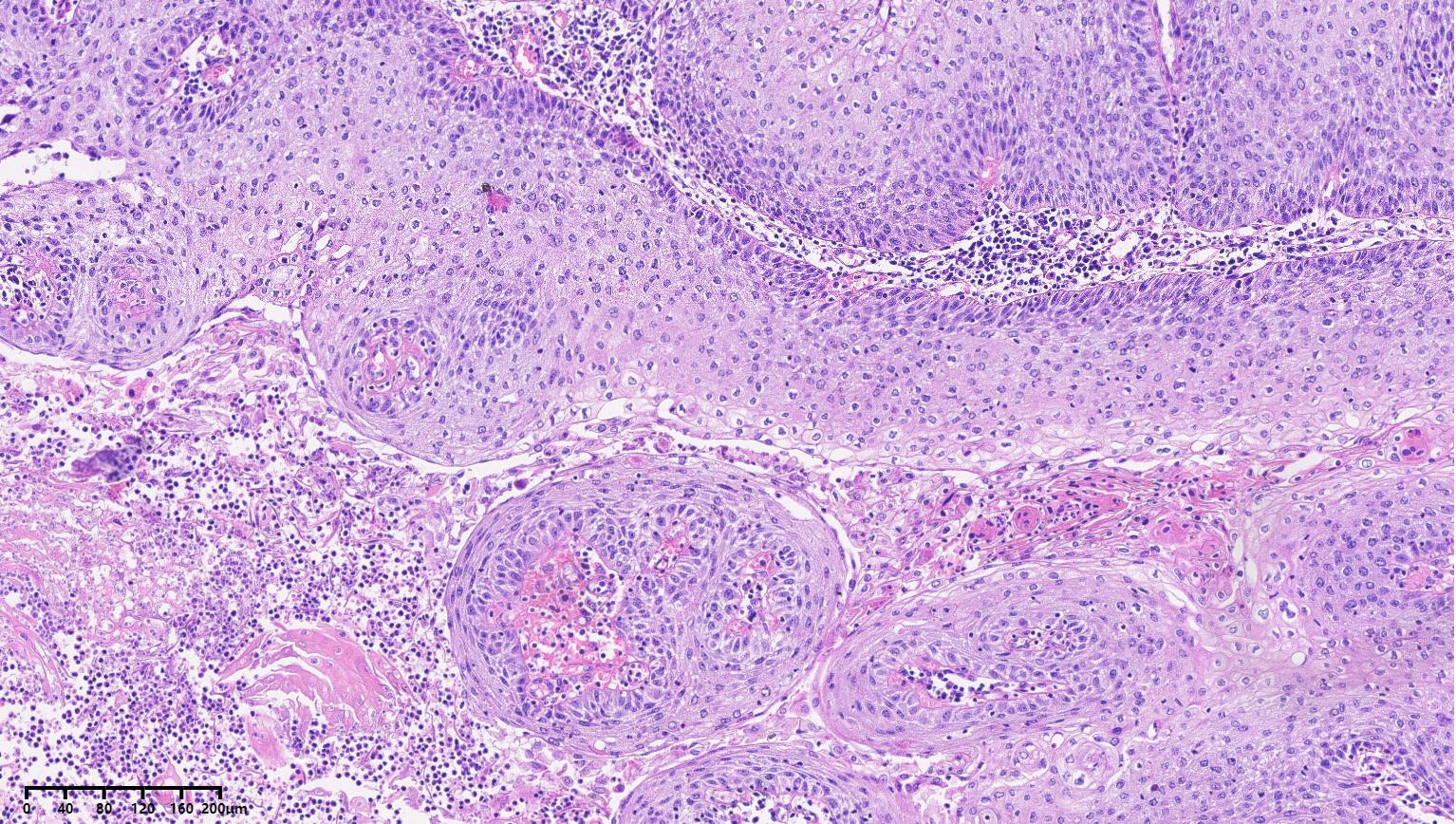

Supplement: Supplementary file 4 [file DataSheet3.zip › high NLR/817746(3%)_10.00X.jpg]

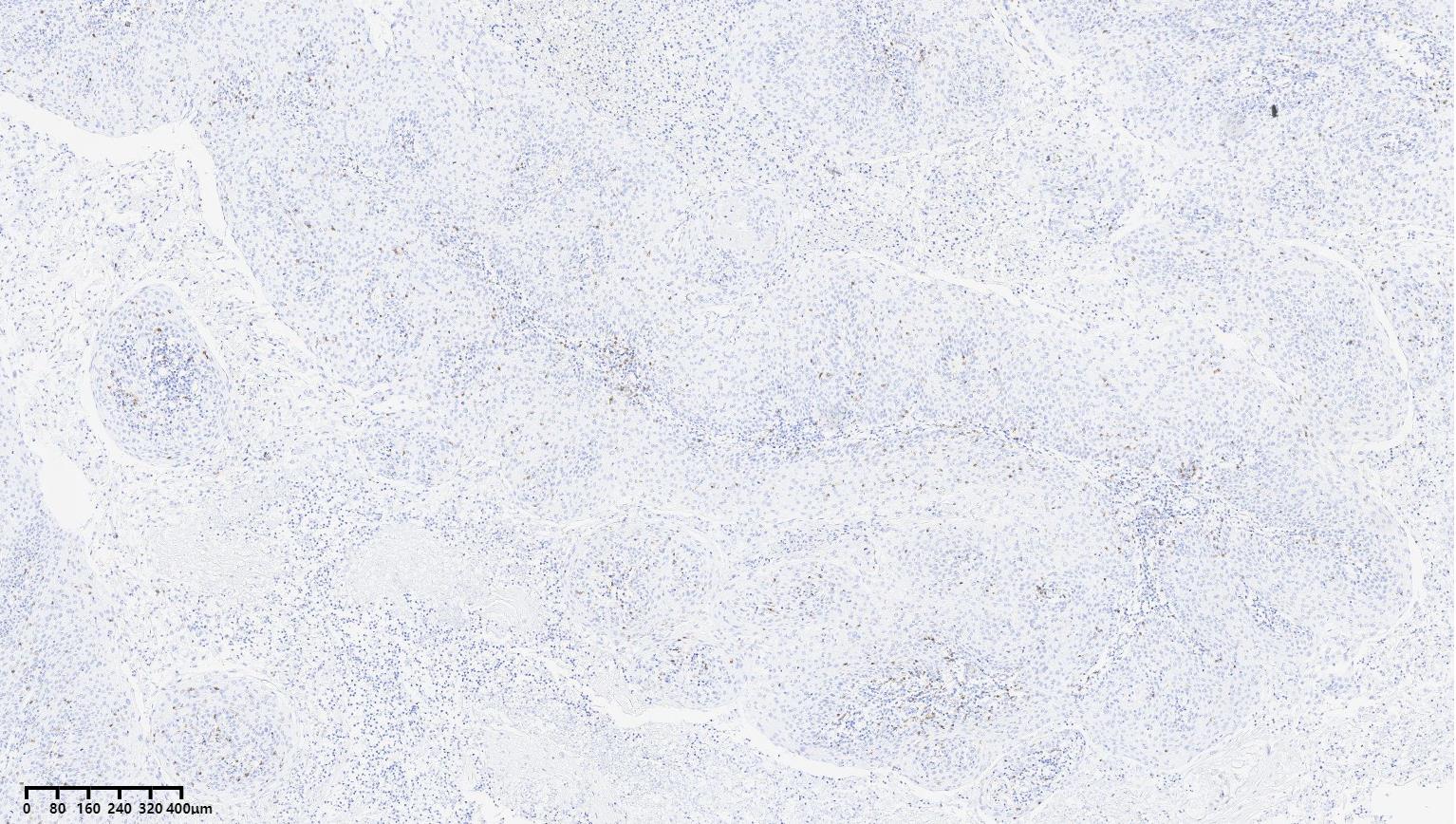

Supplement: Supplementary file 4 [file DataSheet3.zip › high NLR/817746--CD8_4.00X.jpg]

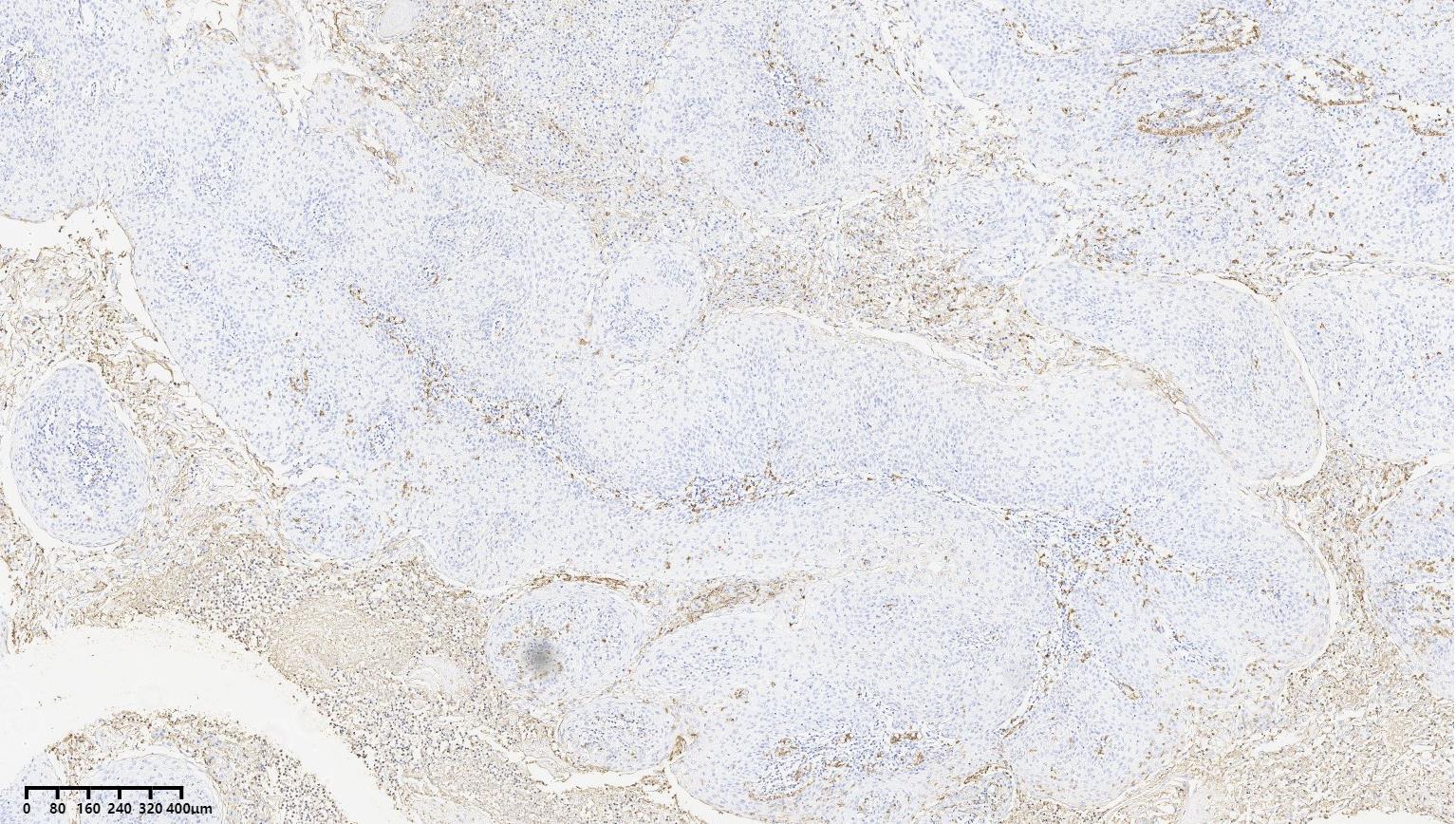

Supplement: Supplementary file 4 [file DataSheet3.zip › high NLR/817746---CD66b_4.00X.jpg]

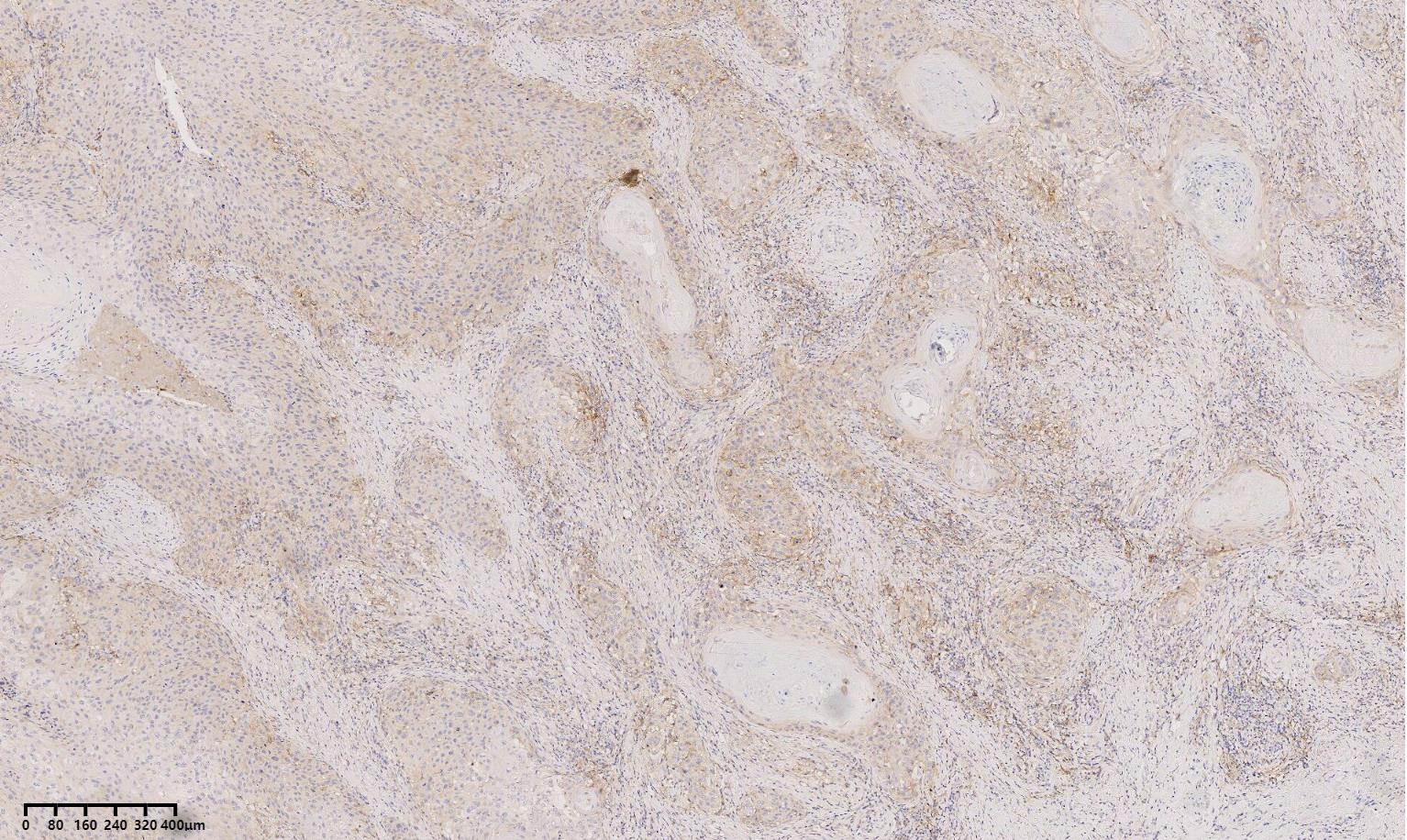

Supplement: Supplementary file 4 [file DataSheet3.zip › high NLR/MMP9_4.00X.jpg]

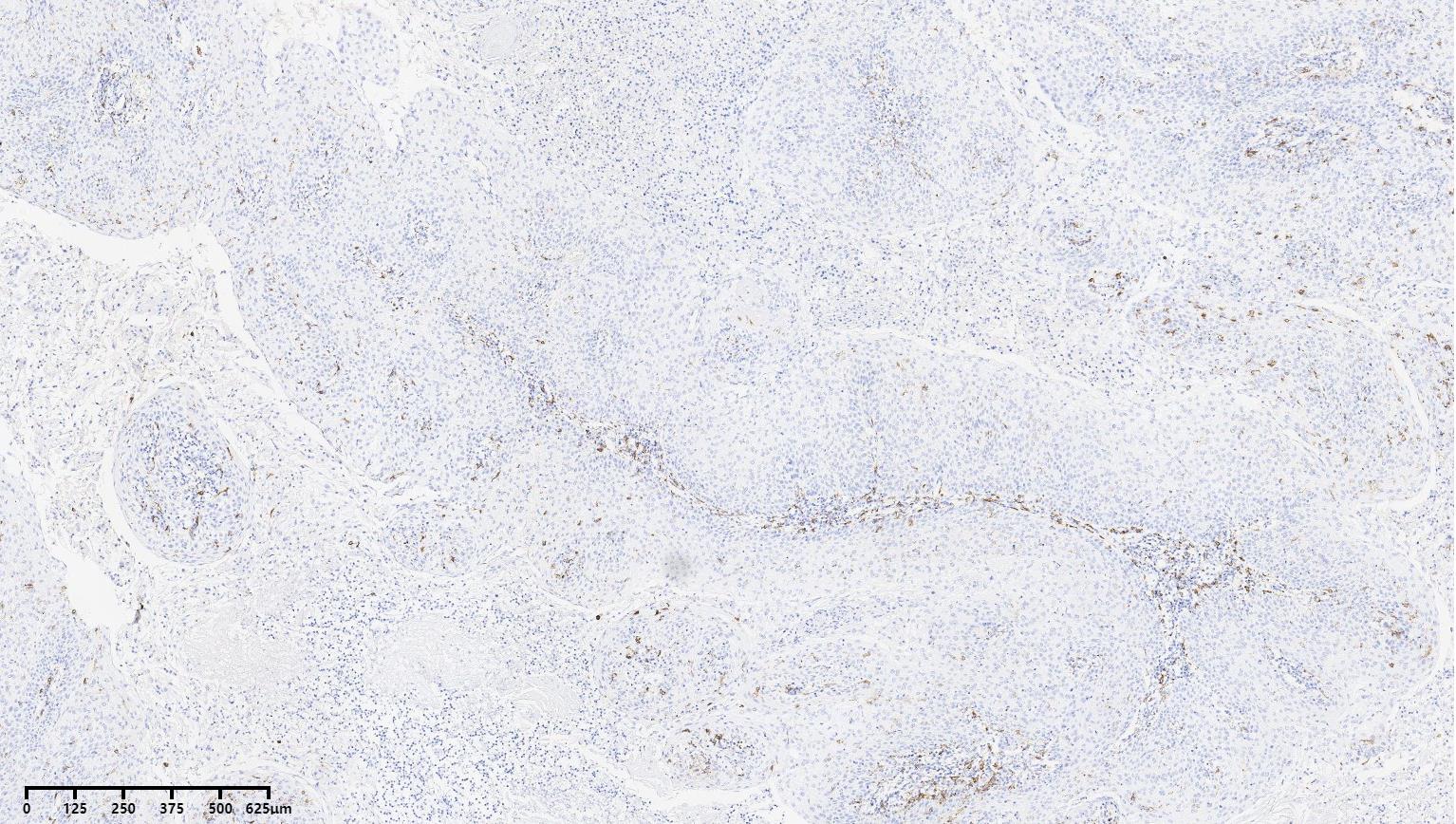

Supplement: Supplementary file 4 [file DataSheet3.zip › high NLR/817746---CD4_4.00X.jpg]

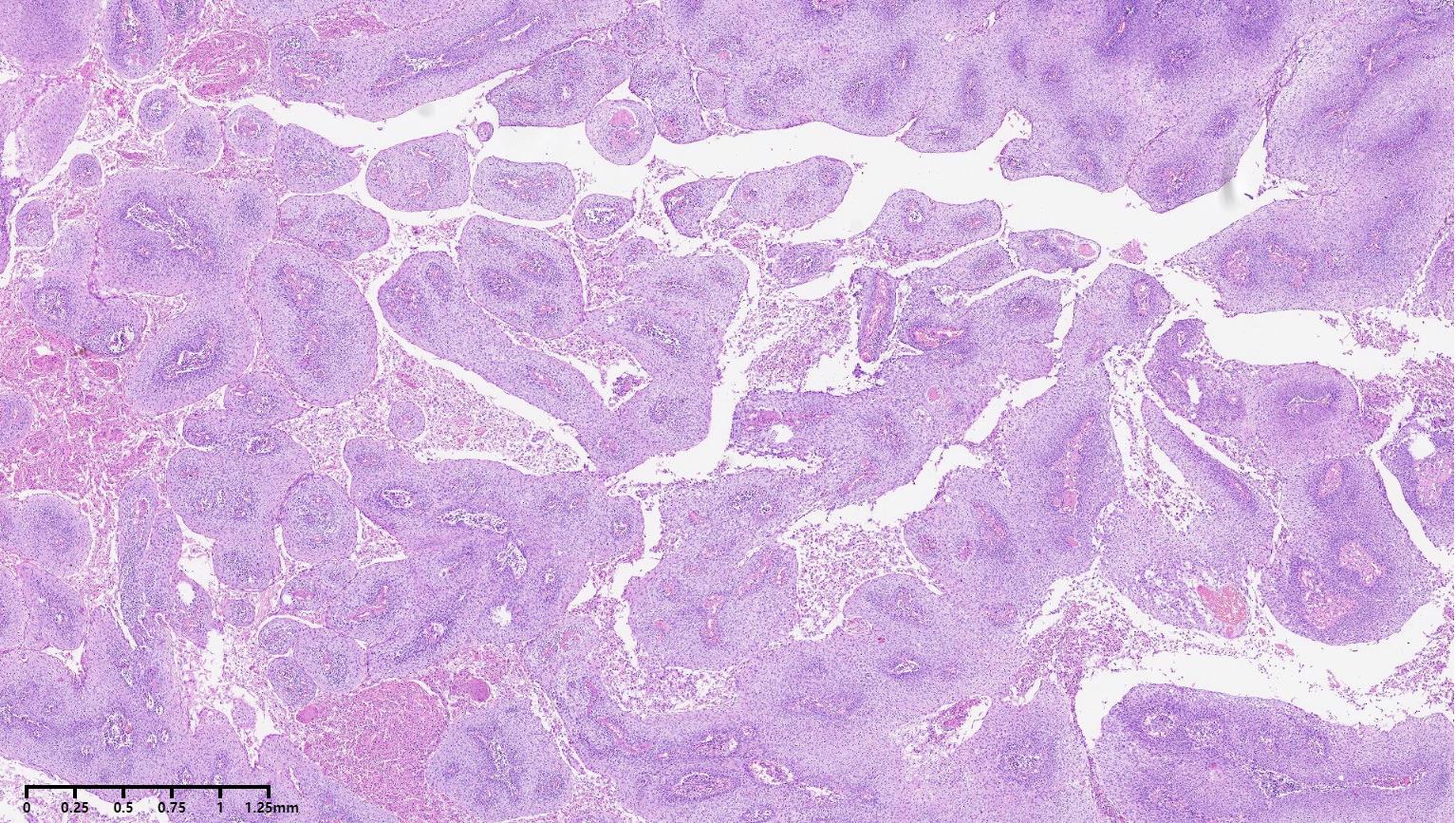

Supplement: Supplementary file 4 [file DataSheet3.zip › high NLR/817746(3%)_2.00X (3%TILs).jpg]

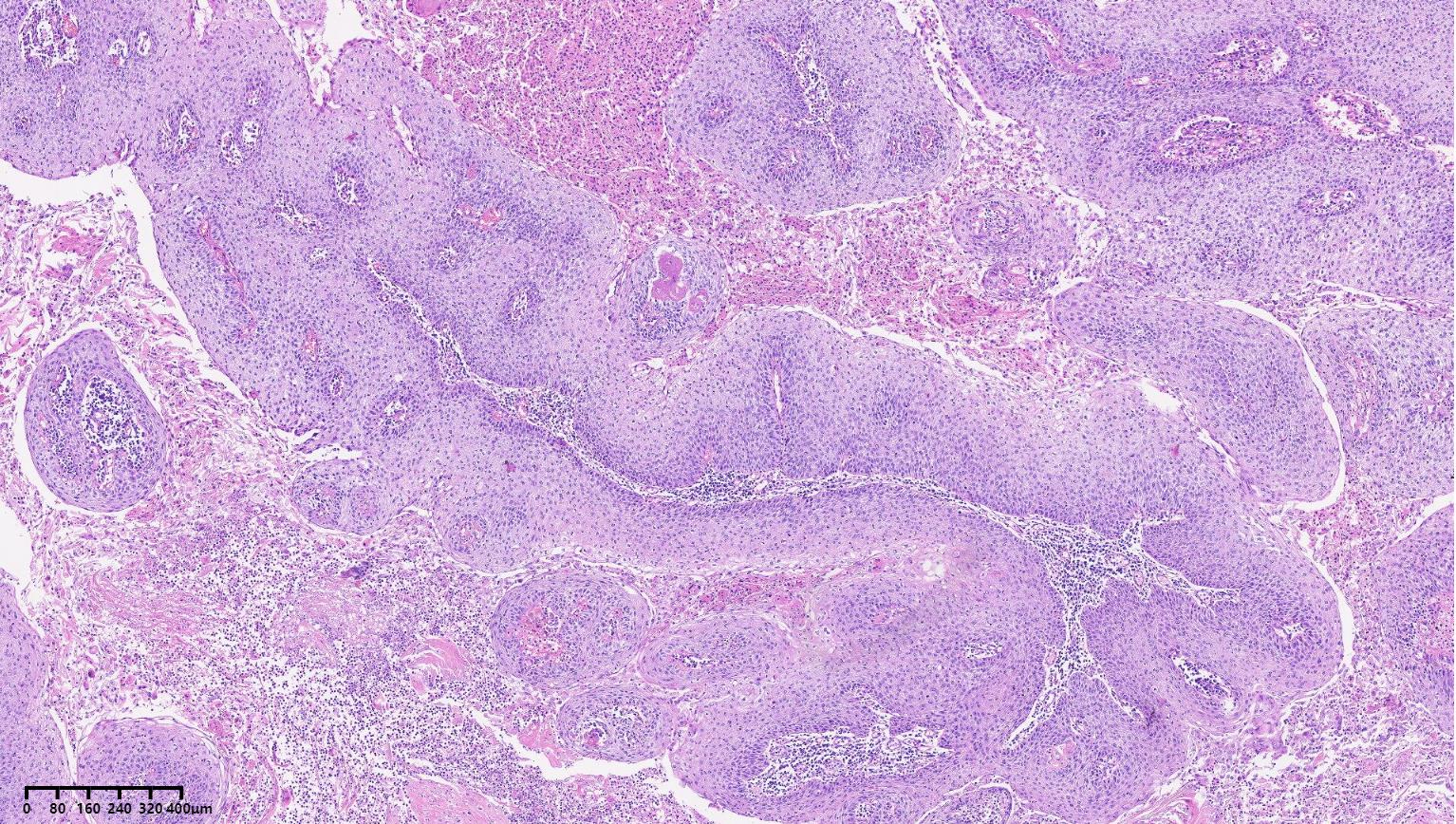

Supplement: Supplementary file 4 [file DataSheet3.zip › high NLR/817746(3%)_4.00X.jpg]

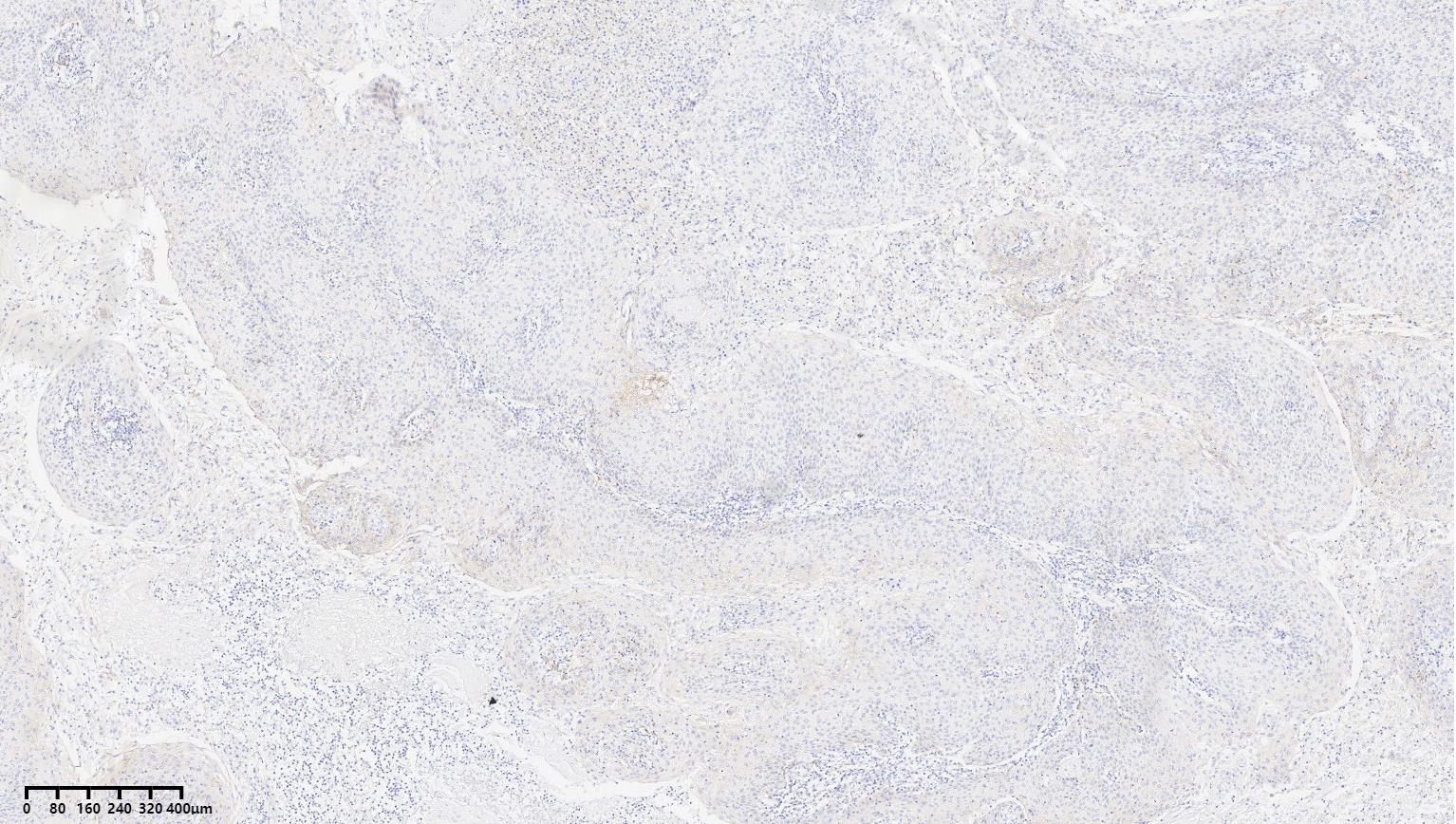

Supplement: Supplementary file 4 [file DataSheet3.zip › high NLR/817746--PDL1_4.00X.jpg]

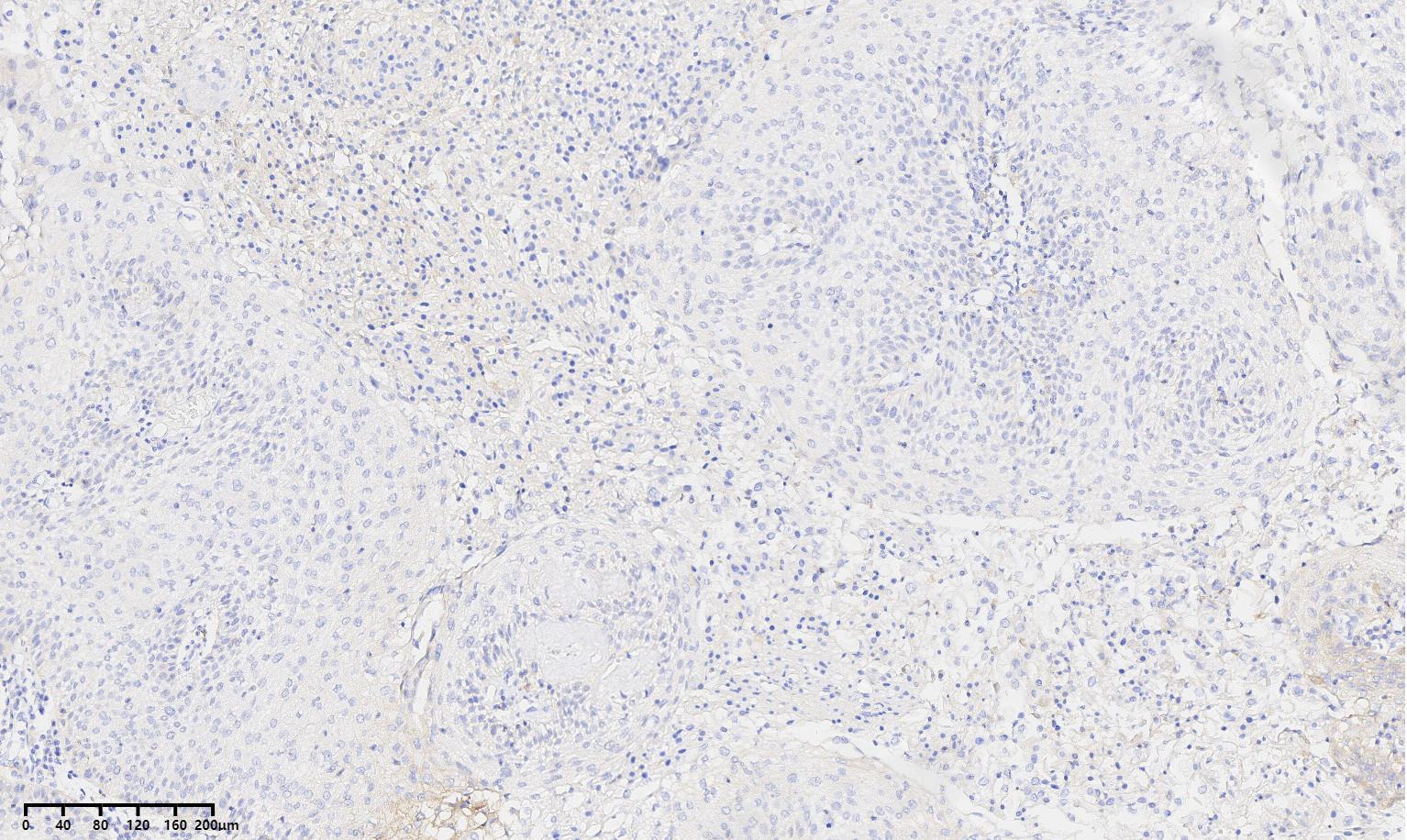

Supplement: Supplementary file 5 [file DataSheet4.zip › low NLR/MMP9_10.00X.jpg]

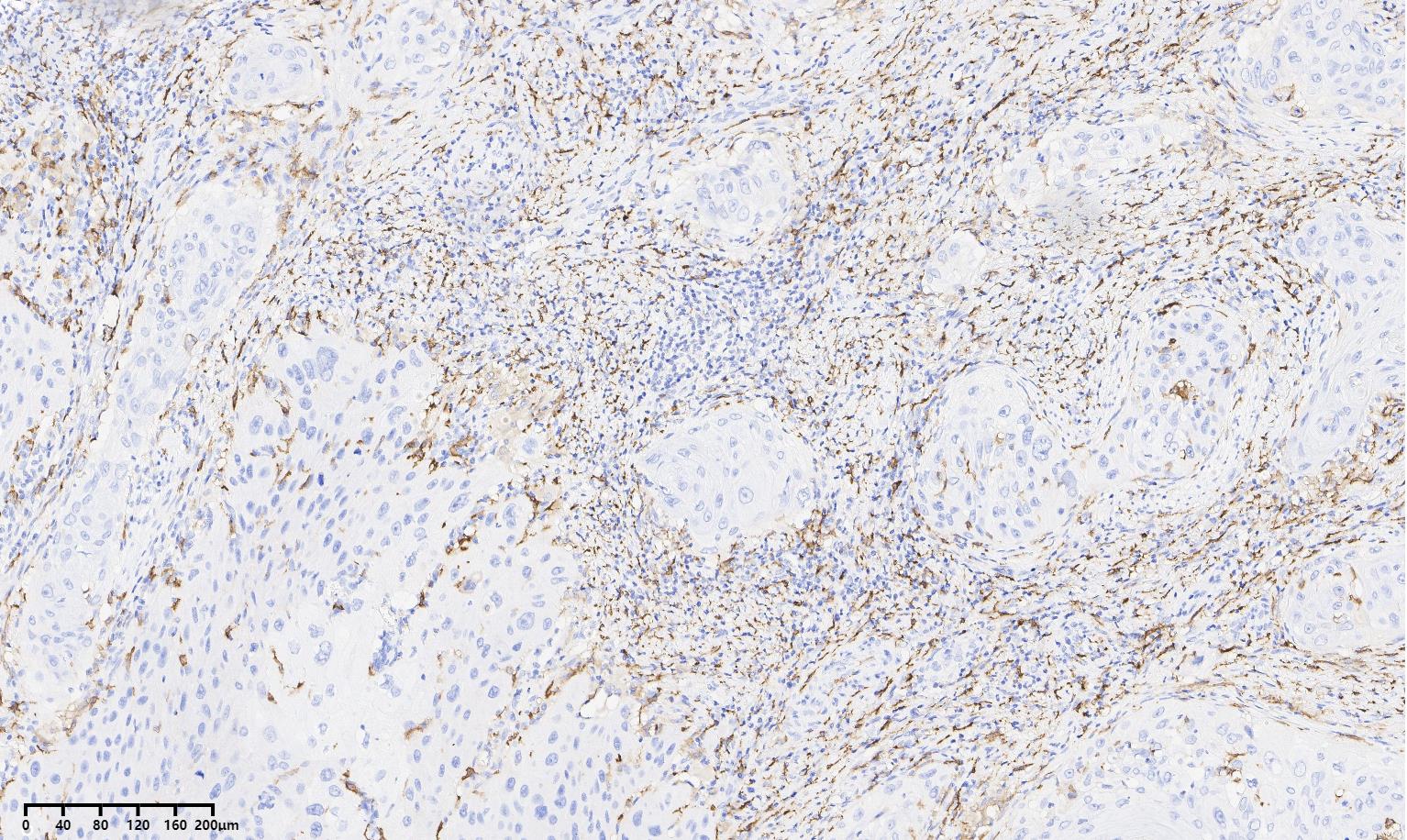

Supplement: Supplementary file 5 [file DataSheet4.zip › low NLR/794771--CD4_10.00X.jpg]

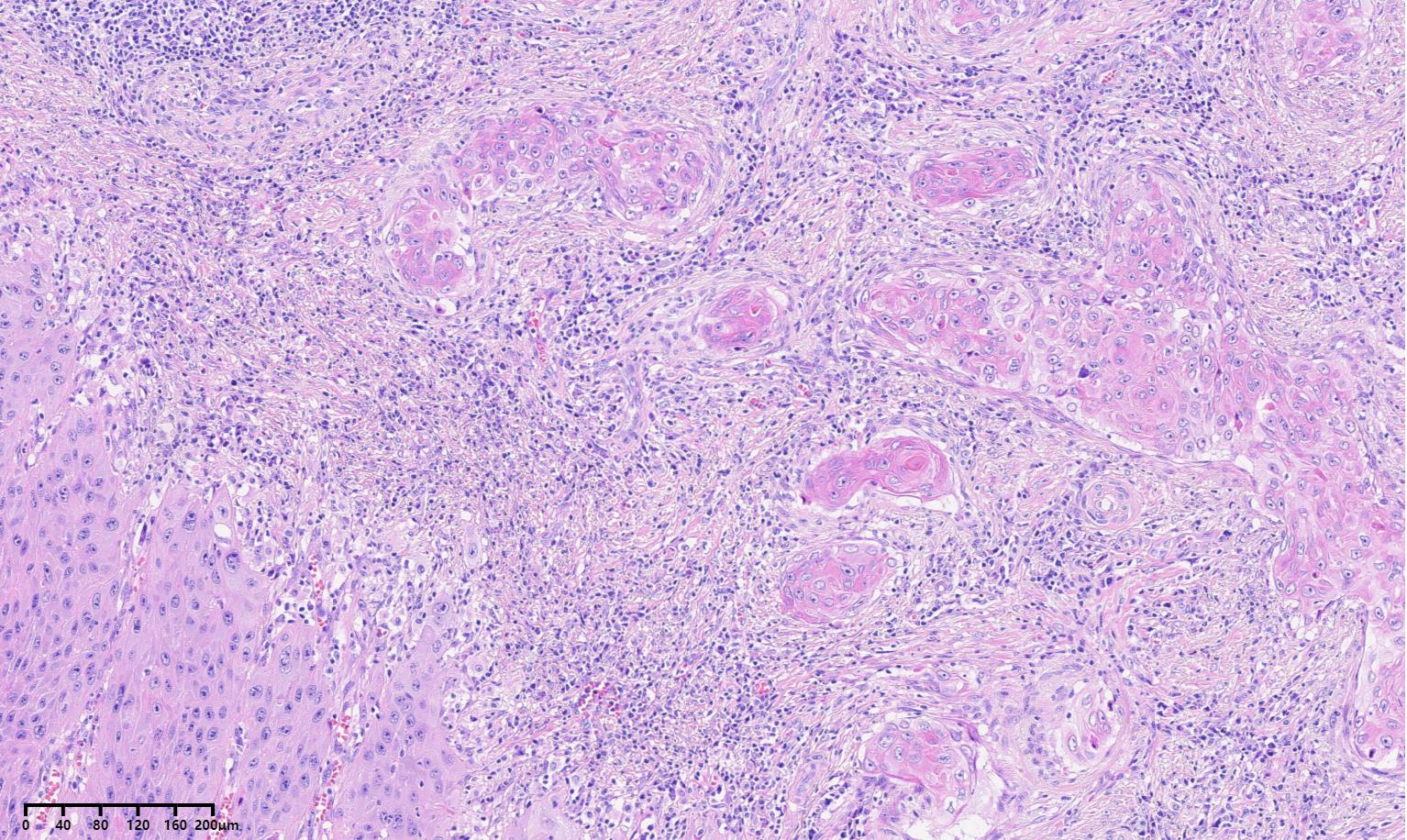

Supplement: Supplementary file 5 [file DataSheet4.zip › low NLR/794771(20%)_10.00X.jpg]

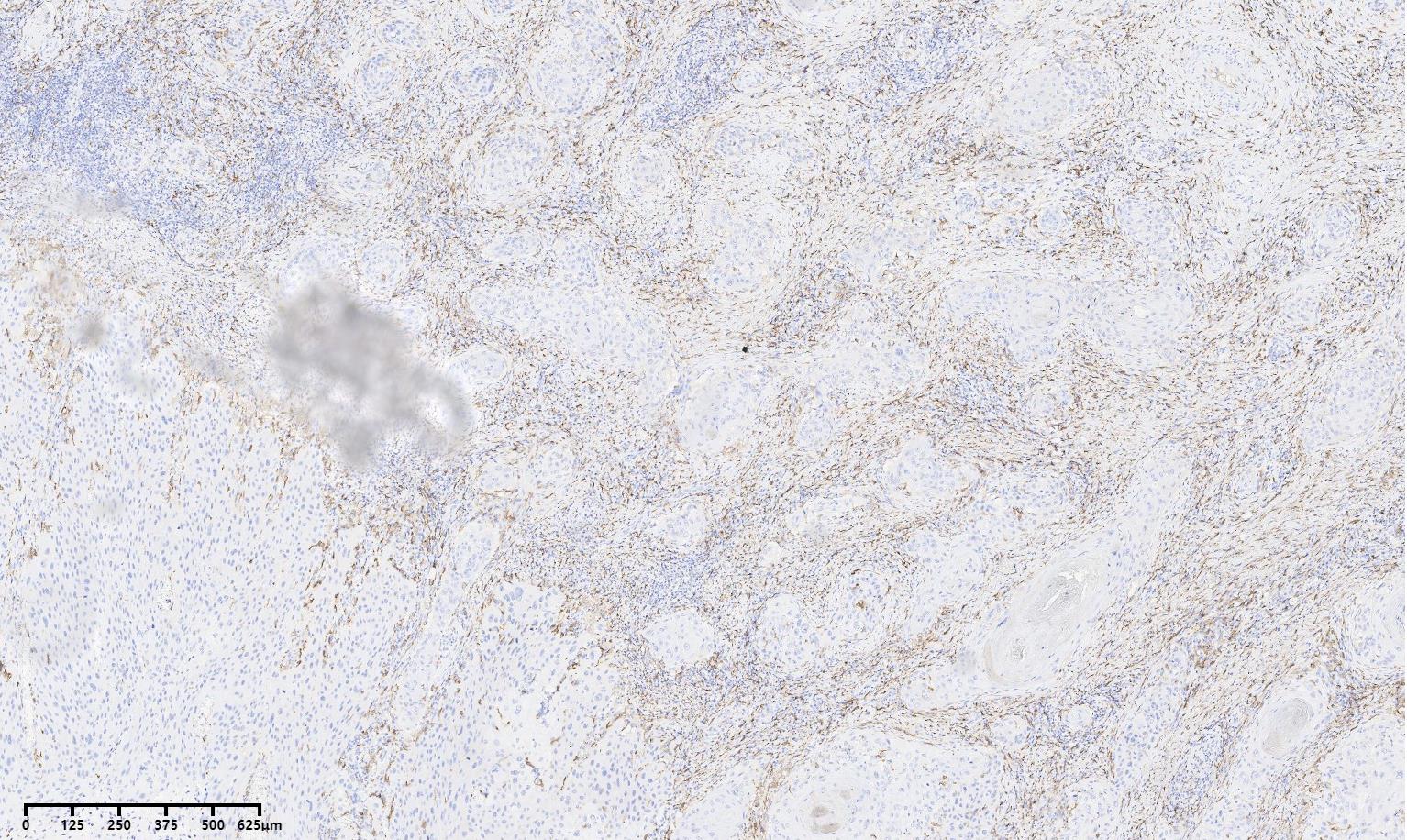

Supplement: Supplementary file 5 [file DataSheet4.zip › low NLR/794771--CD4_4.00X.jpg]

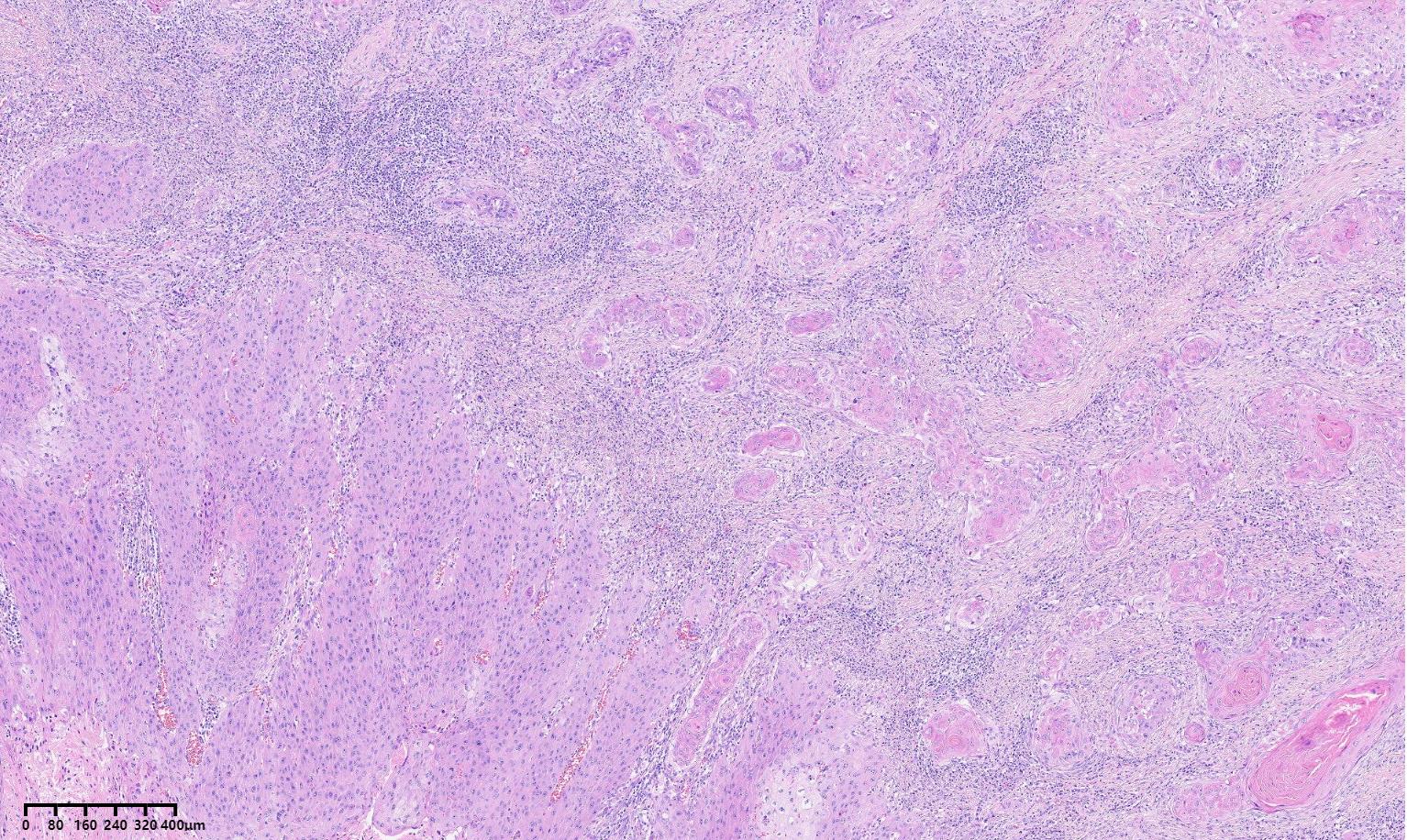

Supplement: Supplementary file 5 [file DataSheet4.zip › low NLR/794771(20%)_4.00X.jpg]

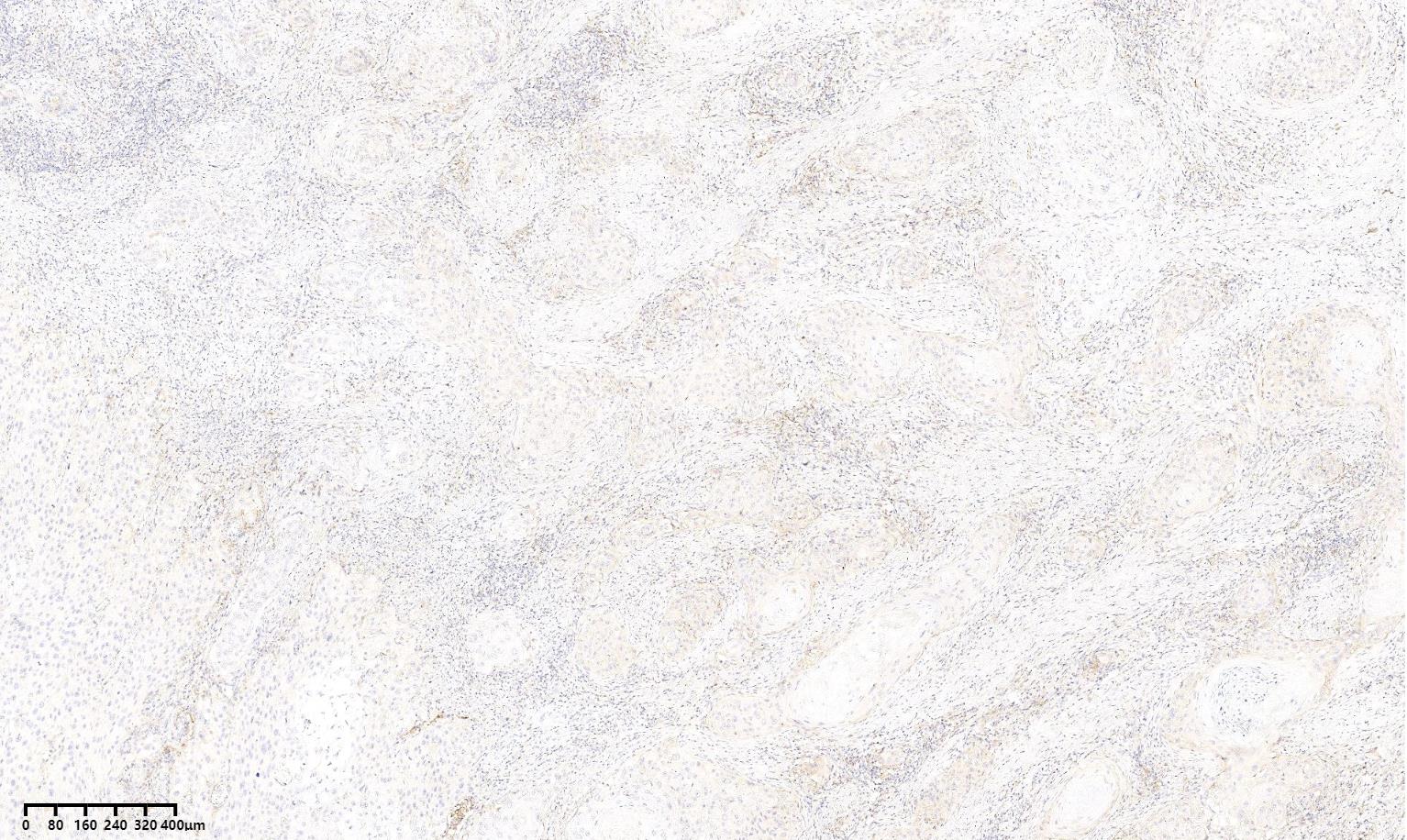

Supplement: Supplementary file 5 [file DataSheet4.zip › low NLR/794771--PDL1_4.00X.jpg]

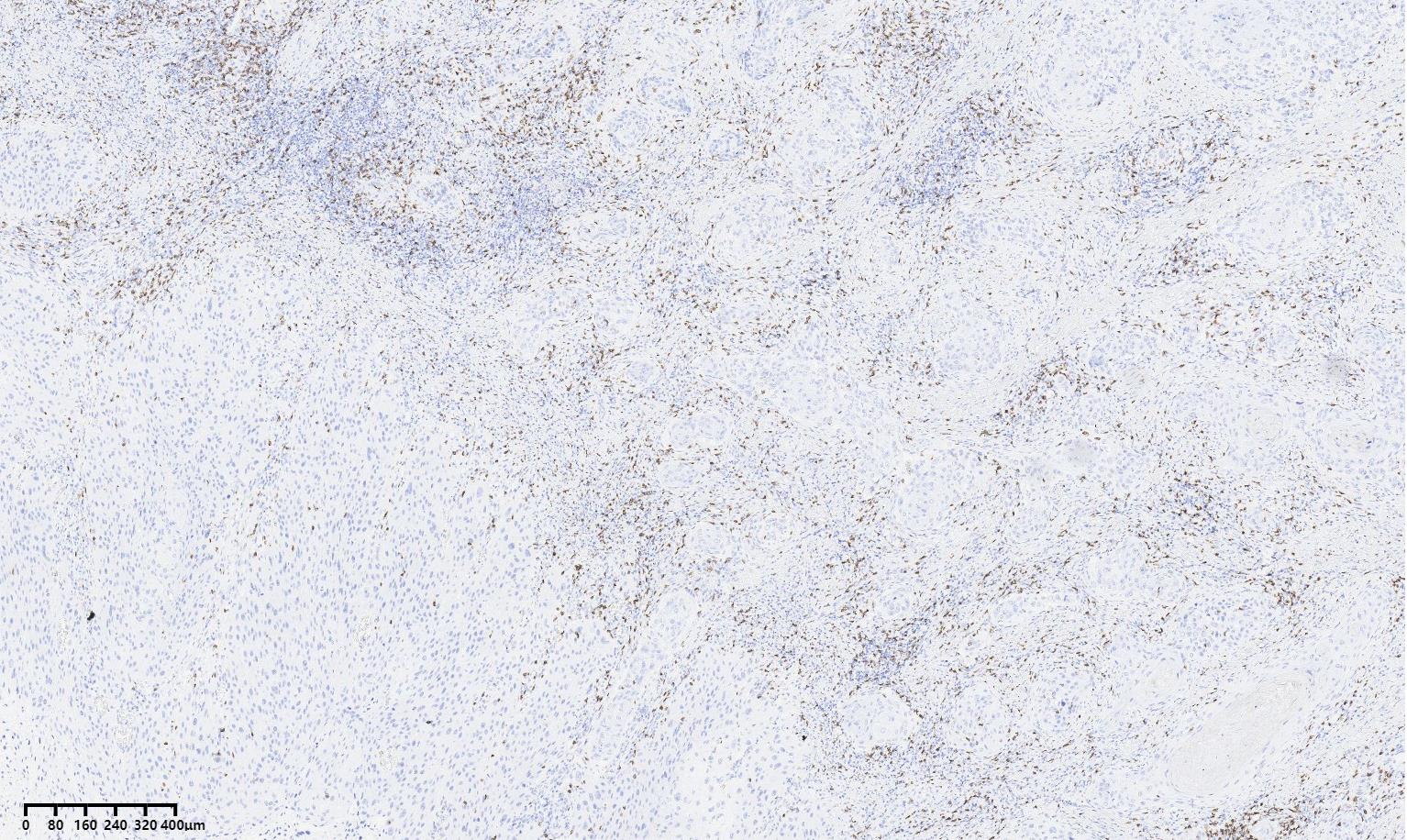

Supplement: Supplementary file 5 [file DataSheet4.zip › low NLR/794771--CD8_4.00X.jpg]

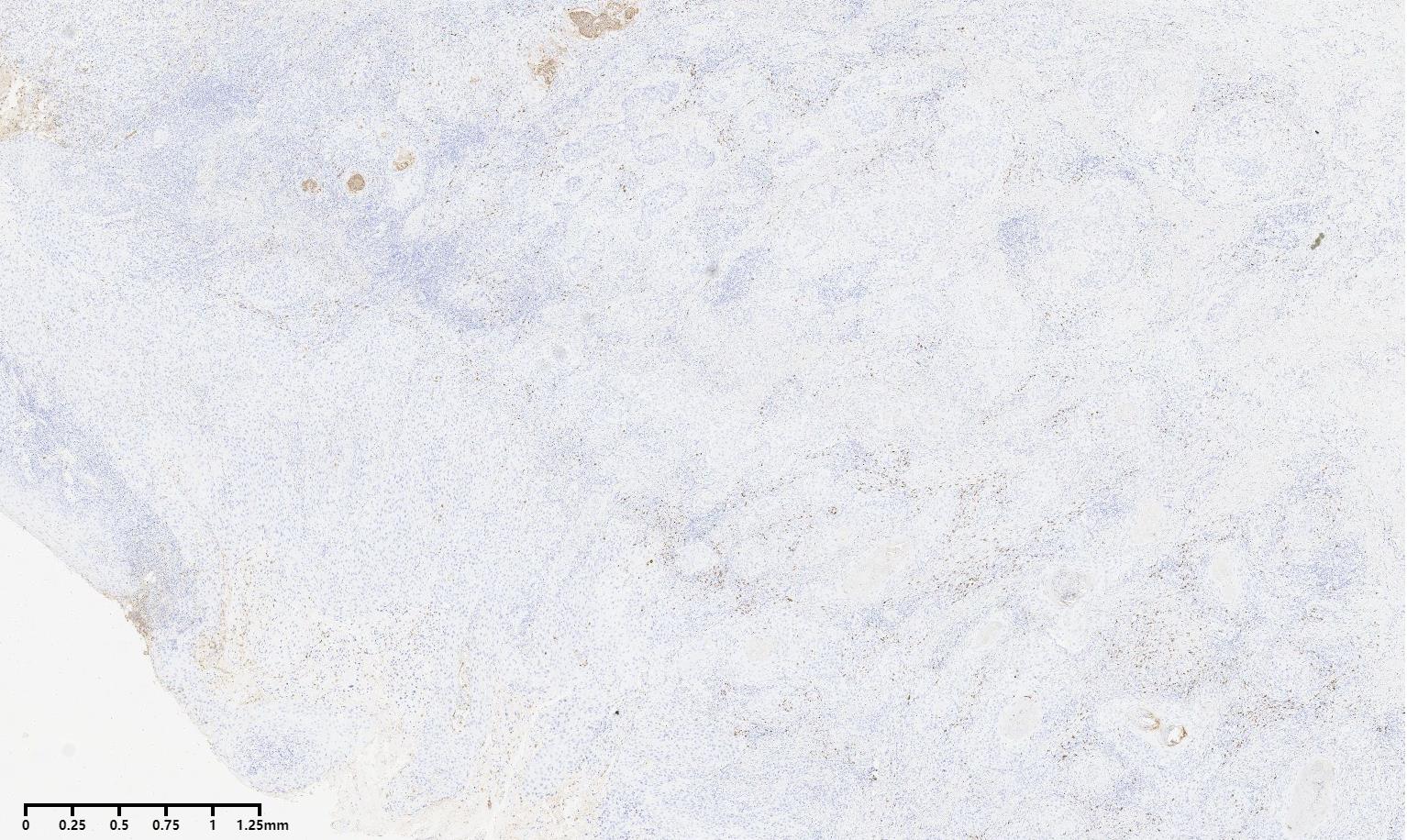

Supplement: Supplementary file 5 [file DataSheet4.zip › low NLR/794771--CD66b_2.00X.jpg]

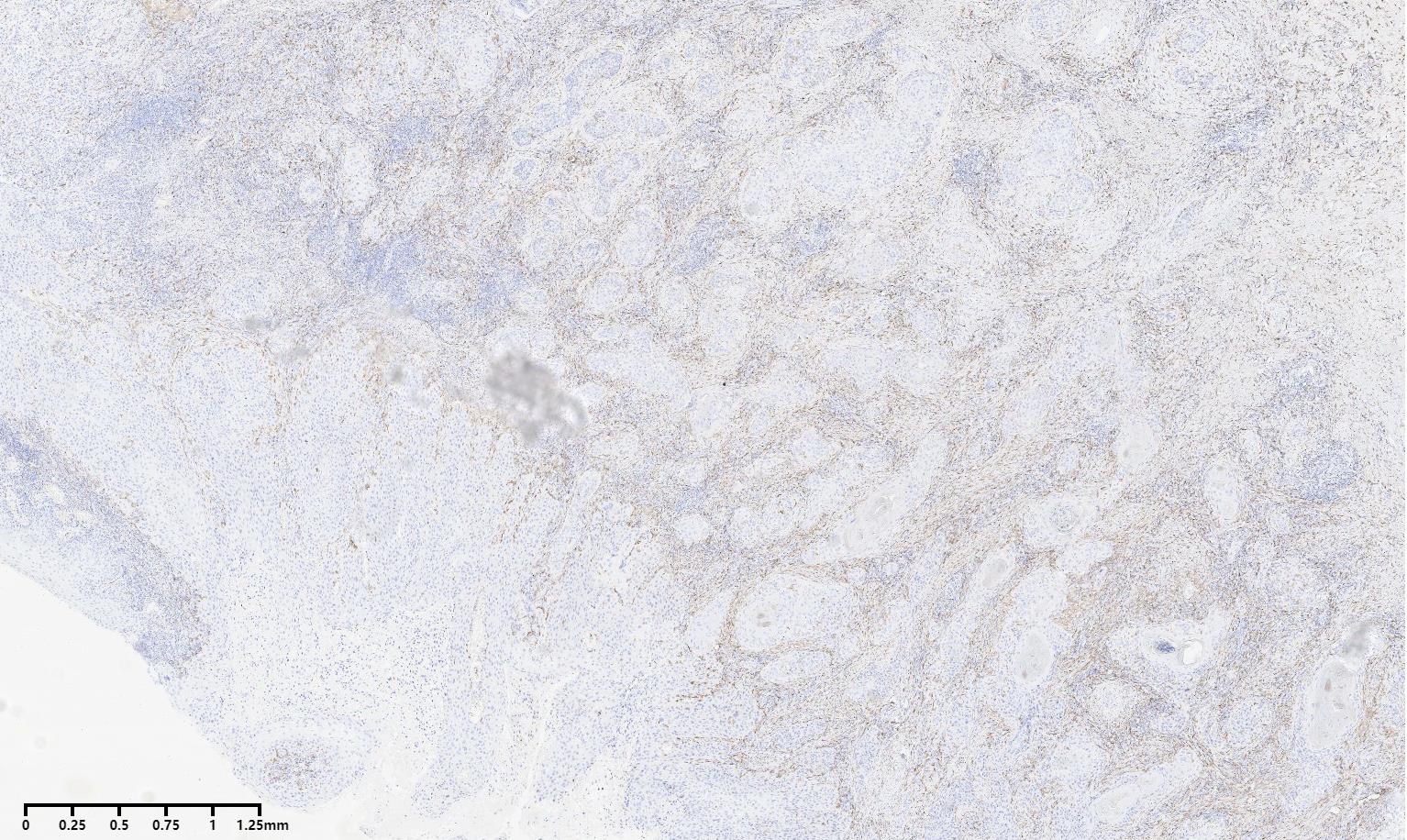

Supplement: Supplementary file 5 [file DataSheet4.zip › low NLR/794771--CD4_2.00X.jpg]

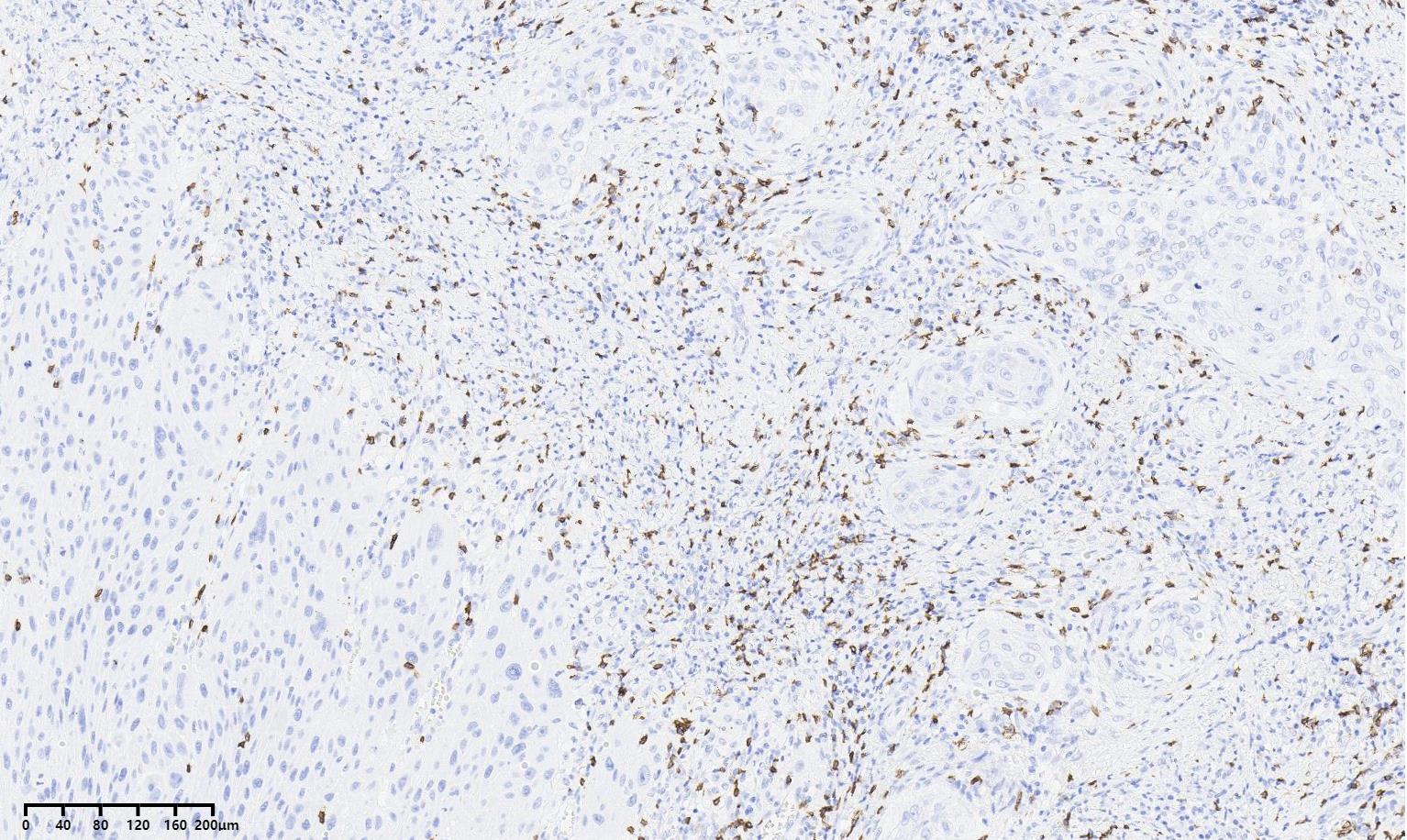

Supplement: Supplementary file 5 [file DataSheet4.zip › low NLR/794771--CD8_10.00X.jpg]

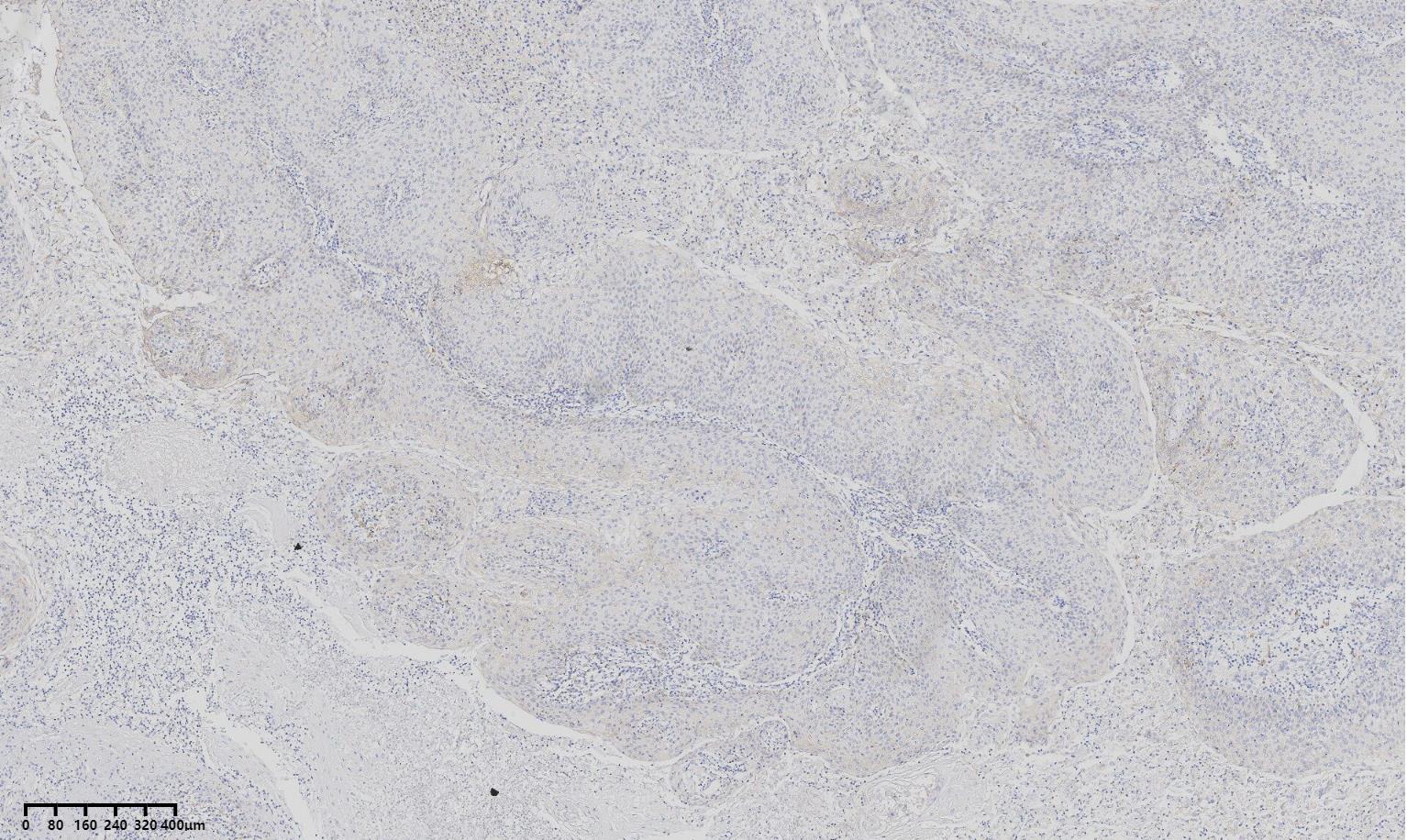

Supplement: Supplementary file 5 [file DataSheet4.zip › low NLR/MMP9_4.00X.jpg]

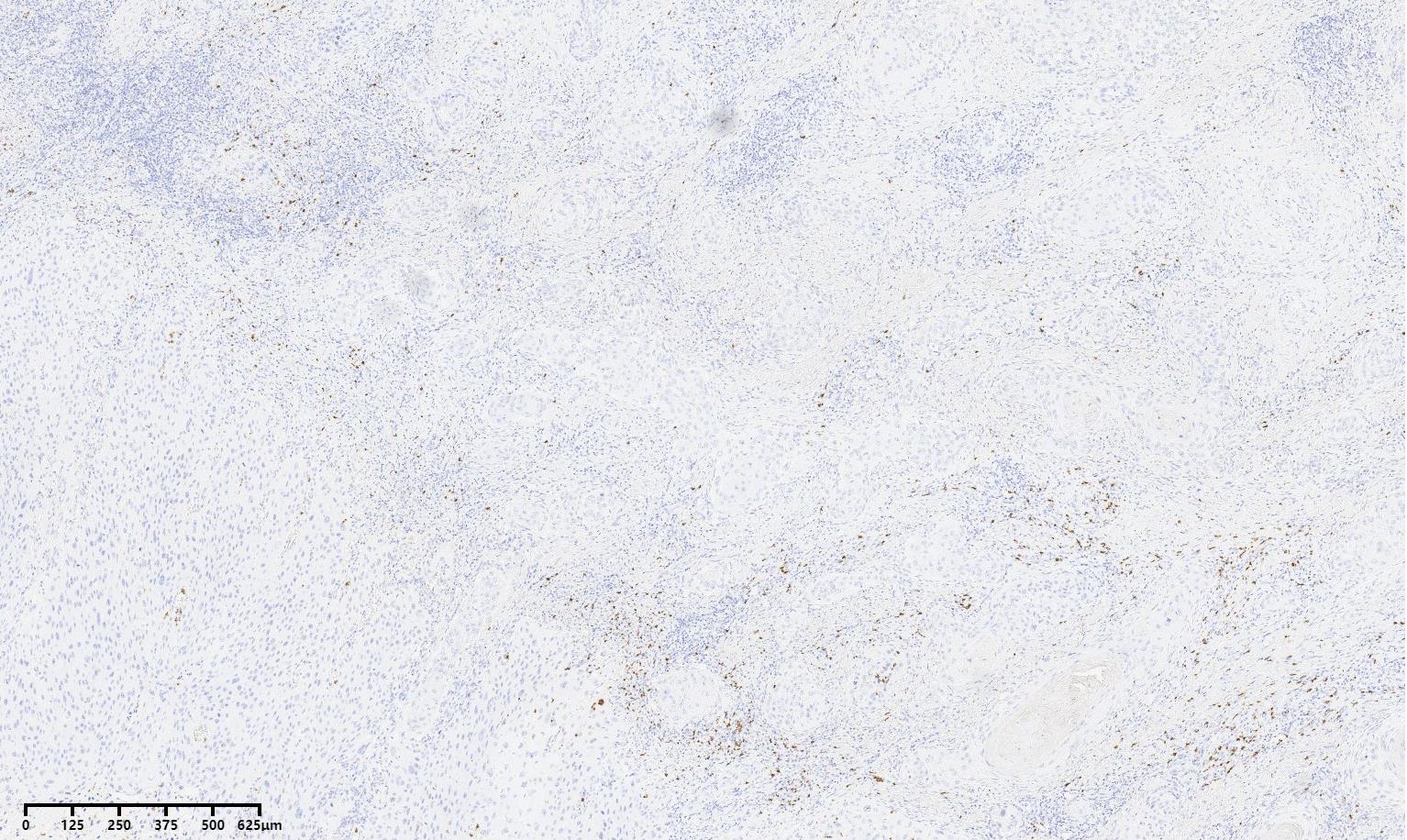

Supplement: Supplementary file 5 [file DataSheet4.zip › low NLR/794771--CD66b_4.00X.jpg]

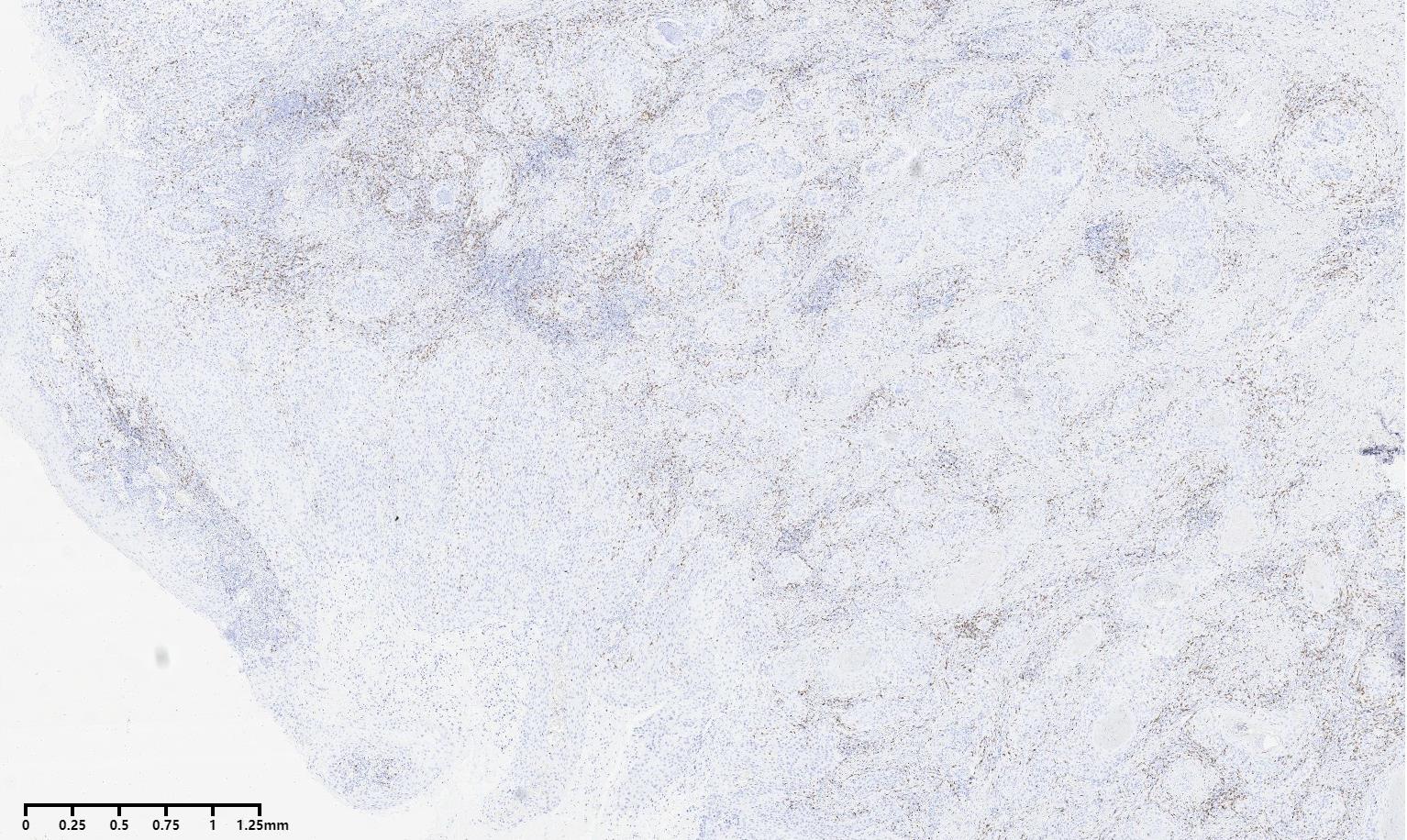

Supplement: Supplementary file 5 [file DataSheet4.zip › low NLR/794771--CD8_2.00X.jpg]

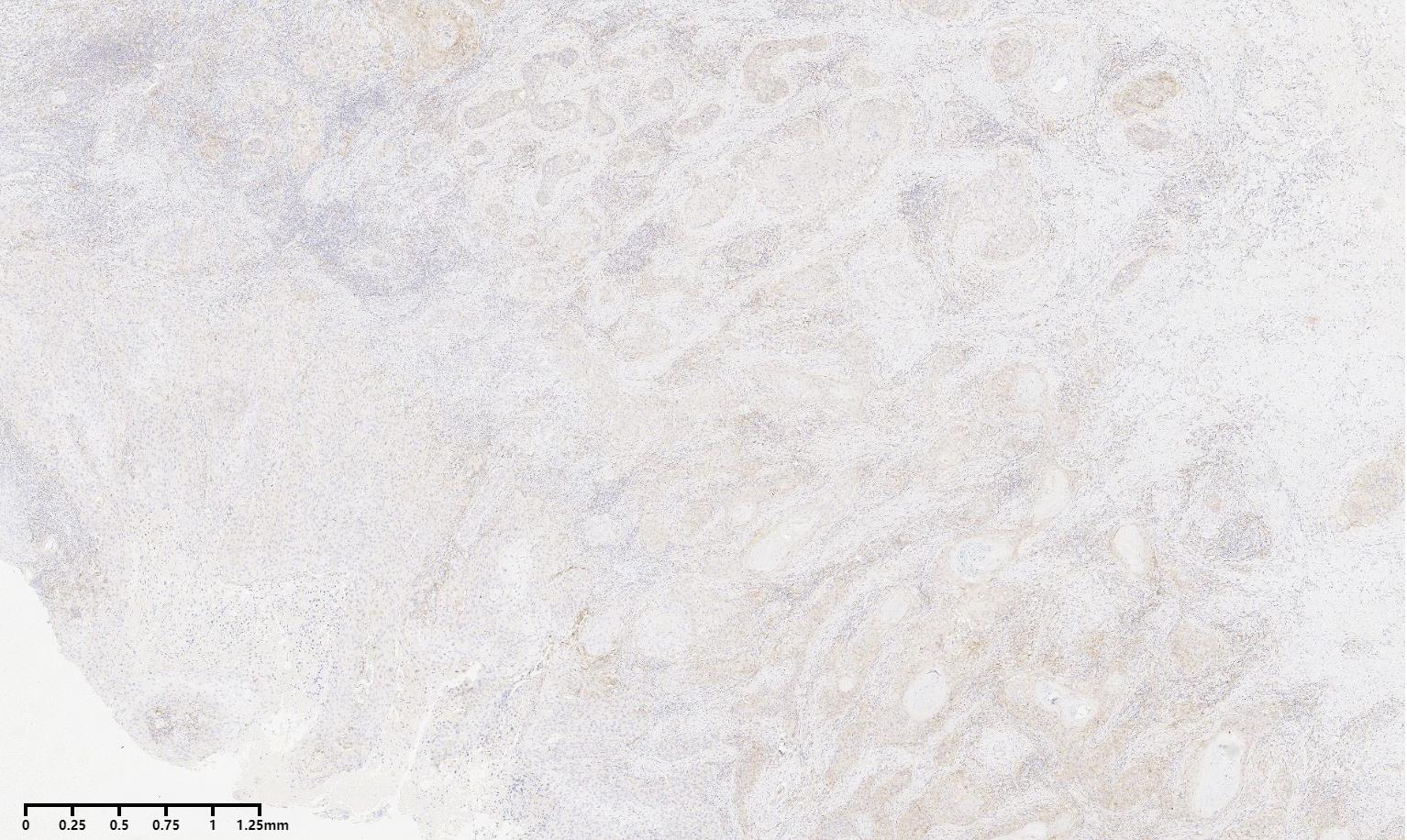

Supplement: Supplementary file 5 [file DataSheet4.zip › low NLR/794771--PDL1_2.00X.jpg]

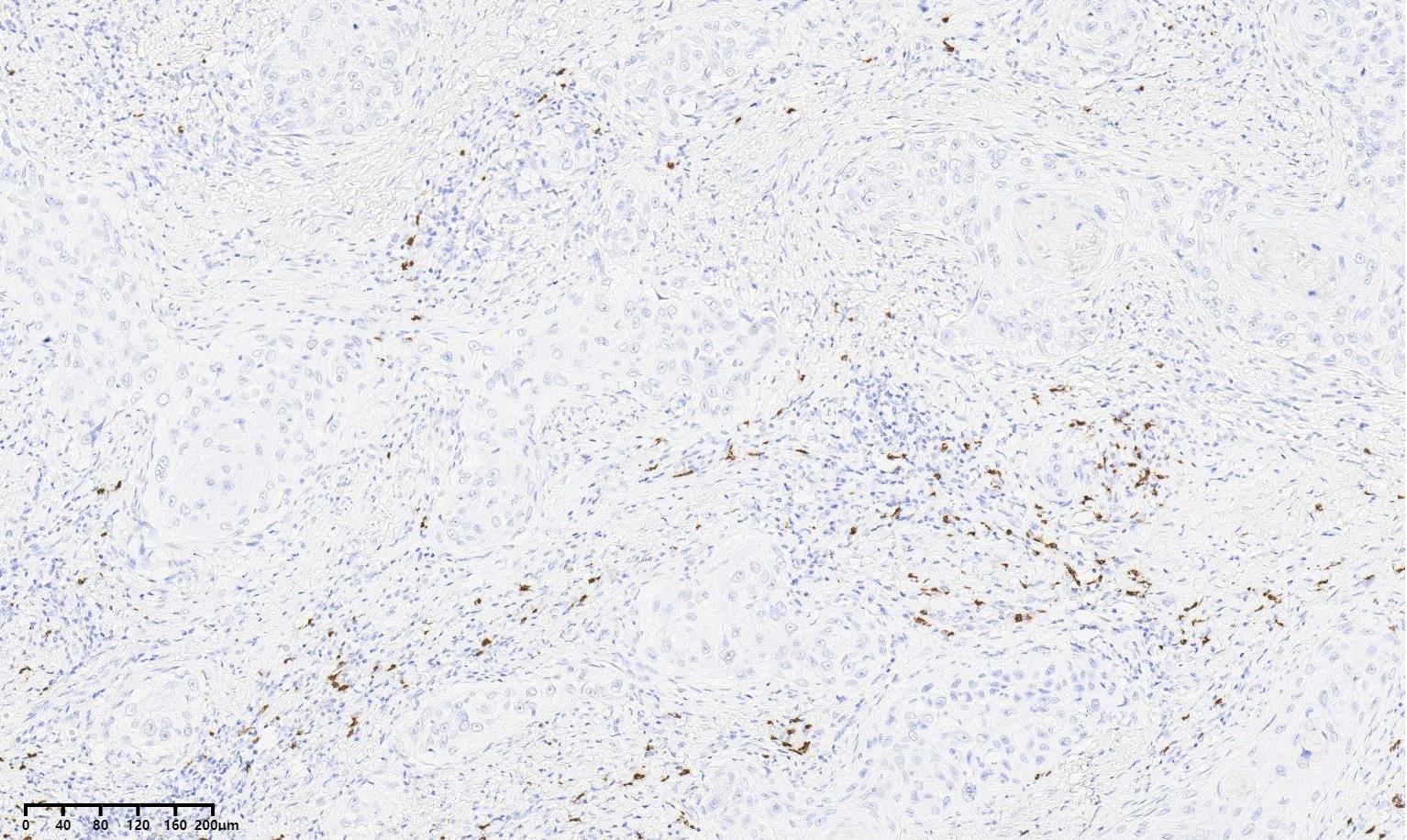

Supplement: Supplementary file 5 [file DataSheet4.zip › low NLR/794771--CD66b_10.00X.jpg]

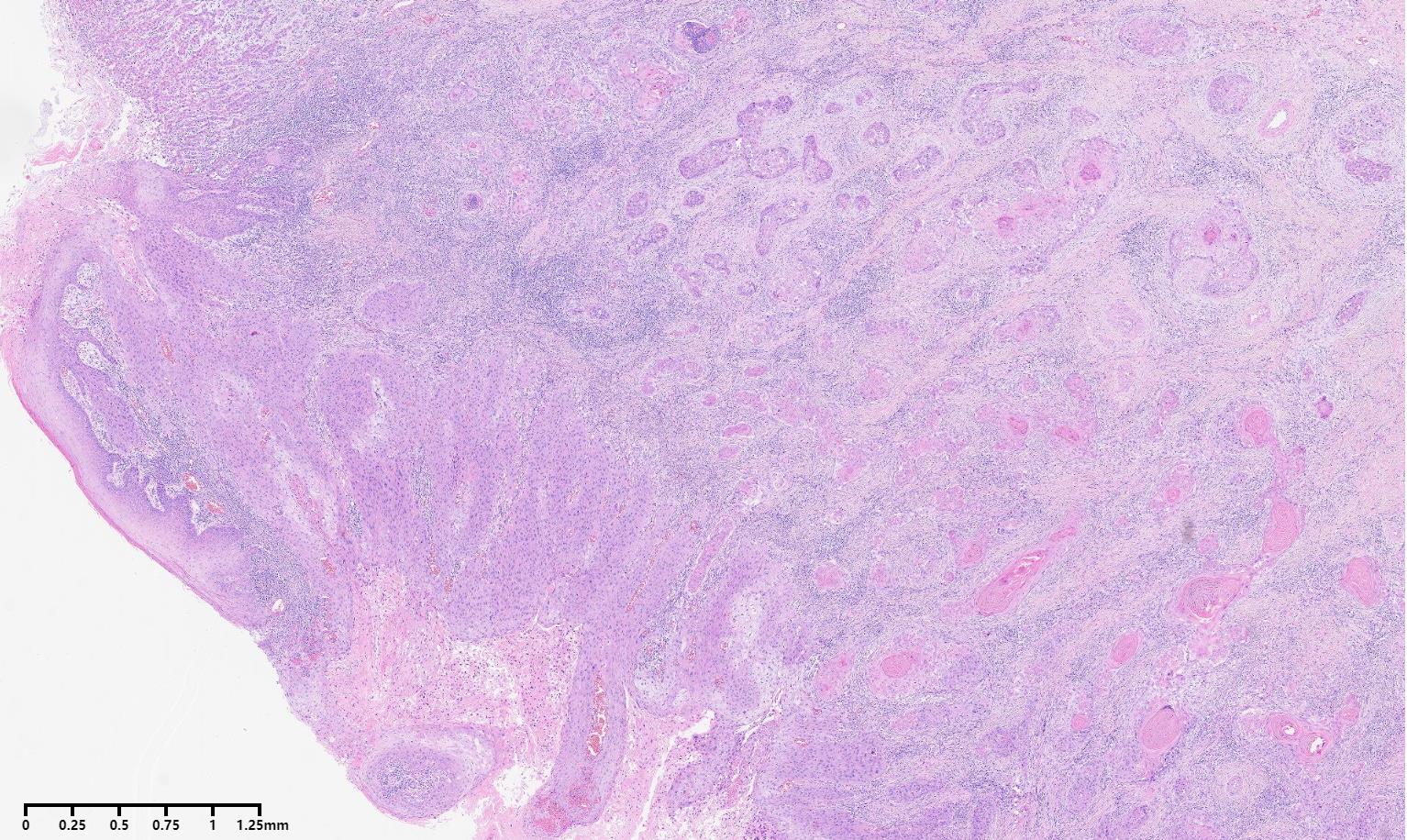

Supplement: Supplementary file 5 [file DataSheet4.zip › low NLR/794771(20%)_2.00X.jpg]

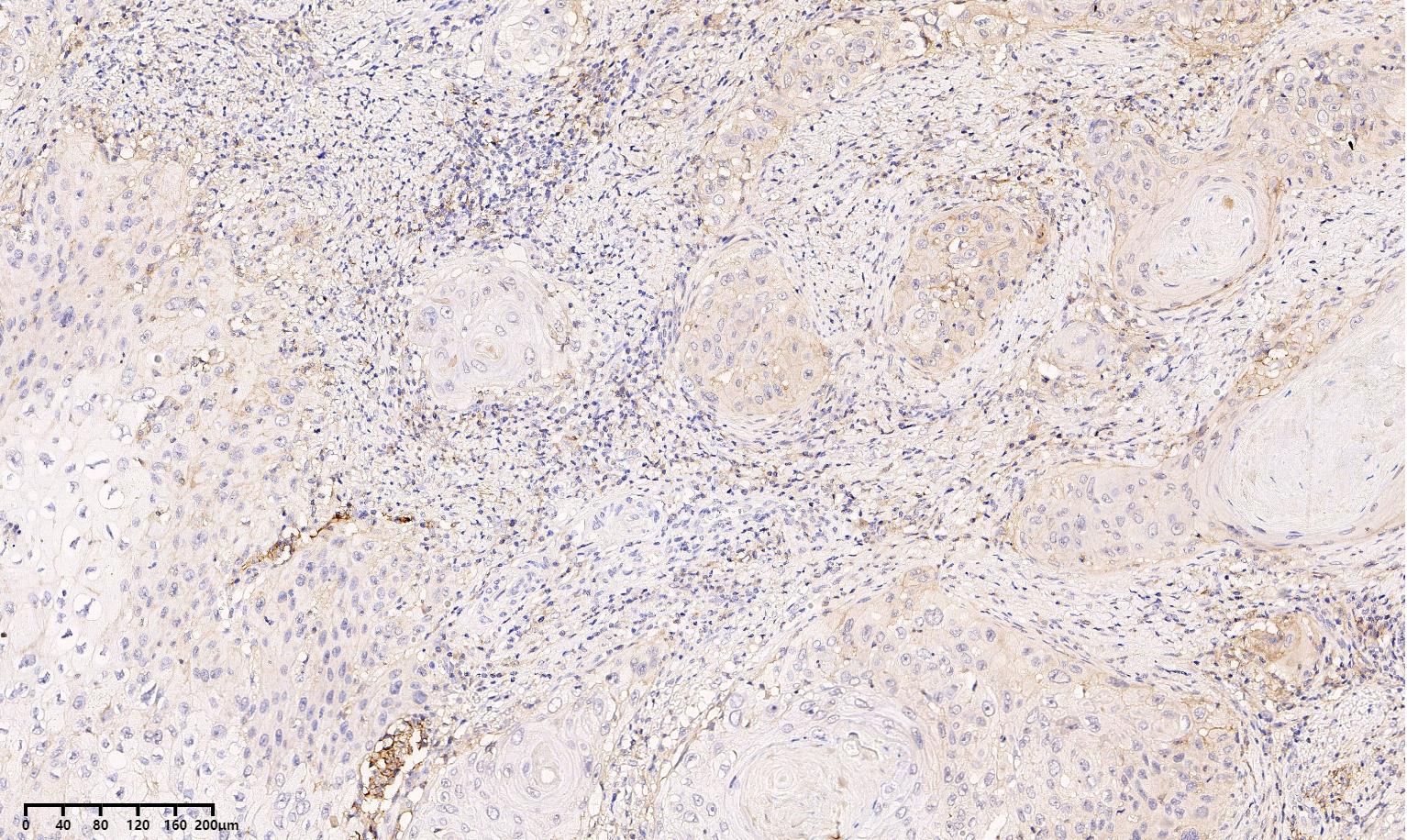

Supplement: Supplementary file 5 [file DataSheet4.zip › low NLR/794771--PDL1_10.00X.jpg]

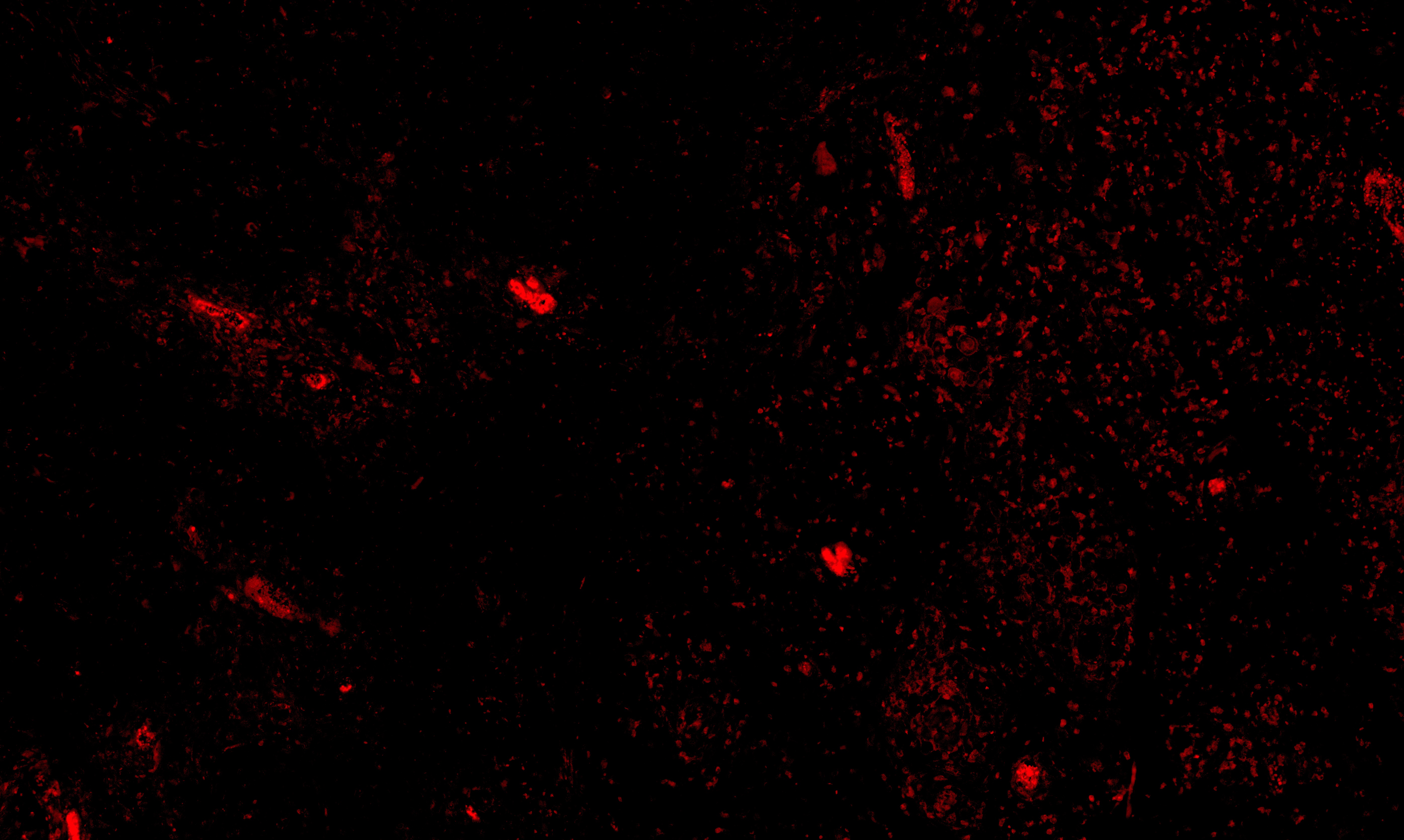

Supplement: Supplementary file 6 [file DataSheet5.zip › NETs-1/NETs_MPO.jpg]

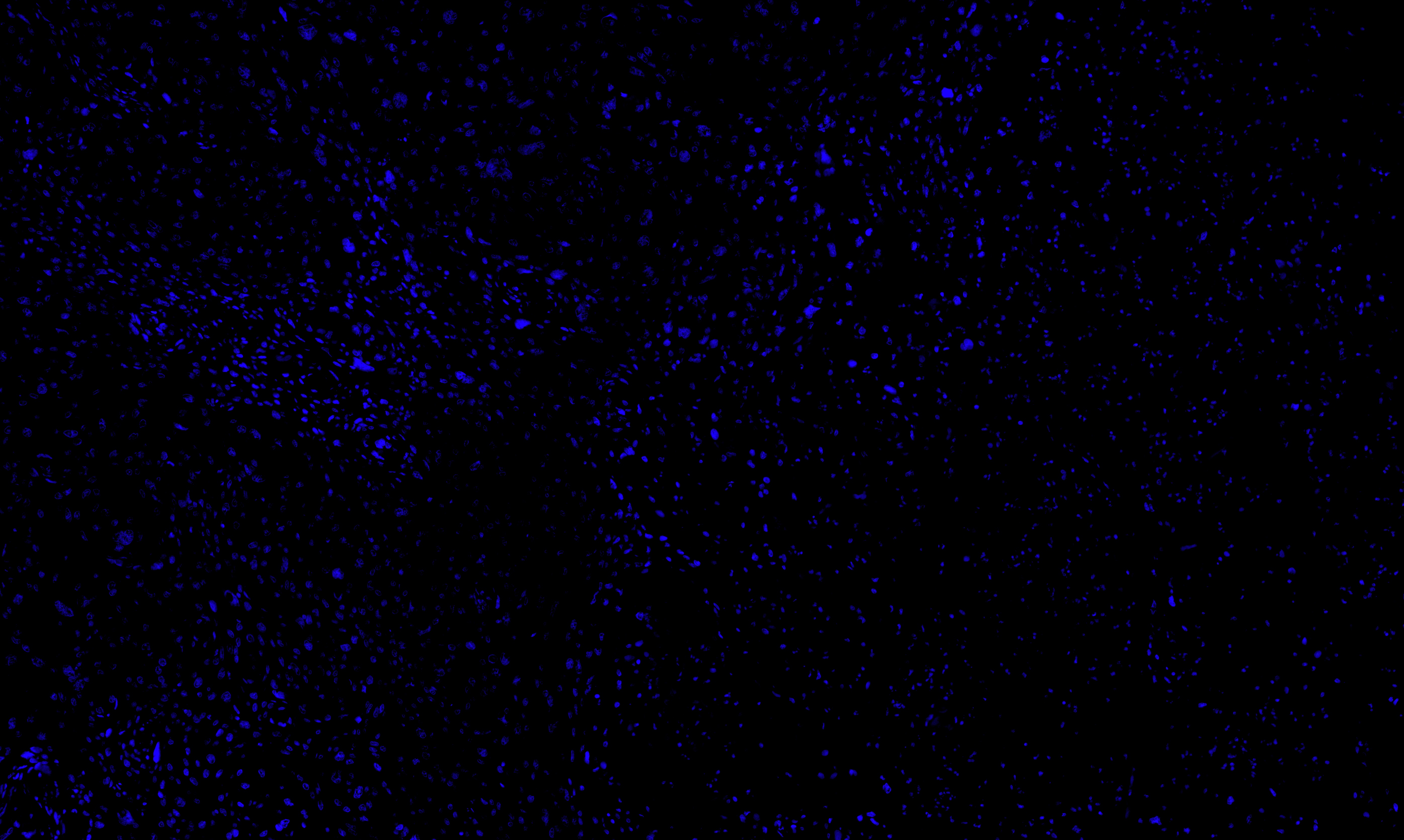

Supplement: Supplementary file 6 [file DataSheet5.zip › NETs-1/NETs_DAPI.jpg]

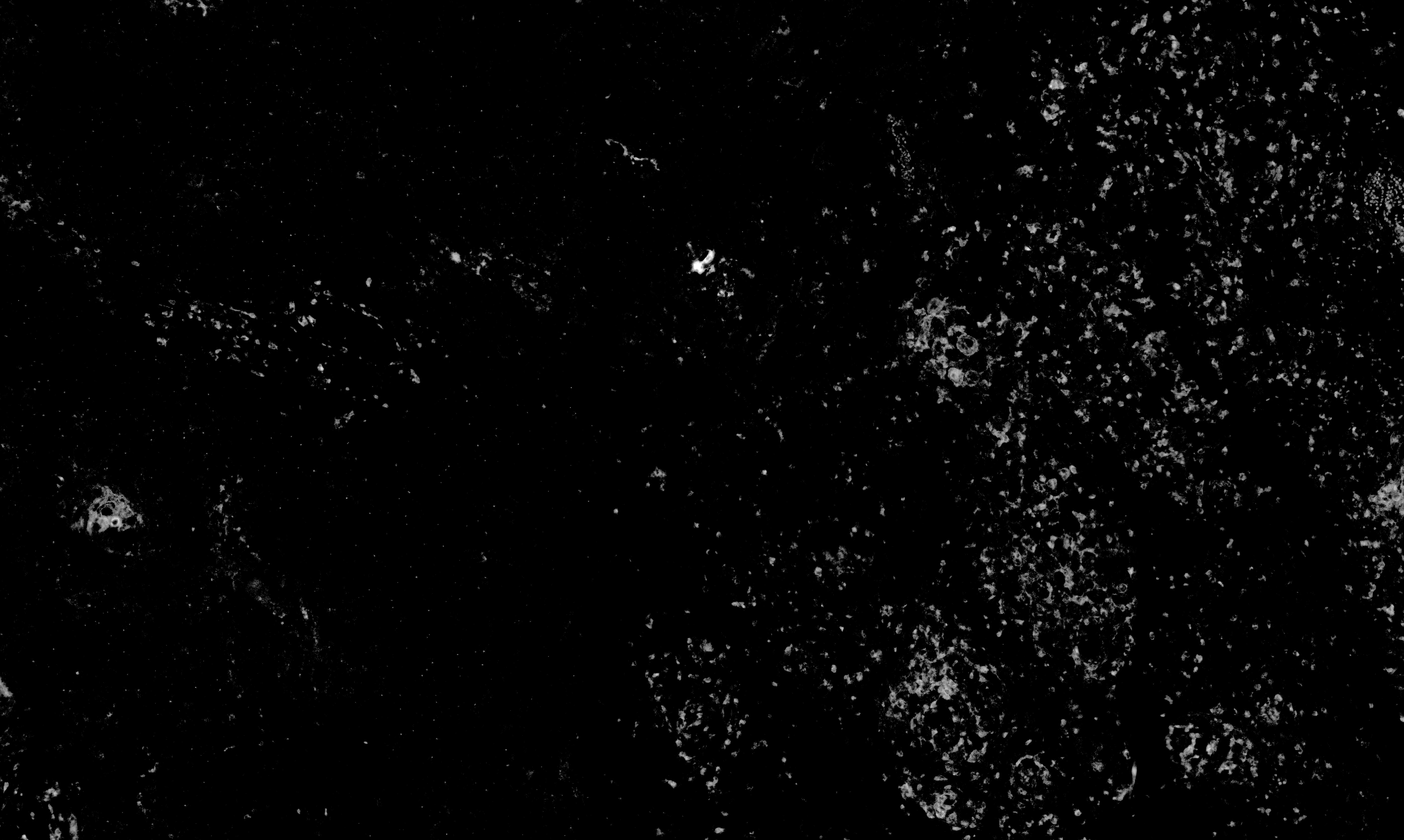

Supplement: Supplementary file 6 [file DataSheet5.zip › NETs-1/NETs_CXCR2.jpg]

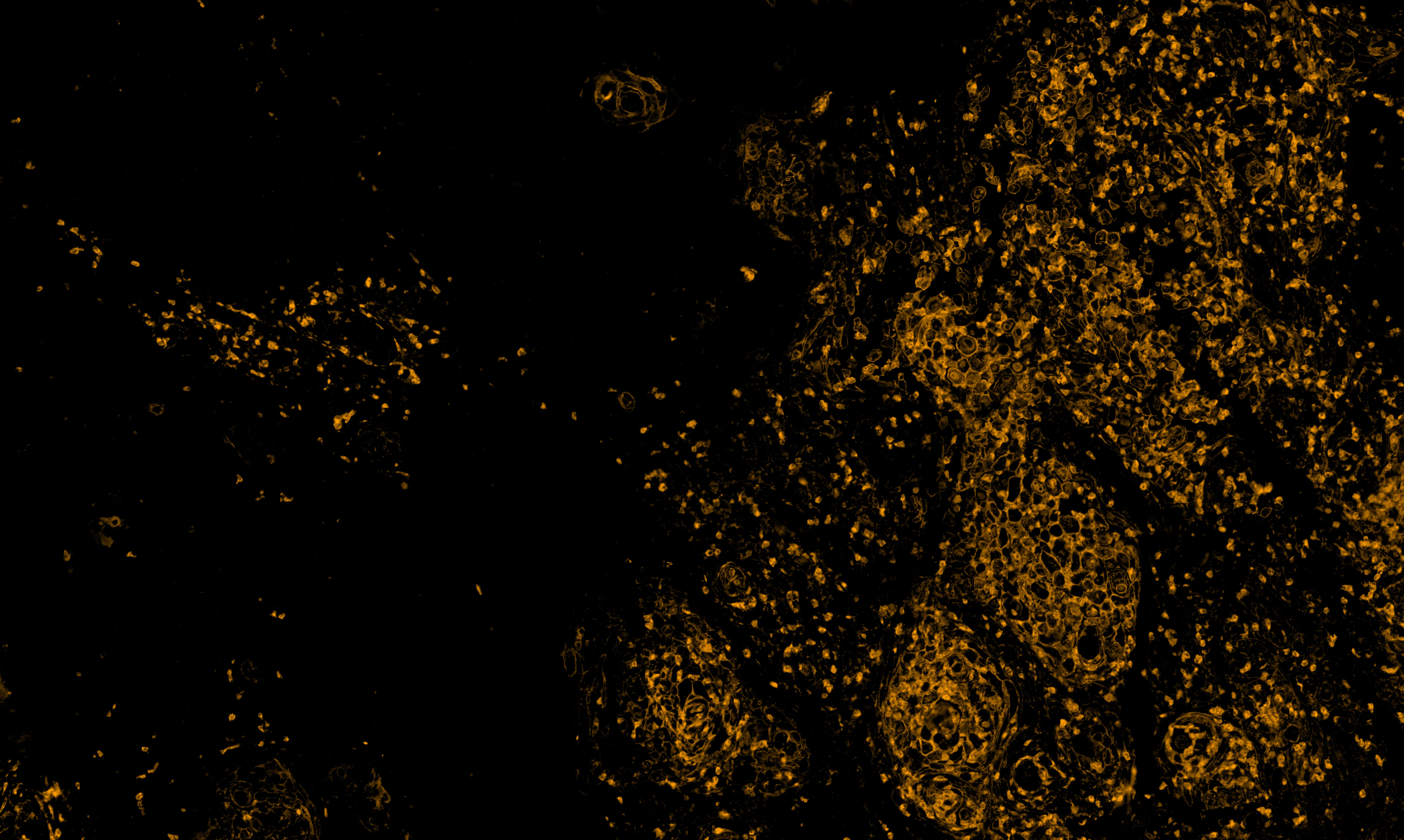

Supplement: Supplementary file 6 [file DataSheet5.zip › NETs-1/NETs_CD66b.jpg]

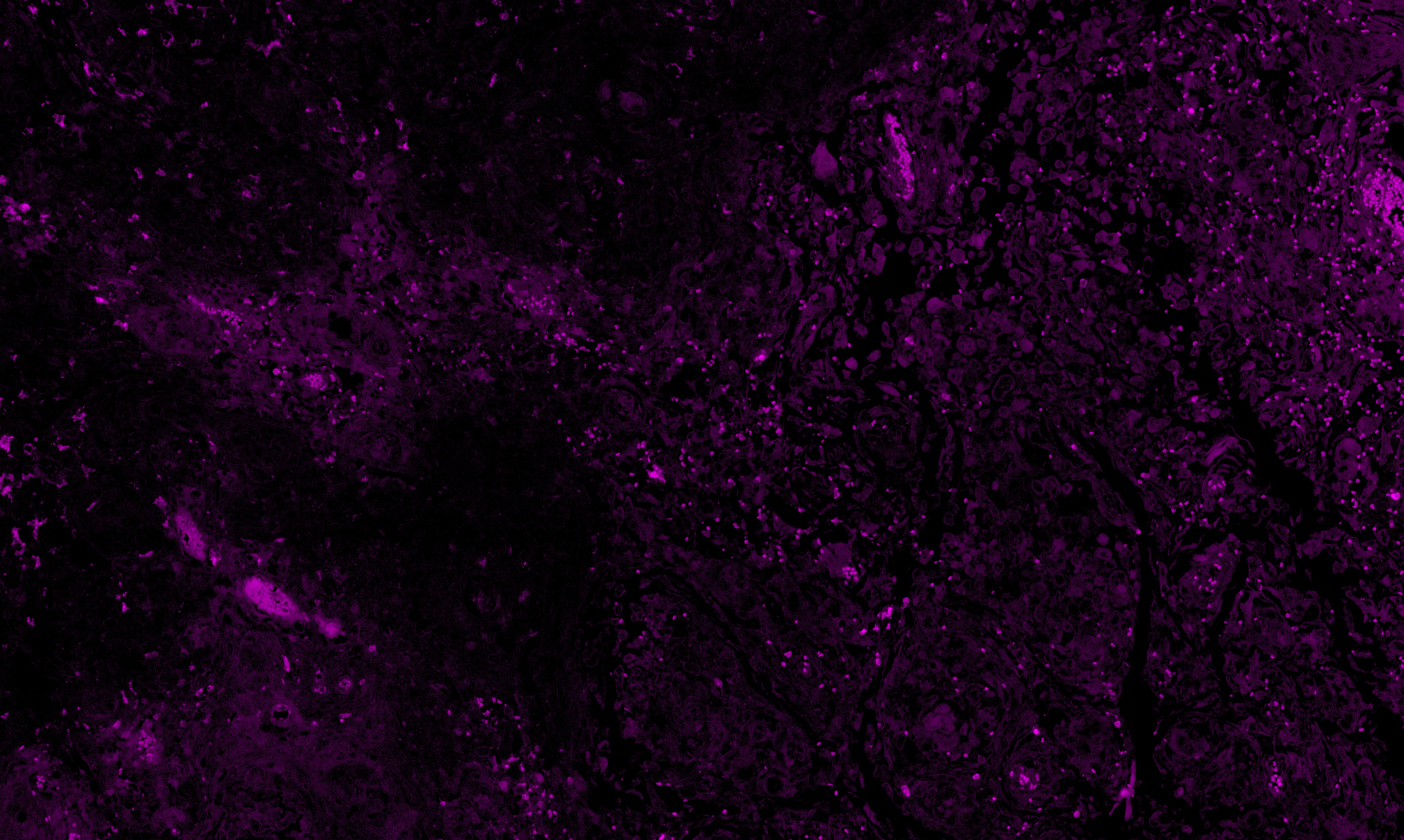

Supplement: Supplementary file 6 [file DataSheet5.zip › NETs-1/NETs_citH3.jpg]

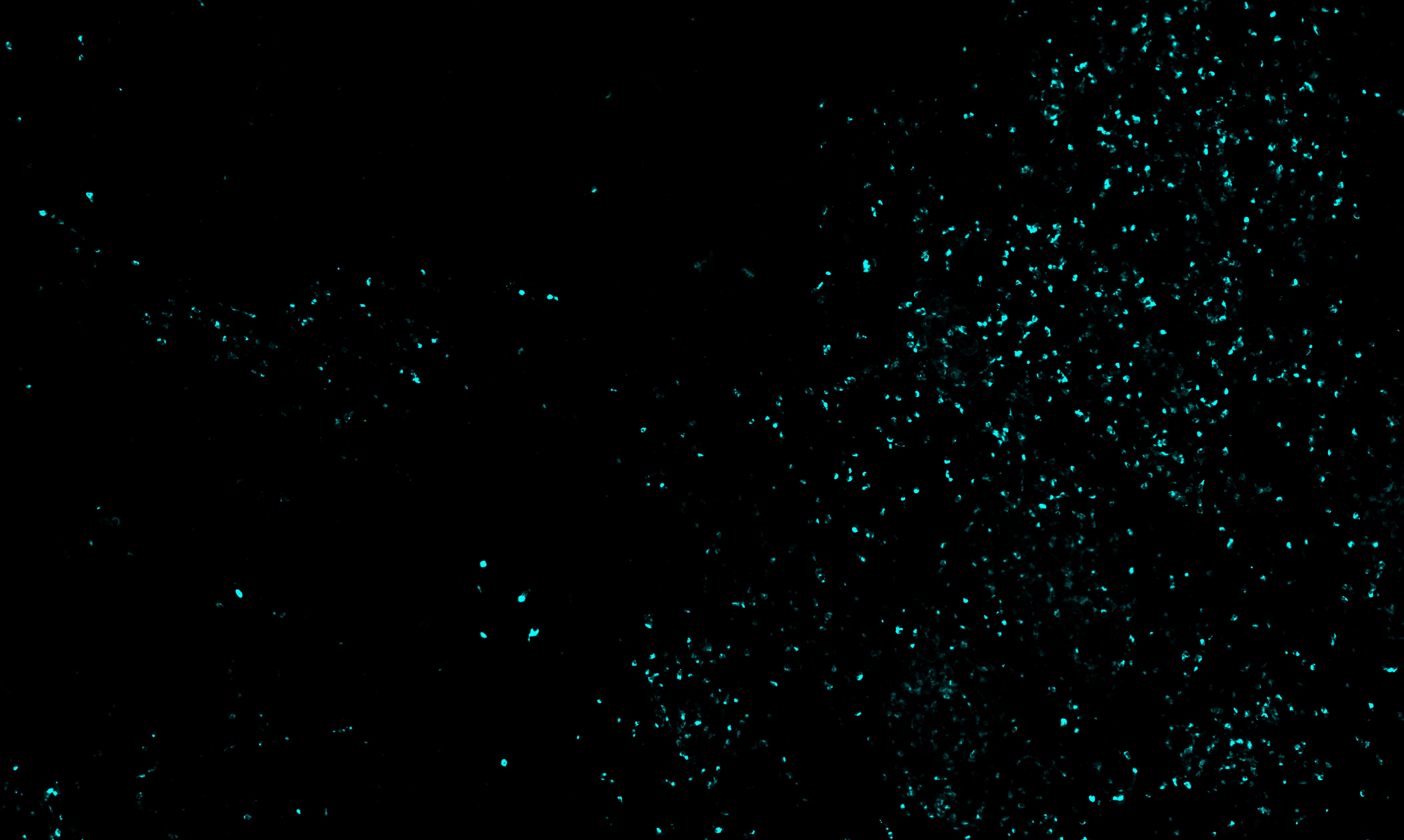

Supplement: Supplementary file 7 [file DataSheet6.zip › NETs-2/TGF-B.jpg]

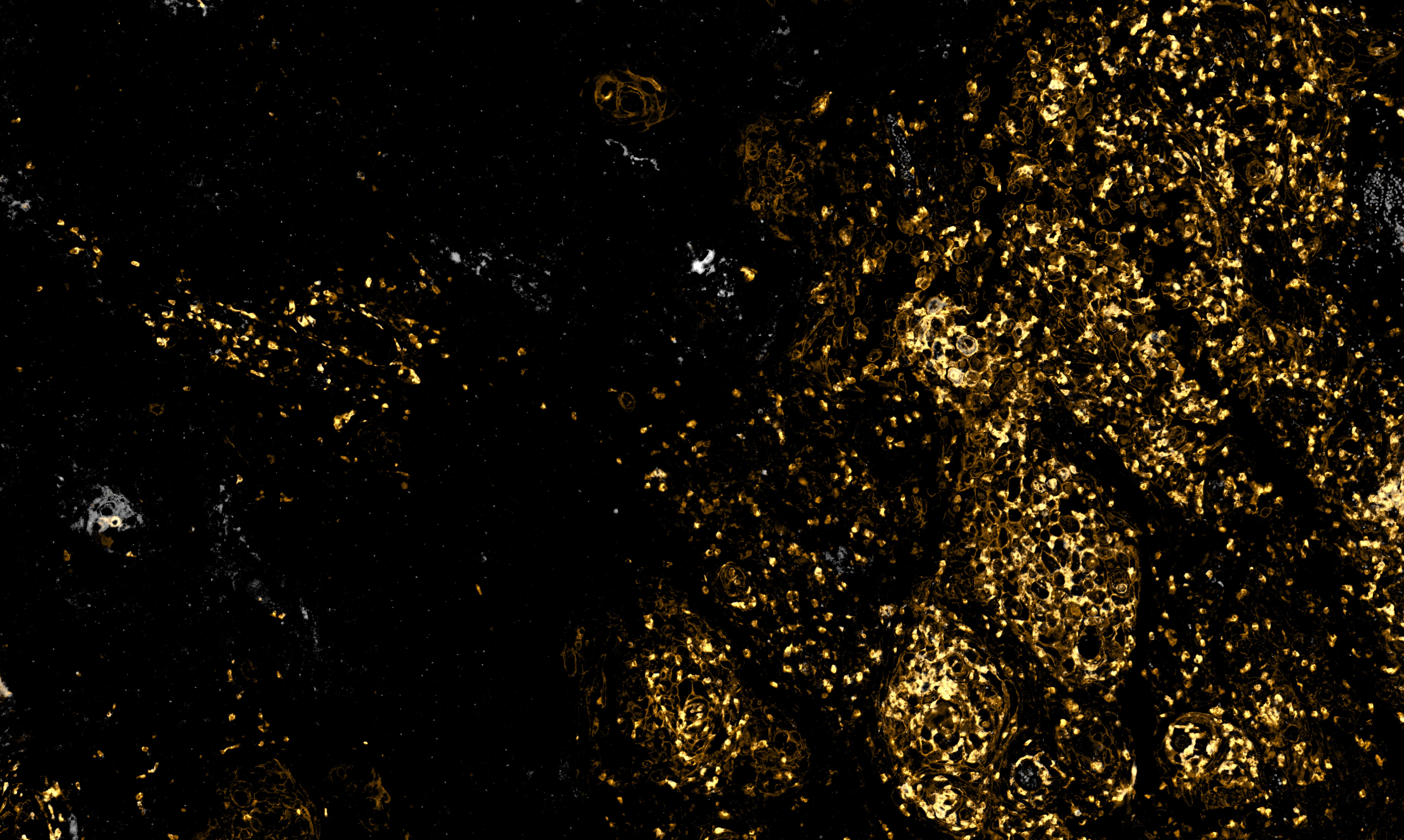

Supplement: Supplementary file 7 [file DataSheet6.zip › NETs-2/CXCR2TANs.jpg]

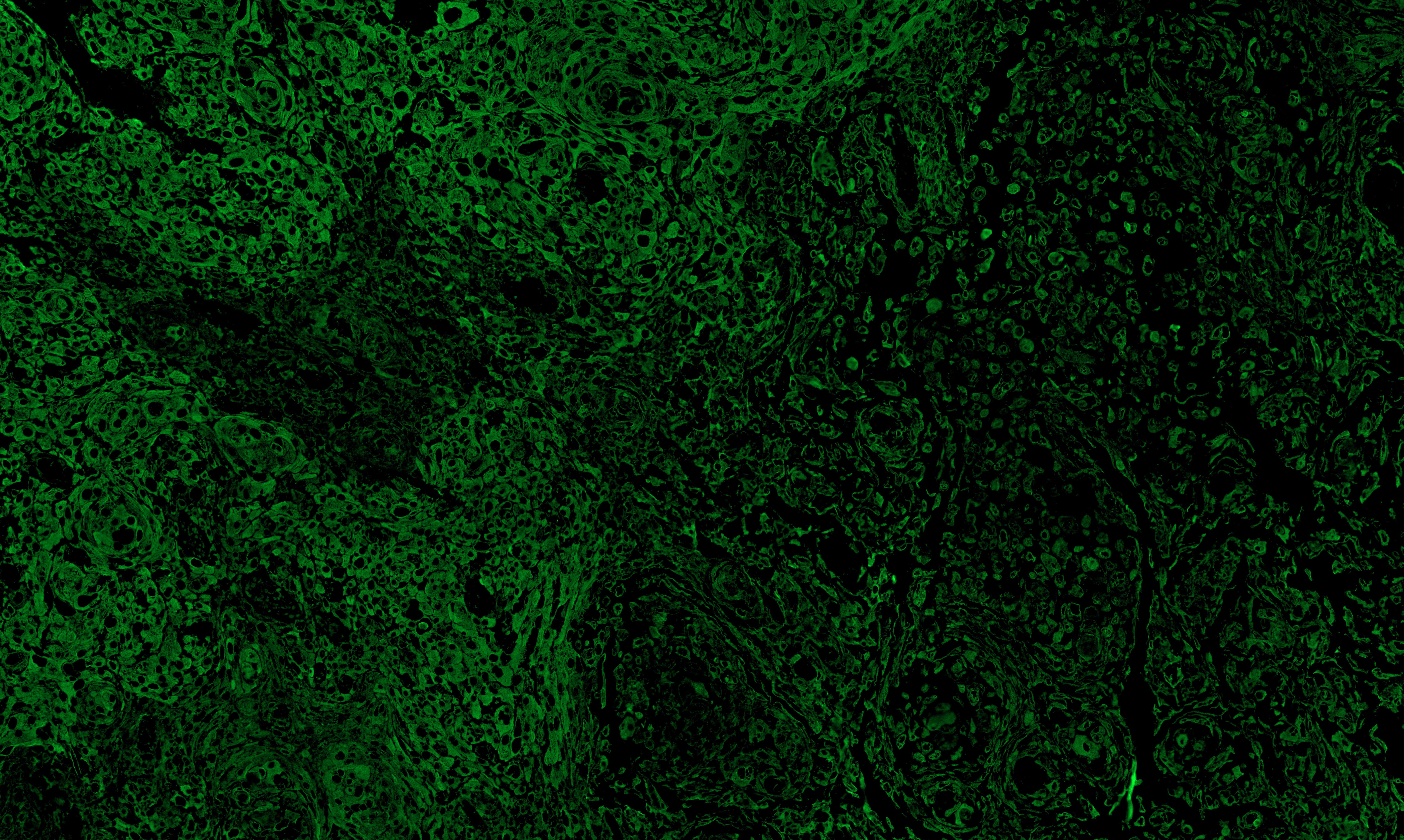

Supplement: Supplementary file 7 [file DataSheet6.zip › NETs-2/PAN-ck.jpg]

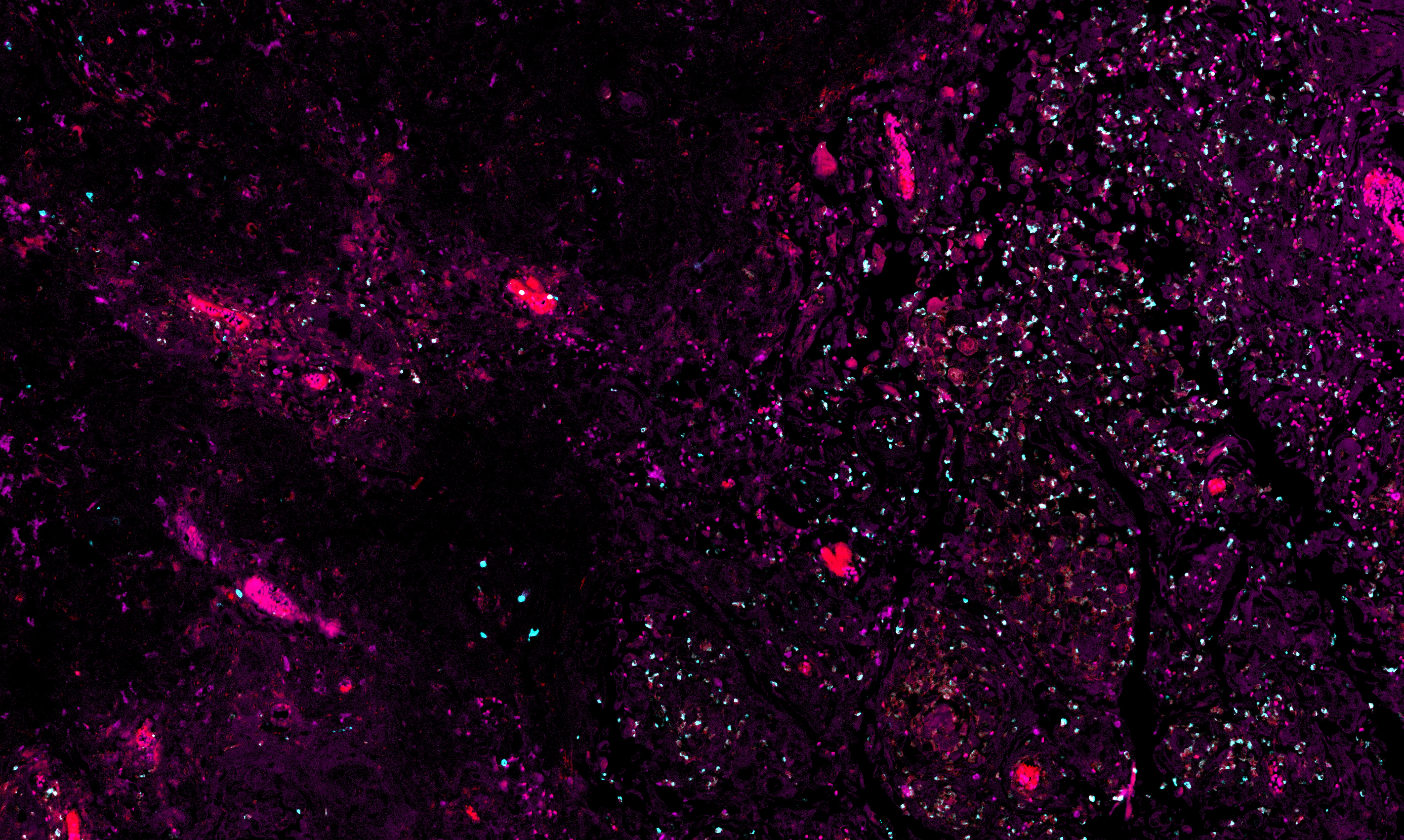

Supplement: Supplementary file 7 [file DataSheet6.zip › NETs-2/citH3MPOTGFB.jpg]
