# Supplementary material for: Neutrophil-to-lymphocyte ratio predicts a poor prognosis for penile cancer with an immunosuppressive tumor microenvironment
Source: Front Immunol. 2025 Apr 16;16:1568825. doi: 10.3389/fimmu.2025.1568825 (PMC12041217; doi:10.3389/fimmu.2025.1568825)
Supplement: Supplementary file 2 [file DataSheet1.pdf]

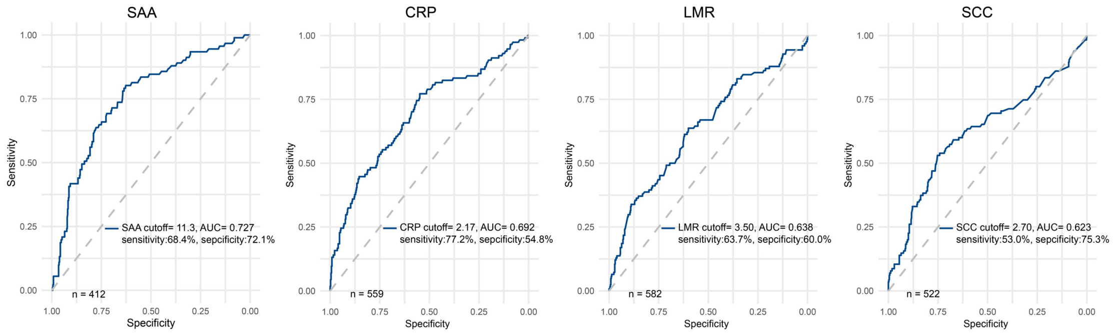

Figure. S1 The best cut-off value of clinical features by ROC curves.

ROC: receiver operating characteristic curve; SAA: serum amyloid A; CRP: C-reactive protein; LMR: lymphocyte-monocyte ratio; SCC-Ag: squamous cell carcinoma antigen; AUC: area under curve.

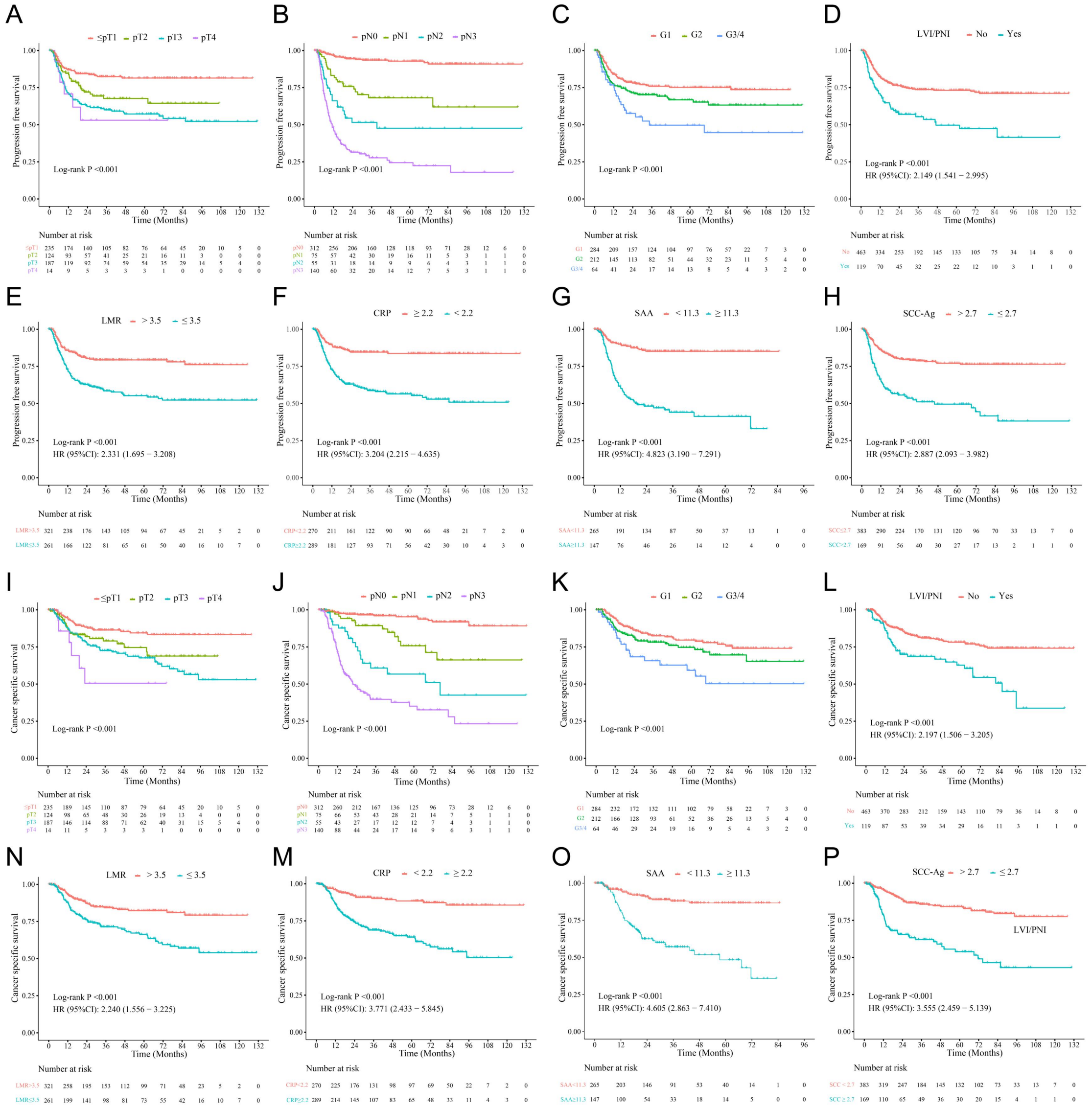

Figure. S2 Survival analysis of clinicopathological features in 582 PSCC patients. Kaplan-Meier survival analysis with log-rank test showed that advanced pT stage (A), pN stage (B), pathological grade (C), LVI/PNI (D), low LMR level (E), high CRP,

SAA and SCC-Ag level (F-H) were associated with poor progression free survival as well as cancer specific survival (I-P). HR: hazard ratio; Cl: confidence interval; LVI: lymphovascular invasion; PNI: perineural invasion; LMR: lymphocyte-monocyte ratio; CRP: C-reactive protein; SAA: serum amyloid A; SCC-Ag: squamous cell carcinoma antigen.

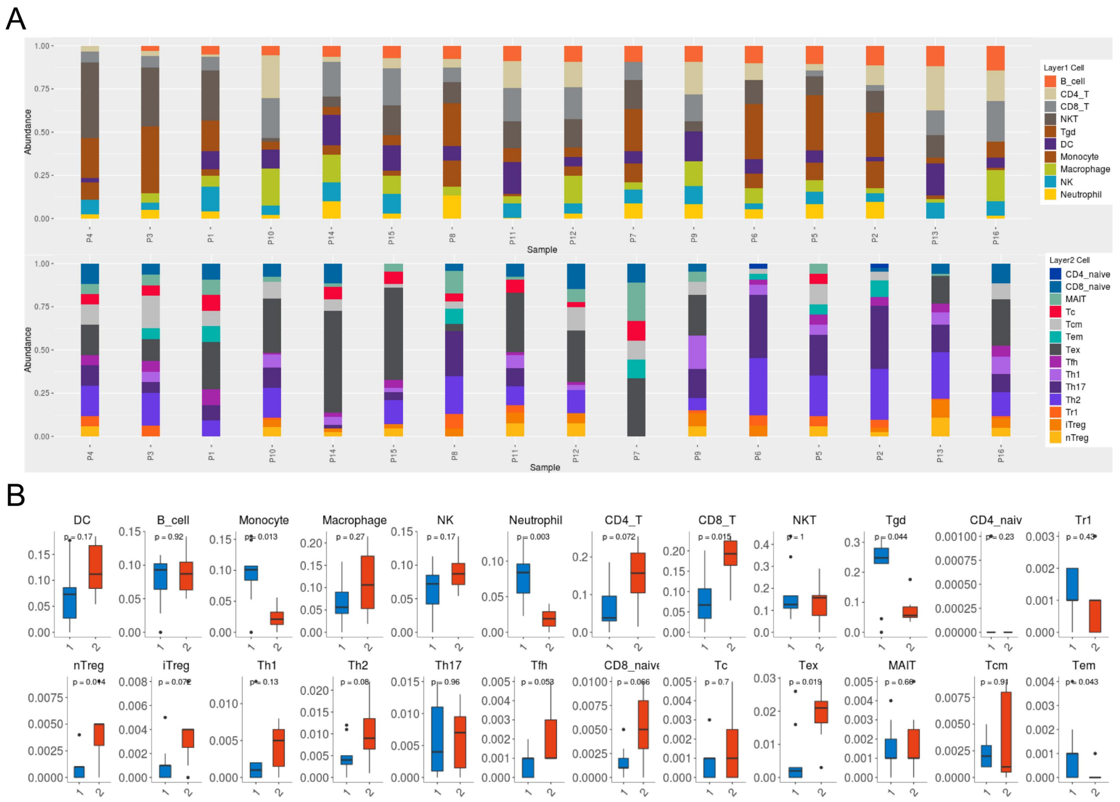

Figure. S3 The evaluation of tumor infiltrating immune cells between high and low NLR PSCC patients.

16 PSCC patients including nine high NLR (NLR≥ 3) level and seven low NLR (NLR< 3) level were performed with mRNA-seq to evaluation the infiltration scores by ImmuCellAI. (A) The bar chart showed the distribution of the 24 different immune cell types between two groups (high NLR group: P2, P3, P4, P5, P6, P7, P8, P9, P14; low NLR group: P1, P10, P11, P12, P13, P15, P16). (B) The box plots indicated the difference of immune cells between high and low NLR groups.

Supplementary Table 1. Univariate analysis of clinicopathological features associated with survival.

		PFS				CSS			
**Variables**	**n**	**3-year (95% Cl)**	**5-year (95% Cl)**	**HR (95% Cl)**	**P-value**	**3-year (95% Cl)**	**5-year (95% Cl)**	**HR (95% Cl)**	**P-value**
**Age (years)**					0.170				0.093
≤ 55	292	0.735 (0.682-0.788)	0.722 (0.667-0.777)	Ref.		0.799 (0.748-0.850)	0.785 (0.732-0.838)	Ref.	
> 55	290	0.661 (0.600-0.722)	0.643 (0.578-0.708)	1.24 (0.91-1.69)		0.772 (0.717-0.827)	0.709 (0.642-0.776)	1.36 (0.95-1.93)	
**BMI (kg/m^2)**					0.558				0.283
≤ 23	274	0.684 (0.625-0.743)	0.658 (0.593-0.723)	Ref.		0.780 (0.727-0.833)	0.709 (0.640-0.778)	Ref.	
> 23	266	0.698 (0.637-0.759)	0.698 (0.637-0.759)	0.91 (0.66-1.25)		0.771 (0.710-0.832)	0.771 (0.710-0.832)	0.82 (0.57-1.18)	
**Phimosis**					0.190				0.219
No	443	0.712 (0.665-0.759)	0.702 (0.655-0.749)	Ref.		0.795 (0.752-0.838)	0.771 (0.724-0.818)	Ref.	
Yes	139	0.661 (0.577-0.745)	0.638 (0.552-0.724)	1.26 (0.89-1.77)		0.752 (0.674-0.830)	0.697 (0.611-0.783)	1.27 (0.87-1.86)	
**Smoking**					0.469				0.419
No	275	0.687 (0.626-0.748)	0.669 (0.606-0.732)	Ref.		0.764 (0.705-0.823)	0.729 (0.664-0.794)	Ref.	
Yes	307	0.711 (0.656-0.766)	0.698 (0.641-0.755)	0.89 (0.66-1.22)		0.801 (0.752-0.850)	0.767 (0.712-0.822)	0.88 (0.63-1.21)	
**Diabetes**					0.705				0.475
No	538	0.701 (0.660-0.742)	0.685 (0.642-0.728)	Ref.		0.784 (0.745-0.823)	0.752 (0.709-0.795)	Ref.	
Yes	44	0.680 (0.511-0.849)	0.680 (0.511-0.849)	1.12 (0.63-1.97)		0.807 (0.678-0.936)	0.740 (0.568-0.912)	1.20 (0.62-1.25)	
**pT stage**					**<0.001**				**<0.001**
pTa/Tis/pT1	235	0.824 (0.771-0.877)	0.815 (0.760-0.870)	Ref.		0.864 (0.815-0.913)	0.845 (0.790-0.900)	Ref.	
pT2	124	0.677 (0.585-0.769)	0.677 (0.585-0.769)	1.90 (1.19-3.01)	**0.007**	0.790 (0.710-0.870)	0.746 (0.650-0.842)	1.87 (1.10-3.18)	**0.021**
pT3	187	0.600 (0.526-0.674)	0.572 (0.494-0.650)	2.74 (1.85-4.07)	**<0.001**	0.726 (0.655-0.797)	0.677 (0.599-0.755)	2.50 (1.60-3.92)	**<0.001**
pT4	14	0.530 (0.250-0.810)	0.530 (0.250-0.810)	3.14 (1.33-7.45)	**0.009**	0.505 (0.215-0.795)	0.505 (0.215-0.795)	4.45 (1.84-10.8)	**0.001**
**pN stage**					**<0.001**				**<0.001**
pN0	312	0.935 (0.906-0.964)	0.928 (0.895-0.961)	Ref.		0.960 (0.936-0.984)	0.953 (0.926-0.980)	Ref.	
pN1	75	0.682 (0.566-0.798)	0.682 (0.566-0.798)	5.06 (2.76-9.27)	**<0.001**	0.894 (0.820-0.968)	0.758 (0.631-0.885)	3.76 (1.83-7.72)	**<0.001**
pN2	55	0.516 (0.369-0.663)	0.476 (0.321-0.631)	9.63 (5.31-17.5)	**<0.001**	0.608 (0.453-0.763)	0.567 (0.404-0.730)	8.95 (4.59-17.4)	**<0.001**
pN3	140	0.277 (0.195-0.359)	0.245 (0.161-0.329)	18.6 (11.4-30.2)	**<0.001**	0.397 (0.301-0.493)	0.351 (0.247-0.455)	18.8 (10.9-32.5)	**<0.001**
**Grade**					**0.001**				**0.003**

G1	284	0.759 (0.704-0.814)	0.752 (0.697-0.807)	Ref.		0.825 (0.774-0.876)	0.796 (0.741-0.851)	Ref.	
G2	212	0.702 (0.637-0.767)	0.668 (0.595-0.741)	1.43 (1.01-2.04)	**0.045**	0.782 (0.721-0.843)	0.747 (0.676-0.818)	1.40 (0.94-2.08)	0.103
G3/4	64	0.497 (0.356-0.638)	0.497 (0.356-0.638)	2.37 (1.51-3.70)	**<0.001**	0.658 (0.527-0.789)	0.593 (0.446-0.740)	2.42 (1.46-4.00)	**0.001**
**LVI/PNI**					**<0.001**				**<0.001**
No	463	0.740 (0.697-0.783)	0.732 (0.687-0.777)	Ref.		0.810 (0.771-0.849)	0.782 (0.737-0.827)	Ref.	
Yes	119	0.554 (0.454-0.605)	0.497 (0.389-0.605)	2.15 (1.54-3.00)		0.686 (0.594-0.778)	0.627 (0.521-0.733)	2.20 (1.51-3.20)	
**NLR**					**<0.001**				**<0.001**
< 3.0	356	0.819 (0.776-0.862)	0.814 (0.771-0.857)	Ref.		0.866 (0.827-0.905)	0.854 (0.813-0.895)	Ref.	
≥ 3.0	226	0.505 (0.431-0.538)	0.458 (0.378-0.538)	3.24 (2.36-4.46)		0.652 (0.579-0.725)	0.574 (0.490-0.658)	3.45 (2.39-4.98)	
**LMR**					**<0.001**				**<0.001**
> 3.5	321	0.794 (0.747-0.841)	0.794 (0.747-0.841)	Ref.		0.843 (0.798-0.888)	0.823 (0.774-0.872)	Ref.	
≤ 3.5	261	0.585 (0.520-0.650)	0.553 (0.484-0.622)	2.33 (1.69-3.21)		0.714 (0.653-0.775)	0.663 (0.579-0.747)	2.24 (1.56-3.23)	
**CRP (mg/L)**					**<0.001**				**<0.001**
< 2.2	270	0.846 (0.801-0.891)	0.837 (0.788-0.886)	Ref.		0.901 (0.862-0.940)	0.884 (0.839-0.929)	Ref.	
≥ 2.2	289	0.587 (0.524-0.650)	0.564 (0.499-0.629)	3.20 (2.21-4.63)		0.688 (0.627-0.749)	0.641 (0.574-0.708)	3.77 (2.43-5.84)	
**SAA (mg/L)**					**<0.001**				**<0.001**
< 11.3	265	0.852 (0.805-0.899)	0.852 (0.805-0.899)	Ref.		0.880 (0.833-0.927)	0.868 (0.817-0.919)	Ref.	
≥ 11.3	147	0.441 (0.349-0.533)	0.413 (0.313-0.513)	4.82 (3.19-7.28)		0.571 (0.475-0.667)	0.484 (0.361-0.607)	4.60 (2.86-7.41)	
**SCC-Ag (ng/ml)**					**<0.001**				**<0.001**
< 2.7	383	0.788 (0.745-0.831)	0.771 (0.724-0.818)	Ref.		0.859 (0.820-0.898)	0.843 (0.800-0.886)	Ref.	
≥ 2.7	169	0.511 (0.425-0.597)	0.495 (0.405-0.585)	2.89 (2.09-3.98)		0.621 (0.537-0.705)	0.539 (0.439-0.639)	3.55 (2.46-5.14)	

BMI: body mass index; LVI: lymphovascular invasion; PNI: perineural invasion; NLR: neutrophil-lymphocyte ratio; LMR: lymphocyte-monocyte ratio; CRP: C-reactive protein; SAA: serum amyloid A; SCC-Ag: squamous cell carcinoma antigen; Ref.: Reference; PFS: progression free survival; CSS: cancer specific survival; HR: hazard ratio; 95% Cl: 95% confidence interval.

Supplementary Table 2. Multivariate analysis of factors associated with survival.

	PFS		CSS	
**Variables**	**HR (95% Cl)**	**P-value**	**HR (95% Cl)**	**P-value**
**pT stage**		0.834		0.792
pTa/Tis/pT1	Ref.		Ref.	
pT2	1.13 (0.62-2.06)	0.688	1.29 (0.65-2.58)	0.464
pT3	1.08 (0.63-1.85)	0.778	0.96 (0.52-1.76)	0.884
pT4	0.72 (0.26-2.00)	0.532	1.06 (0.37-3.06)	0.913
**pN stage**		**<0.001**		**<0.001**
pN0	Ref.		Ref.	
pN1	2.99 (1.07-8.39)	**0.038**	1.24 (0.33-4.67)	0.749
pN2	9.62 (4.10-22.6)	**<0.001**	9.10 (3.61-22.9)	**<0.001**
pN3	13.8 (6.79-28.0)	**<0.001**	10.3 (4.86-22.0)	**<0.001**
**Grade**		0.594		0.636
G1	Ref.		Ref.	
G2	0.80 (0.51-1.27)	0.351	0.79 (0.55-1.57)	0.788
G3/4	0.99 (0.74-2.56)	0.983	1.29 (0.63-2.64)	0.480
**LVI/PNI (Yes vs No)**	1.28 (0.81-2.00)	0.289	1.29 (0.77-2.16)	0.332
**NLR (≥ 3.0 vs < 3.0)**	1.01 (0.54-1.89)	0.972	0.96 (0.47-1.95)	0.903
**LMR (≤ 3.5 vs > 3.5)**	1.32 (0.76-2.29)	0.324	1.32 (0.71-2.44)	0.383
**CRP (≥ 2.2 vs < 2.2 mg/L)**	1.38 (0.74-2.56)	0.316	1.74 (0.86-3.51)	0.125
**SAA (≥ 11.3 vs < 11.3 mg/L)**	2.17 (1.18-3.99)	**0.012**	1.63 (0.82-3.25)	0.162
**SCC-Ag (≥ 2.7 vs < 2.7 ng/ml)**	0.92 (0.58-1.45)	0.708	1.20 (0.71-2.02)	0.505

PFS: progression free survival; CSS: cancer specific survival; HR: hazard ratio; 95% Cl: 95% confidence interval; LVI: lymphovascular invasion; PNI: perineural invasion; NLR: neutrophil-lymphocyte ratio; LMR: lymphocyte-monocyte ratio; CRP: C-reactive protein; SAA: serum amyloid A; SCC-Ag: squamous cell carcinoma antigen; Ref.: Reference
